# Epigenetic modifications precede molecular alterations and drive human hepatocarcinogenesis

**DOI:** 10.1172/jci.insight.146196

**Published:** 2021-09-08

**Authors:** Carolin Czauderna, Alicia Poplawski, Colm J. O’Rourke, Darko Castven, Benjamín Pérez-Aguilar, Diana Becker, Stephanie Heilmann-Heimbach, Margarete Odenthal, Wafa Amer, Marcel Schmiel, Uta Drebber, Harald Binder, Dirk A. Ridder, Mario Schindeldecker, Beate K. Straub, Peter R. Galle, Jesper B. Andersen, Snorri S. Thorgeirsson, Young Nyun Park, Jens U. Marquardt

**Affiliations:** 1 Department of Medicine I, University Medical Center Mainz, Mainz, Germany.; 2 Department of Medicine I, University Medical Center Schleswig-Holstein, Campus Lübeck, Lübeck, Germany.; 3 Institute of Medical Biostatistics, Epidemiology, and Informatics (IMBEI), Division Biostatistics and Bioinformatics, Johannes Gutenberg University, Mainz, Germany.; 4 Biotech Research and Innovation Centre (BRIC), Department of Health and Medical Sciences, University of Copenhagen, Copenhagen, Denmark.; 5 Institute of Human Genetics, Department of Genomics, Life & Brain Center, University of Bonn, Bonn, Germany.; 6 Institute of Pathology, University Clinic of Cologne, Center for Molecular Medicine Cologne, University of Cologne, Cologne, Germany.; 7 Faculty of Medicine and Medical Center, University of Freiburg, Institute of Medical Biometry and Statistics, Freiburg, Germany.; 8 Department of Pathology, University Medical Center Mainz, Mainz, Germany.; 9 Tissue Bank, University Medical Center Mainz, Mainz, Germany.; 10 Laboratory of Experimental Carcinogenesis (LEC), Center for Cancer Research, National Cancer Institute, NIH, Washington DC, USA.; 11 Department of Pathology, Brain Korea; 21 Project for Medical Science, Integrated Genomic Research Center for Metabolic Regulation, Yonsei University, Seoul, Republic of Korea.

**Keywords:** Hepatology, Oncology, Epigenetics, Liver cancer, Molecular biology

## Abstract

Development of primary liver cancer is a multistage process. Detailed understanding of sequential epigenetic alterations is largely missing. Here, we performed Infinium Human Methylation 450k BeadChips and RNA-Seq analyses for genome-wide methylome and transcriptome profiling of cirrhotic liver (*n* = 7), low- (*n* = 4) and high-grade (*n* = 9) dysplastic lesions, and early (*n* = 5) and progressed (*n* = 3) hepatocellular carcinomas (HCC) synchronously detected in 8 patients with HCC with chronic hepatitis B infection. Integrative analyses of epigenetically driven molecular changes were identified and validated in 2 independent cohorts comprising 887 HCCs. Mitochondrial DNA sequencing was further employed for clonality analyses, indicating multiclonal origin in the majority of investigated HCCs. Alterations in DNA methylation progressively increased from liver cirrhosis (CL) to dysplastic lesions and reached a maximum in early HCCs. Associated early alterations identified by Ingenuity Pathway Analysis (IPA) involved apoptosis, immune regulation, and stemness pathways, while late changes centered on cell survival, proliferation, and invasion. We further validated 23 putative epidrivers with concomitant expression changes and associated with overall survival. Functionally, Striatin 4 (STRN4) was demonstrated to be epigenetically regulated, and inhibition of STRN4 significantly suppressed tumorigenicity of HCC cell lines. Overall, application of integrative genomic analyses defines epigenetic driver alterations and provides promising targets for potentially novel therapeutic approaches.

## Graphical abstract

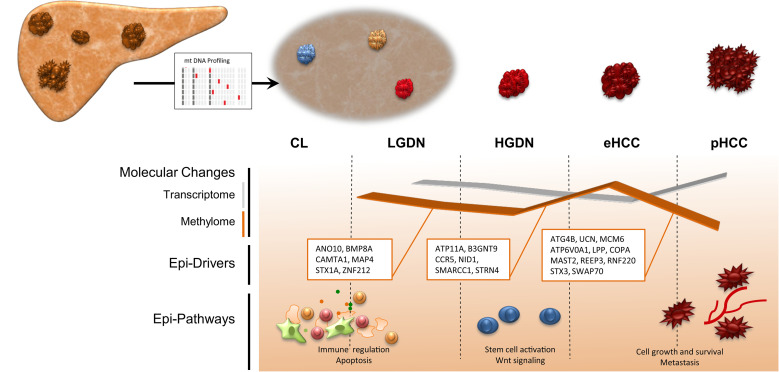

## Introduction

Hepatocellular carcinoma (HCC) is a hallmark of inflammation-induced cancers and ranks among the most common causes of cancer-related deaths worldwide (1). Herein, hepatocarcinogenesis is a multistage process that most frequently develops in the background of a chronic inflammatory liver disease and liver cirrhosis (CL) induced by chronic viral hepatitis, either hepatitis B (HBV) or C viruses (HCV), alcohol abuse, or other metabolic and hereditary factors (2). Preneoplastic dysplastic lesions, i.e., low-grade dysplastic nodules (LGDNs) and high-grade dysplastic nodules (HGDNs), evolve into early hepatocellular carcinoma (eHCC) that, subsequently, progresses to advanced HCC (pHCC) (3). This sequence is accelerated by genetic and epigenetic alterations that induce a malignant transformation at early stages and promote progression into advanced stages. During the past decade, several molecular alterations and changes to the microenvironmental cellular contexture have been associated with increased risk of HCC in chronic liver diseases (3, 4). Integrative transcriptome analysis of dysplastic lesions, eHCC, and pHCC in patients with chronic HBV infection recently revealed that molecular profiles of early lesions are relatively uniform, whereas a sharp increase in molecular heterogeneity is induced in pHCC (5). However, activation of prognostically adverse signaling pathways from dysplastic nodules (DNs) to pHCC was only partially explained by observed genetic alterations, suggesting that complementary mechanisms might be operative and drive hepatocarcinogenesis. It is well established that epigenetic mechanisms in cancer cells are highly influenced by microenvironmental stimuli. In this context, changes in DNA methylation patterns are believed to be early events in tumor development in inflammatory cancers preceding allelic imbalances and ultimately leading to cancer progression, thereby adding considerable complexity to the pathogenesis of liver and other cancers (6, 7). In the liver, methylation patterns can be effectively used to classify patients according to different etiological factors (e.g., HBV, HCV, alcohol) (8). In addition to changes in global methylation patterns, distinct methylation profiles are strongly correlated with clinical characteristics and survival of patients with HCC (9–13). Recent studies also indicate that methylation signatures have a high prognostic value for HCC development and recurrence after curative resection or liver transplantation (12, 14, 15). Evidence for the importance of a DNA methylation dependent, multistep sequence of molecular alterations in hepatocarcinogenesis was further demonstrated in HBV-related liver cancers and indicated a major contribution of epigenetics in deregulation of key pro-oncogenic molecules from cirrhotic nodules over DNs to eHCC and finally pHCC (16). However, our understanding of the molecular complexity is still limited; a detailed catalogue of key (epi)genetic alterations commonly altered across the full spectrum of hepatocarcinogenesis remains to be defined. This lack of information represents a major challenge for preventive strategies as well as therapeutic approaches in HCC.

A significant drawback in the study of sequential evolution of liver cancer is the scarcity of tissues from early stages. In contrast to other cancers, detailed investigation of stage-wise progression is also highly demanding due to a shortage of the available lesions from the full spectrum of stages from livers of individual patients. To overcome this limitation, we here collected a cohort of unique patients infected with HBV with synchronous occurrence of the complete spectrum of early and advanced stages in the same patient and performed multilevel sequencing analyses. Interestingly, our mitochondrial DNA sequencing (mtDNA-Seq) analyses revealed a multiclonal origin of codeveloped lesions in the background of chronic liver inflammation. We further created a detailed landscape of epigenetic alterations and affected signaling pathways in HCC. New epigenetic drivers were identified by integrative approaches and subsequently validated in 2 Western cohorts of patients with HCC comprising 887 human samples. Notably, 23 newly identified and validated epidrivers have an impact on overall survival of patients with HCC. Among them, Striatin 4 (*STRN4*) was shown to be epigenetically regulated and highly activated in late stages of hepatocarcinogenesis with prognostically adverse implications for patients with HCC. Here we demonstrate that targeting STRN4 resulted in decreased tumorigenicity of liver cancer cells.

## Results

### Clonal diversity of (pre)neoplastic lesions.

Several recent studies indicate substantial intratumoral heterogeneity in HCC (17). The presented cohort comprising low- (*n* = 4) and high-grade (*n* = 9) dysplastic lesions, eHCC (*n* = 5), and pHCC (*n* = 3) as well as cirrhotic liver (*n* = 7) from 8 individual patients with HCC offers a unique background to delineate if synchronously coexisting preneoplastic and cancerous lesions are derived from the same clonal origin within individual livers. Clinicopathological characteristics of the patients are displayed in Table 1. Mitochondria are highly exposed to ROS (18). Consequently, mt-genome integrity is significantly disrupted during tumor development, leading to clonal expansion or loss of mutated mtDNA copies. Thus, tracking of mt-genome variants, particularly heteroplasmic mt-variants, effectively defines the clonal origin of different lesions (19).

**Figure 1:**
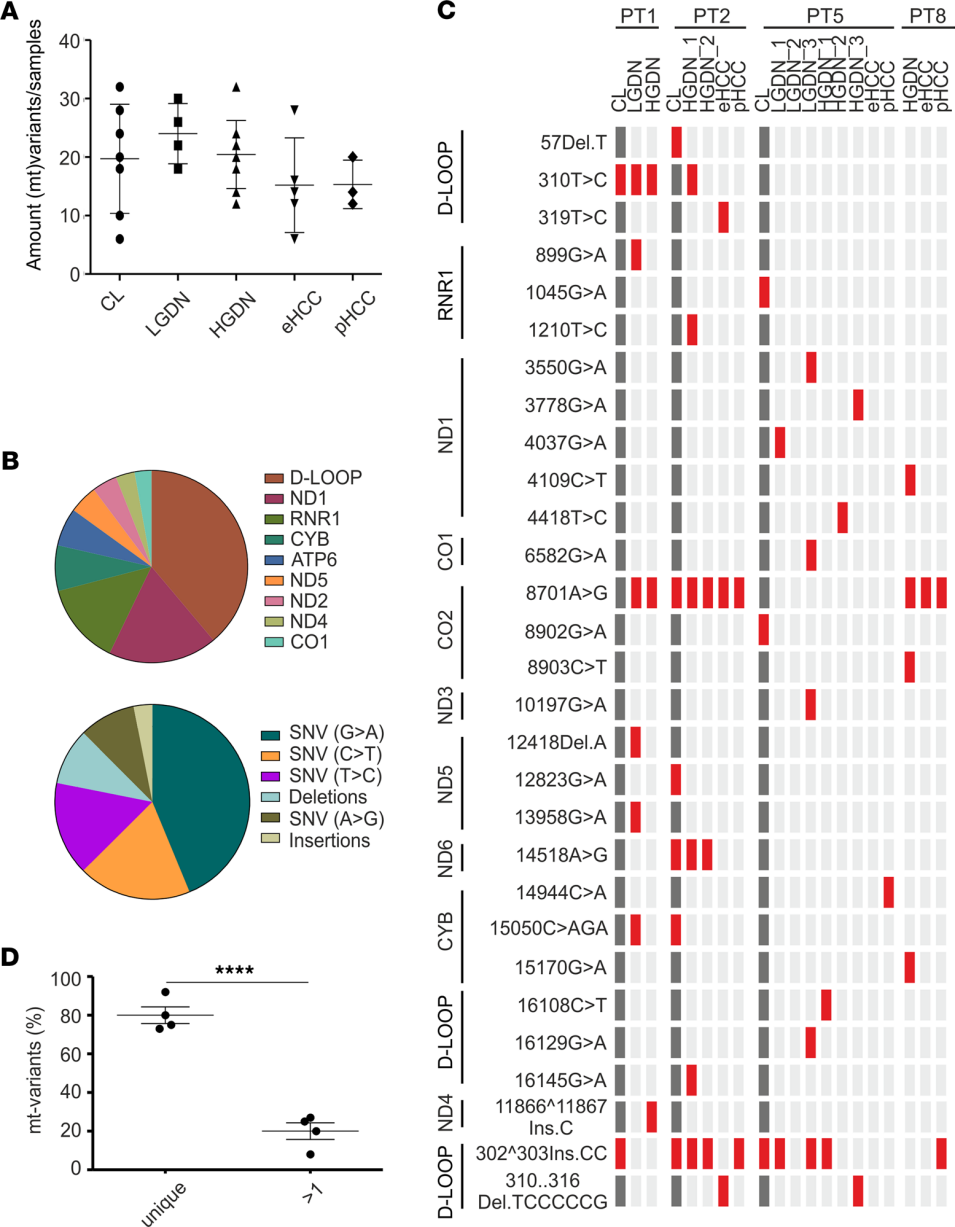
mtDNA-seq for clonality analyses. (**A**) Shown is the amount of mt-variants per sample (mean ± SD), CL, *n* = 7; LGDN, *n* = 4; HGDN, *n* = 9; eHCC, *n* = 5; and pHCC, *n* = 3; 1-way ANOVA (Bonferroni Correction). (**B**) Frequency of mt-variants in mt-gene regions (up) and type of mt-variants (down) normalized to CL and base pair length cumulatively detected in preneoplastic and cancer lesions. (**C**) Mt-variant–profiling of PT1, PT2, PT5, and PT8. Red: heteroplasmic mt-variants. (**D**) Shown is the occurrence of mt-variants in the lesions per patient as a percent divided in unique occurrence versus presence in more than 1 lesion. Students *t* test, ****P < 0.0001.****

We applied mtDNA-seq to the entire cohort and identified a total of 830 mt-variants. A median of 29.5 mt-variants were detected in individual samples (CL: 30 ± 4.7; LGDN: 32 ± 2.6; HGDN: 30 ± 2.9; eHCC: 28 ± 4.0; pHCC: 28 ± 2.1). The overall number of mt-variants were comparable across all stages of the disease, indicating that a high mt-mutation rate is already present in the HBV-infected, diseased cirrhotic livers (Figure 1A). As expected, most variants were homoplasmic (735 of 830) and the highest frequency of variants in preneoplastic and cancer lesions was observed in the D-LOOP region, i.e., involved in genes important for replication and expression of mtDNA (Figure 1B) (19, 20). Most of the alterations were single nucleotide variations with G>A base transitions (Figure 1B). Notably, several mt-variants have been associated with other cancer types (Supplemental Table 1; supplemental material available online with this article; https://doi.org/10.1172/jci.insight.146196DS1). After exclusion of patients with only 1 lesion (i.e., PT3, PT4, PT6, and PT7), mt-variant profiles were generated based on heteroplasmic mt-variants for PT1, PT2, PT5, and PT8 (Figure 1C, Supplemental Table 2, and Supplemental Figure 1A).

Interestingly, LGDNs and HGDNs of PT1 had 2 common heteroplasmic mt-variants (*310T > C; 8701A > G*), suggesting that lesions could either have evolved from clonal expansion and share the same cellular origin or be associated with a malignant transformation in general (21, 22). However, we detected several heteroplasmic mt-variants that occurred only in the LGDNs and, consistently, mutational profiles of preneoplastic and cancer lesions of PT2, PT5, and PT8 were highly heterogeneous, indicating a multiclonal origin in the majority of lesions (Figure 1C). We further confirmed that 20% (± 8.52) of variants were present in more than 1 lesion, whereas 80% (± 8.52) were unique variants (*P* < 0.0001; Figure 1D) overall, suggesting that the mutational profiles of the different lesions are driven by de novo emergence in individual lesions (Figure 1C). These observations suggest that the diseased hepatic microenvironment might induce a field effect that predisposes induction of epigenetic and genetic changes throughout the liver, resulting in multiple preneoplastic lesions gradually progressing to advanced HCC (17, 23). These findings prompted us to next dissect the epigenetic signature and the resulting transcriptomic changes during sequential evolution of liver cancer.

### Epigenetic landscape during sequential evolution of HCC.

**Figure 2:**
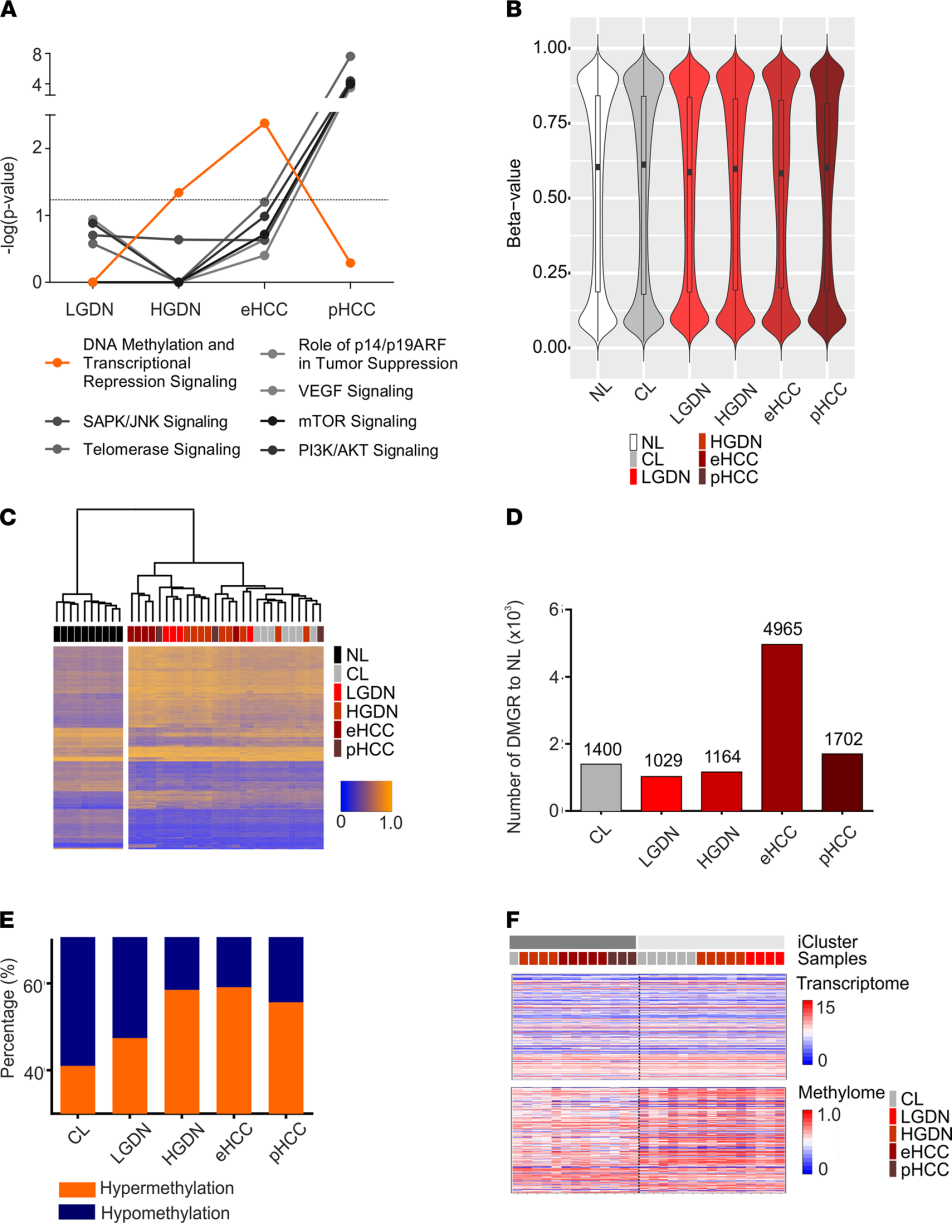
Epigenetic landscape of hepatocarcinogenesis. (**A**) Signaling pathway regulation during sequential evolution of HCC analyzed by ingenuity pathway analyses based on differentially expressed genes (DEG). DEGs were identified by DESeq2 in R using Wald Test statistics. Significance of each pathway was determined by scoring system provided by the IPA tool. (**B**) Shown are violin blots demonstrating methylation variance and β-value distribution during malignant transformation. (**C**) Unsupervised clustering of DMGRs of CL, preneoplastic (LGDN, HGDN), and cancer (eHCC, pHCC) lesions to noninfected NLs; FDR-corrected *P* value < 0.05 and/or a minimum β-value difference ≥ ± 0.2 (see Methods). (**D**) Number of DMGRs to NL during sequential evolution of HCC; FDR-corrected *P* value < 0.05 and/or a minimum β-value difference ≥ ± 0.2. (**E**) Distribution of hyper- (orange) and hypomethylated (blue) DMGRs during sequential evolution of HCC. (**F**) Heatmap organized by iCluster grouping DNA-methylation status and mRNA expression.

We first assessed global transcriptome changes as well as significantly deregulated signaling pathways of the different lesions. The results confirmed our previous findings that activation of key oncogenic pathways occurred late, i.e., in eHCC and pHCC lesions (5). However, when analyzing networks related to epigenetic modifications, we detected a significant activation of ‘genes related to DNA methylation and transcriptional repression signaling’ that occurred early during malignant transformation with a peak in DNs and eHCC. Interestingly, pathways associated with epigenetic changes were largely inactivated in pHCC, suggesting that mechanisms beyond epigenetics might be operative at advanced stages (Figure 2A and Supplemental Table 3). Next, we sought to define and quantify global methylation patterns affected during hepatocarcinogenesis. We applied Infinium Human Methylation 450k BeadChips to all lesions. As already demonstrated in previous studies, β-value density during hepatocarcinogenesis displayed a trend to global hypomethylation (Figure 2B and Supplemental Figure 2A). To further identify differentially methylated genomic regions (DMGRs) associated with HCC development and progression, we compared epigenetic alterations of dysplastic and cancer lesions to noninfected and noncirrhotic liver (NL) (DMGR_NL_: *n* = 10; Figure 2C). Consistent with the observed pathway activation of epigenetic modifications, we detected an increase and maximum peak of DMGRs in eHCC lesions (DMGR_eHCC_: *n* = 4965; DMGR_pHCC_: *n* = 1702) in comparison to other stages of the disease (Figure 2D). While hypomethylated marks were mainly located in open sea regions, hypermethylated marks that progressively increased during hepatocarcinogenesis occurred mainly in CpG Island regions, suggesting regulatory importance and potential impact on gene expression (Figure 2E and Supplemental Figure 2B). Unsupervised cluster analyses based on the identified DMGRs effectively subdivided normal liver from CL as well as from preneoplastic and malignant lesions. Interestingly, we did not achieve a sharp distinction between cirrhotic parts, preneoplastic, and malignant lesions by DNA methylation profiling alone (Figure 2C). However, integrative iCluster analyses of both the DNA methylome and the transcriptome resulted in 2 distinct clusters: a malignant and nonmalignant cluster. Thus, integrative analyses yielded in an improved differentiation and identification of lesions at higher risk of malignant transformation (Figure 2F, Supplemental Figure 2C, and Supplemental Table 4).

**Figure 3:**
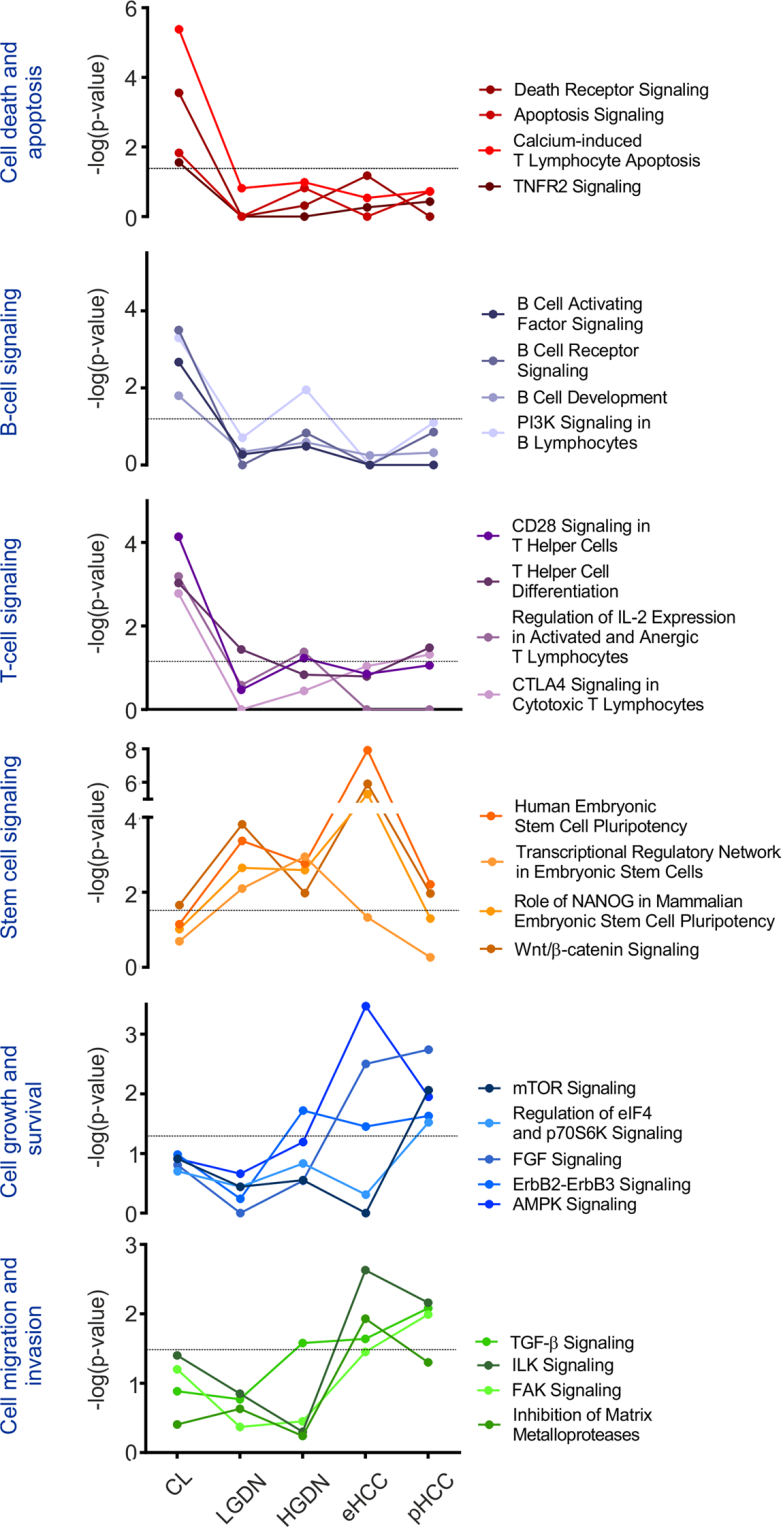
Epipathways. Signaling pathway regulation during sequential evolution of HCC analyzed by IPA based on detected stage-specific DMGR to NL. Significance of each pathway was determined by scoring system provided by the IPA tool.

We next analyzed functional networks and signaling pathway regulation of identified DMGRs. While early epigenetic alterations from CL to LGDN centered on signaling pathways related to cell death, apoptosis, and immune regulation, late changes involved cell survival, growth, and migration. We further detected a common regulation of stem cell–associated pathways including Wnt/β-catenin signaling only in DNs as well as eHCC (Figure 3 and Supplemental Table 5).

**Figure 4:**
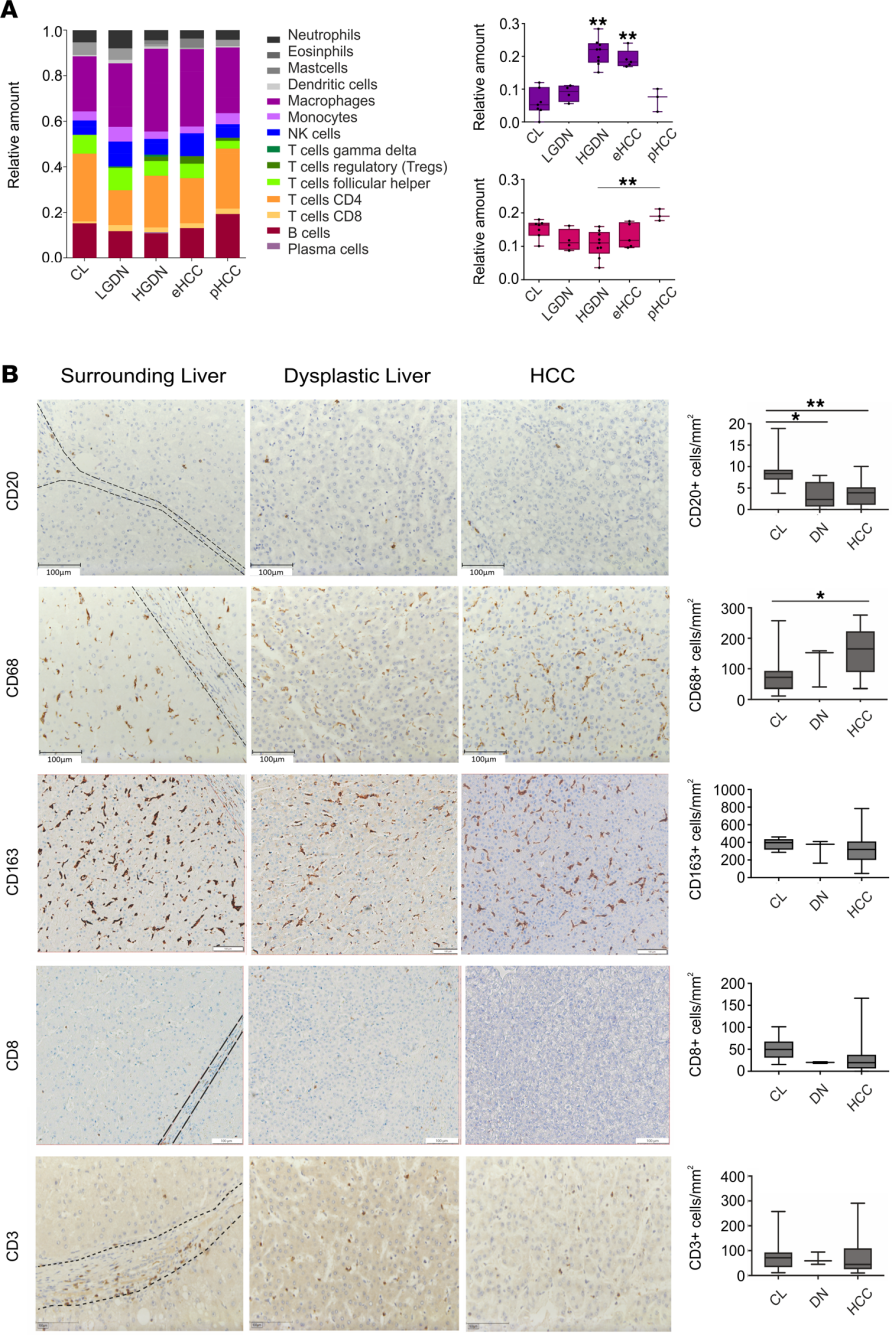
Immune regulation. (**A**) Relative amount of immune cells analyzed by Cibersort based on differentially expressed gene signatures during sequential evolution of HCC. Relative amount of (a) macrophages M0 and (b) naive B cells during sequential evolution of HCC detected by cibersorting; 1-way ANOVA (Bonferroni Correction); **P < 0.01. (**B**) IHC of representative areas of macrophages (CD68), M2 macrophages (CD163), B cells (CD20), and T cells (CD3, CD8) expressed as cell number per mm^2^ of an independent cohort of HCV-infected patients with CL (*n* = 10), DNs (*n* = 3; *n*_CD20_=5), and HCC (*n*_CD20_ = 25; *n*_CD68_= 23; *n*_CD163_= 49; *n*_CD8_= 50; *n*_CD3_= 24); Kruskal-Wallis test was used to assess statistical significance; *P <0.05; **P <0.01.

We next dissected the immune cell composition based on the gene expression profiles using the cibersort tool that revealed early changes of the immune compartment in the diseased microenvironment (Figure 4A). Interestingly, we saw an increase in M0 macrophages during the transition from LGDN to HGDN and eHCC, whereas B cell content was considerably reduced in premalignant and malignant lesions and increased in pHCC (Figure 4A). To evaluate if these early changes of the immune compartment during malignant transformation are valid and applicable independent of the underlying etiology, we explored the presence of B cells (CD20+), T cells (CD3+, CD8+), and macrophages (CD68+, CD163+) in an independent cohort of patients infected with HCV. Consistently, we were able to confirm an increase of macrophages (CD68+) during malignant transformation. Interestingly, we did not observe changes in the CD163+ macrophage (M2) population (Figure 4B). Furthermore, we consistently detected a significant decrease of B cells already in dysplastic lesions and HCC lesions, whereas the population of T cells did not significantly change (Figure 4B).

### Identification and validation of epidrivers in HCC development and progression.

**Figure 5:**
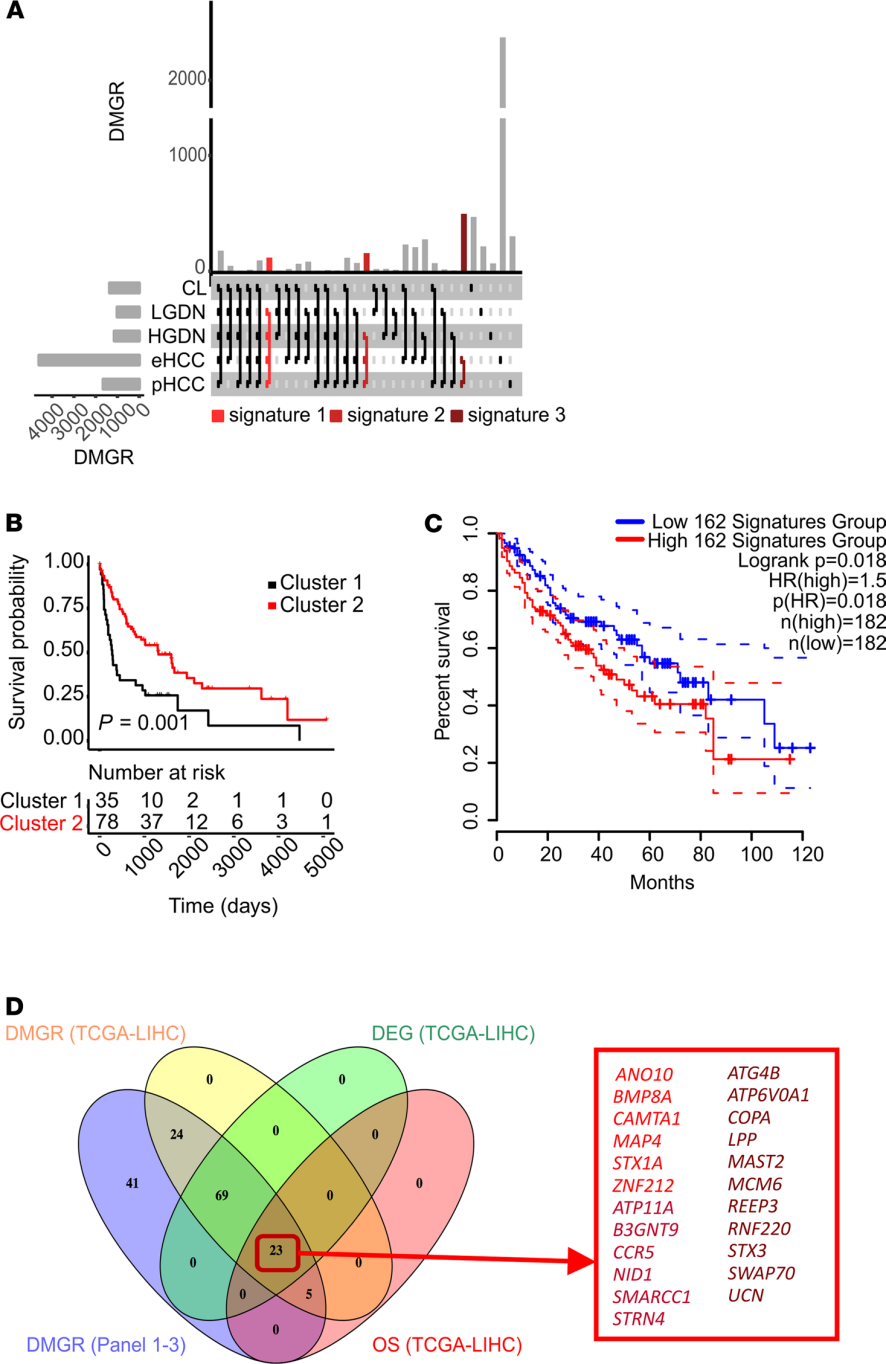
Identification and validation of DMGR in TCGA-LIHC cohort. (**A**) Upset plot showing the overlap of DMGRs to NLs across each stage of disease. (**B**) Kaplan-Meier analysis based on the specific transcriptome profile of 162 DMGRs in tumor tissue using publicly available data from authentic human HCCs of 139 patients from Lee et al. cohort (24). Survival analyses were performed by CRAN package survival and survminer (version 0.4.3) using log rank tests. *P* = 0.001. (**C**) Kaplan-Meier analysis based on the specific transcriptome profile of 162 DMGRs in tumor tissue using publicly available data from authentic humans of the TCGA-LIHC cohort using the GEPIA.2 tool. (**D**) Venn Diagram of identified DMGR_Panel1-3_ (*n* = 162) and validated DMGRs in the TCGA-LIHC cohort including DNA-methylation status (yellow, *n* = 121), gene expression status (green, *n* = 92), and association to overall survival (OS; red, *n* = 23).

To identify epigenetic alterations with high oncogenic potential, so called epidrivers, we defined 3 signatures of early DMGRs common in all lesions from LGDN to pHCC, of late DMGRs common in all lesions from HGDN to pHCC, and of malignant DMGRs common in eHCC and pHCC. Signatures of early and late epigenetic changes included 117 and 156 DMGRs, while signature 3 compromised 495 DMGRs (Figure 5A and Supplemental Table 6). Several DMGRs have been previously described in the context of HCC, including *NKX6-2, NSD1, TBX15*, and *ZIC1* (9). Next, we performed integrative analyses of our RNA-Seq data to define those DMGRs that lead to a concomitant progressive gene expression alteration in cancer tissue (eHCC and/or pHCC). We detected 24 (20.5%) DMGRs out of signature 1, 24 (15.38%) out of signature 2, and 114 (23.03%) out of signature 3 (Supplemental Figure 3A and Supplemental Table 7) with gene expression changes in HCC lesions. Again, our analyses identified several previously described DMGRs with expression changes during cancer development and progression including *GLTSCR1, THRSP,* and *ATP6V1C1* (7). Analyses of deregulated networks associated with the 162 identified DMGRs with expression changes in HCC centered on connective tissue development and function, cellular development, growth and proliferation, embryonic development, and cancer. Cellular functions showed activation in cellular movements, cell morphology, and signaling (Supplemental Table 8). We further confirmed a significant enrichment in signaling pathways associated with stem cell activation, immune regulation, and oncogenic traits such as cell growth, survival, and migration/invasion (Supplemental Figure 3B). To next investigate whether the 162 identified DMGRs have potential impact on biological traits of tumors, we integrated our results with an independent cohort of patients with HCC (24) and the cohort of advanced HCC from the TCGA-database (TCGA-LIHC cohort, *n* = 366 HCC) and assessed clinical outcomes by subclustering the tumors based on the expression profiles of the 162 DMGRs.

Notably, significant association to overall survival of patients could be revealed in our independent patient cohort as well as in the TCGA-LIHC cohort (Figure 5, B and C) (25). The subanalyses of each panel confirmed a significant association to the outcome of Panel 1 and 2 in the TCGA-LIHC cohort and of Panel 3 in the HCC cohort of Lee et al. (Supplemental Figure 4) (24).

Next, we validated methylation and expression changes of each gene of the 162 gene signatures as well as their prognostic association and relevance for cancer progression using the TCGA database. We compared differentially methylated probes (DMPs) in cancer tissues to NLs (*n* = 75) and validated differential DNA methylation in 121 genes out of our 162 identified epigenetic oncogenic marks (Figure 5C and Supplemental Table 9). Among the validated 121 DMGRs, we further confirmed expression changes in 92 genes, of which expression of 23 genes had a significant impact on overall survival of patients with HCC (Figure 5D and Supplemental Tables 10 and 11). Multivariate analyses revealed 14 out of 23 genes with significant prognostic implications in HCC (Supplemental Table 12).

Functional network analyses of these epidrivers confirmed relevance of the genes for cancer, organismal injury and abnormalities, gene expression, and cell cycle as well as connective tissue development and function (Supplemental Figure 5A). Signaling pathway analyses of the putative epidrivers further centered on molecular mechanisms of cancer, cell survival, proliferation, and invasion as well as stem cell activation and immune regulation (Supplemental Figure 5B). While some genes (*ATG4B, CCR5, MCM6, UCN*) were detected in the context hepatocarcinogenesis before, most of the epidrivers were the result of the investigation of our unique cohort (26–29).

### STRN4 is a potentially novel oncogenic epidriver in HCC progression.

**Figure 6:**
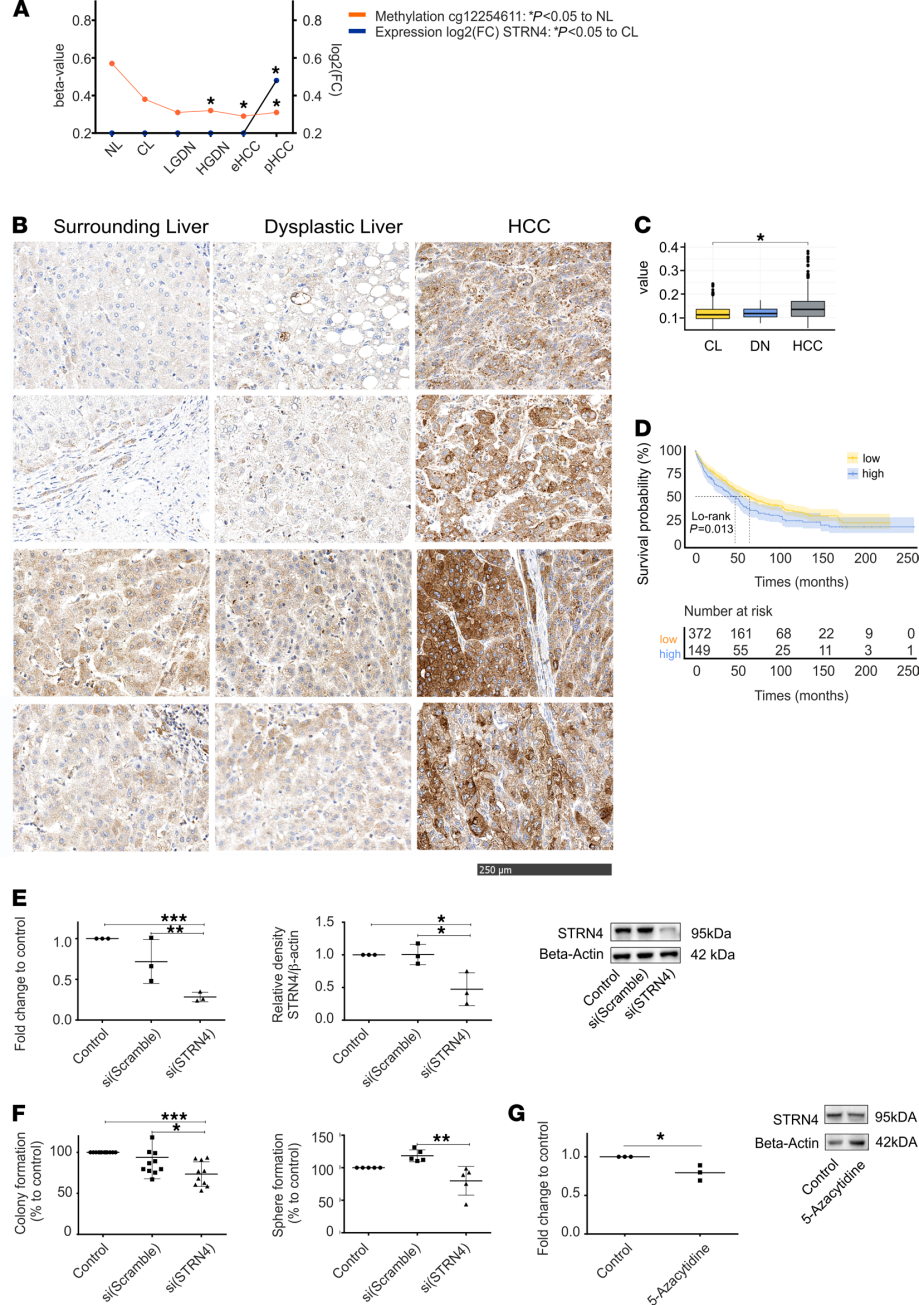
STRN4 as a potentially novel epigenetically regulated oncogene. (**A**) Methylation and expression of STRN4 across all stages of hepatocarcinogenesis. Differential methylation was determined as FDR-corrected *P* < 0.05 and/or a minimum β-value difference ≥ ± 0.2. Differential expression: Significance testing was performed using Wald test statistics. *P* < 0.05. (**B**) Representative images of expression of STRN4 in tissue microarrays analyzed in 521 patients with HCC to dysplastic nodules (*n* = 10) and surrounding liver tissue (*n* = 526). (**C**) Cytoplasmatic optical density analyses of tissue microarrays expressed of STRN4 in HCC (*n* = 524) to DN (*n* = 10) and CL (*n* = 526). Kruskal-Wallis test was used to assess statistical significance; *P < 0.001. (**D**) Impact on overall survival of STRN4 for 521 patients with HCC using log rank tests. (**E**) Expression of STRN4 in Huh7 cell lines in nontreated (control), si(Scramble), and siSTRN4-treated cells on mRNA (left) and protein level (right). (**F**) Colony formation (left) and sphere formation (right) of Huh7 cell lines in nontreated (control), si(Scramble), and siSTRN4-treated cells; 1-way ANOVA: *P < 0.05; **P <0.01; ***P < 0.001. (**G**) Expression of STRN4 in Huh7 cell lines in nontreated (control) and 5-Azacytidine treated cells on protein level; Student’s *t* test: *P <0.05.

The application of integrative genomic analyses enabled us to define putative epigenetic driver alterations with relevance to malignant transformation and progression in the liver. Next, we evaluated if a newly identified molecule could be a new target for potentially novel therapeutic approaches as a proof of concept. Among the 23 putative epidrivers associated with overall survival, we identified STRN4 as a putative pro-oncogenic molecule specifically activated in late stages of hepatocarcinogenesis. Our DNA methylation analyses revealed an early, progressive hypomethylation of the body region of *STRN4* (cg12254611, Chromosome 19: 47,249,193). Consistent with a stepwise activation, activation of gene expression occurred late and was dependent on the degree of hypomethylation (Figure 6A). Using publicly available databases, we confirmed upregulated expression of *STRN4* in several tumor types, including HCC (Supplemental Figure 6A). Importantly, high expression of *STRN4* was significantly associated with poor prognosis of patients with HCC (Supplemental Figure 6B). Upregulation of STRN4 in HCC in comparison to corresponding nontumor tissue as well as DNs was further validated by IHC using an independent cohort of 521 patients of the University Medical Center Mainz with confirmed HCC involving different etiologies (Figure 6, B and C). Clinicopathological characteristics of the patients are displayed in Supplemental Table 13. Patients with HCC with high expression of STRN4 had a significantly worse outcome compared with patients with HCC with low expression (Figure 6D). Next, we functionally explored the tumorigenic potential of STRN4. We silenced STRN4 expression in hepatoma cell lines Huh7 (Figure 6E) and Hep3B (Supplemental Figure 7, A and B) by siRNA. Consistently, decreased STRN4 expression resulted in an impaired ability to form colonies and spheres, implicating impairment of their oncogenic potential (Huh7: Figure 6F; Hep3B: Supplemental Figure 7C). Finally, to confirm the epigenetic regulation of STRN4, we employed an epigenetic unmasking approach using treatment of cells with 5-AZA. We observed a significant downregulation of STRN4 by 5-AZA treatment (Figure 6G). These investigations establish the importance of STRN4 as a potentially novel oncogenic epidriver in HCC.

## Discussion

Oncogenesis in the liver involves a multistage process that is fueled by chronic inflammatory liver diseases (3). The early dysplastic lesions emerge in the disrupted tissue microenvironment and subsequently progress to early and advanced HCC lesions (16).

Here we addressed clonal evolution of individual lesions as well as intertumoral heterogeneity in HCC and further defined a detailed catalogue of epigenetic alterations that promote human hepatocarcinogenesis.

Our unique cohort of HBV-infected patients included the complete spectrum of early and advanced stages synchronously arisen in the same patient. To date, few data are available that address whether multifocal HCC result by an intrahepatic metastatic process or by multicentric carcinogenesis. We here investigated the evolutionary background of dysplastic and cancerous lesions synchronously detected in the same patient by mtDNA profiling and addressed the degree of corresponding intertumoral heterogeneity (19). Heteroplasmic mt-variants were highly heterogeneous across preneoplastic and cancer lesions (Figure 1, C and D). Therefore, our results indicate that multifocal coexisting preneoplastic and cancerous lesions might not regularly derive from the same clonal origin within individual livers but rather emerge as de novo clones and, potentially, as a consequence of the ubiquitous inflammatory cell death. These observations are consistent with previous findings, which employed multiomic approaches and revealed a profound intra- and intertumoral heterogeneity in HCC indicative of multiclonal origins in multifocal HCCs (17, 30, 31). A limitation of this study is the limited sample size of the patient cohort. However, our investigations on mtDNA profiling for intertumoral heterogeneity represent relevant findings in this rare and unique cohort of patients with different stages of HCC disease in the same liver and warrant further investigations in larger collectives. Interestingly, we observed that the number of variants per sample was similar across all stages (~30%) of disease, reflecting high mutation rates already in the diseased cirrhotic livers (Figure 1A). Consistently, we have detected cellular alterations within the diseased livers, including large liver cell changes (LLCCs) (87.5% grade ≥ 2) and small liver cell changes (SLCCs) (62.5% grade ≥ 2; Supplemental Table 14), representing preneoplastic dysplastic lesions less than 1 mm in diameter without a circumscribed nodular appearance. Detection and extent of LLCCs and SLCCs have been related to hepatocarcinogenesis in several studies (32, 33). These results confirm that severe preneoplastic changes in the diseased microenvironment potentially predispose malignant transformation even before defined lesions emerge. Recent investigations suggest that the pronounced hepatic field effect might be induced by methylation abnormalities in early preneoplastic phases of HCC that precede genomic instability in advanced stages (17). Consistently, we detected late acquisition of transcriptomic changes in key oncogenic pathways, whereas pathways related to ‘DNA methylation and transcriptional repression signaling’ were predominantly operative in DNs with a peak of differentially methylated genes in eHCC lesions. These results underline early acquisition of epigenetic alterations in liver cancer, whereas other molecular alterations, i.e., transcriptomic and genetic changes, seem to dominate disease progression in late stages of the disease. While we observed a trend toward global hypomethylation during hepatocarcinogenesis (Figure 2B), our results on DMGR during sequential evolution of liver cancer revealed a stepwise increase in methylation events with he highest frequency of changes in early HCC (Figure 2, D and E). These findings are in concordance with recent studies and findings in patients with HBV describing an increase in hypermethylated genes during hepatocarcinogenesis (16). Subsequent whole methylome analyses revealed that DMGRs of cirrhotic liver, preneoplastic and cancerous lesions are highly divergent from NLs. Interestingly, we detected stem cell–associated pathways including Wnt/β-catenin signaling activated already in dysplastic as well as eHCC, potentially predisposing tumor development. These investigations are in agreement with a potential epigenetic progenitor cell origin in HCC (6). Our results further confirm the dominant role of WNT as a driver of hepatocarcinogenesis especially in HBV-driven HCCs (34). Consistent with previous findings, late changes centered on key oncogenic pathways of cell survival, growth, and metastasis (5). Furthermore, integrative iCluster analyses of both the epigenome and the transcriptome effectively separated malignant from nonmalignant clusters overall, confirming that early acquisition of DNA methylation contributes to gene expression changes during sequential evolution of HCC. Multilevel integrative analyses therefore provide a powerful tool to effectively differentiate and identify lesions at higher risk for a malignant transformation.

Computational immune phenotyping of HCC recently showed that the immune composition varied largely across samples and highlights severe transformation of the immune microenvironment from activating anti-tumor effector cells to resting suppressive immune cells (35). Our results demonstrate that early changes involved apoptosis and immune regulation pathways that emerge from differential regulation in cirrhosis and DNs, confirming early acquisition of immune escape mechanisms during hepatocarcinogenesis. Consistently, deconvolution of the immune cell composition by cibersorting confirmed differential immune cell infiltration at early stages. Based on our molecular data, we observed an increase in macrophages during transition from LGDNs to HGDNs and eHCC, whereas B cell content tends to be reduced in premalignant and malignant lesions and slightly increased in pHCC. Although no clear trend could be revealed, pHCC sample size was particularly limited in our cohort and might be underrepresented in the cibersorting analyses. Thus, we performed IHC staining using an independent cohort of patients with HCC to explore and validate changes in the immune composition in a larger patient cohort. These analyses confirmed a significant early decrease of B cells and a progressively increase of macrophages during hepatocarcinogenesis by IHC analyses independent of the underlying etiology. The obtained findings are in concordance with previous findings that underline the importance of macrophages for hepatocarcinogenesis (36). Furthermore, a recent paper demonstrated that densities of T and B cells are associated with the survival of patients with HCC (37). In light of the changes of immune regulation in early stages of hepatocarcinogenesis observed in our study, application of immune therapeutic approaches might be justified, particularly in potentially novel adjuvant treatment approaches currently under clinical evaluation.

In contrast to previous studies that focused on single genes, stage-specific, or on late stages of hepatocarcinogenesis (4, 16, 38, 39), we here systematically examined epigenetic changes that commonly occurred across the different stages of the disease and controlled gene expression, i.e., bona fide epidrivers. As expected, early changes commonly disrupted showed the lowest amount of DMGRs whereas DMGRs associated with eHCC and pHCC involved almost 500 alterations. Integration with our transcriptomic data set revealed a total of 162 differentially methylated genes with concomitant expression changes in oncogenic lesions. We further confirmed that the gene expression signature of the 162 DMGRs showed significant association to overall survival in 2 independent HCC cohorts (Figure 5, B and C) (24) Moreover, we validated a total of 121 (75%) DMGRs in the TCGA-LIHC data set with concomitant expression changes in 92 DMGRs (57%; Figure 5D) out of our 162 identified DMGRs, proving, therefore, that validated DMGRs were independent from underlying HCC etiology. Among them, we confirmed that gene expression changes in 23 genes were significantly associated with survival of patients with HCC. Among those, more than 60% (14/23) of the genes showed an independent impact on overall survival in the TCGA-LIHC cohort as revealed by multivariate analyses (Figure 5D and Supplemental Tables 11 and 12). Importantly, only a few genes were previously implicated with liver cancer development *(ATG4B, CCR5, MCM6, and UCN*) (26–29), whereas most of the epidrivers were newly identified.

In line with recent findings that suggest robust prognostic impact of DNA methylation-driven genes (40), our integrative epigenetic analyses provide a powerful approach to identify potentially novel drivers of hepatocarcinogenesis.

Among newly identified epidrivers, we identified STRN4 activated in late stages of hepatocarcinogenesis. STRN4 belongs to the striatin protein family, which is part of the striatin interaction phosphatases and kinases complex (41). Recent studies have revealed metastatic and protumorigenic properties of STRN4 in several tum or etiologies including colorectal and prostate cancer as well as non–small cell lung cancer (NSCLC) (42–45). Indeed, we detected upregulated gene expression of *STRN4* in several tumor types, including HCC in the TCGA database. We further confirmed upregulated protein expression by IHC using an independent cohort of 521 patients with HCC (Figure 6, B and C). Importantly, validations of the findings in independent cohorts compromising 887 patients with HCC confirmed that high STRN4 expression was significantly associated with poor prognosis (Figure 6D and Supplemental Figure 6B). Thus, our study potentially provides the first evidence that STRN4 indeed possesses oncogenic properties in liver cancer. Consistently, silencing of STRN4 significantly affected tumorigenic properties of human hepatoma cells (Figure 6, E and F) and might provide a new promising target for therapeutic applications in HCC.

In conclusion, we analyzed the epigenetic landscape during sequential evolution of hepatocarcinogenesis. The study provides evidence that early epigenetic alterations promote immune escape and induces stemness properties in preneoplastic lesions, thus, enhancing malignant properties during liver cancer development and progression. We subsequently defined and validated new epigenetic driver alterations including STRN4 that might provide new predictive and therapeutic opportunities for patients with HCC.

## Methods

### Samples.

A total of 28 samples were collected, including 7 surrounding liver tissues, i.e., CL, 4 LGDNs, 9 HGDNs, 5 eHCCs, and 3 pHCCs from 8 patients with HCC with chronic HBV infection. Nodules were resected from explanted cirrhotic livers. All lesions were classified according to the criteria of ‘International Consensus Group for Hepatocellular Neoplasia’ by 2 independent expert pathologists (46). All procedures were approved by the local authorities and prior patient consent was obtained. Demographic and clinicopathological data of the patients can be found in Table 1.

### Nucleic acid extraction.

Total RNA was extracted using the Qiagen RNeasy Mini Kit following the manufacturer’s instructions. RNA quantity and purity were estimated using a Nanodrop ND-1000 Spectrophotometer, and integrity was assessed by an Agilent 2100 Bioanalyzer. DNA was extracted using a Qiagen Qiamp DNA Kit following the manufacturer’s instructions.

### Whole mtDNA ultra-deep sequencing.

Multiplex PCR-based ultra-deep sequencing analysis of the whole mt-genome was performed and analyzed as previously described (19). In brief, PCR-amplicable DNA was quantified by real-time PCR using the HFE gene as amplifying reference (173 bp). Standard curves in a range of 0.195–50 ng were prepared from unmutated high quality DNA (Takara Bio Europe). Real-time PCR was then carried out in triplicate with 1 μL DNA each, in a 20 μL reaction mix containing 0.4 μM of the HFE forward and reverse primer (HFE-173F: TTCTCAGCTCCTGGCTCTCATC and HFE-173R: TCGAACCTAAAGACGTATTGCCC) and the GoTaq qPCR Master Mix (Promega). To generate amplicons of a low size (around 60–200 bp), 108 primer sets spanning the whole mtDNA were designed according to the mt-sequence of accession no. NC_012920 or taken from previously published primer sets. For enrichment of the mt-genome by a multiplex PCR, primer sets were pooled in 4 primer mixes of 2 μM and in each reaction, 10 ng of PCR-accessible DNA representing DNA of approximately 1500 cells were used. mtDNA was then amplified in 4 separate multiplex PCR reactions per sample using the GeneRead DNAseq Panel PCR Kit (QIAGEN) in accordance with the manufacturer’s protocol. Libraries were pooled and purified using Agencourt AMPure XP magnetic beads and a Biomek FXp workstation (Beckman Coulter). Fifty nanograms of enriched targets of each sample were adenylated and ligated to NEXTflex DNA barcodes-48 (Bioo Scientific). Agencourt AMPure XP magnetic bead purification and size selection, barcoded libraries were amplified by PCR cycles. Finally, 12 pM of the constructed libraries were sequenced using the V2 chemistry of Illumina and 2 × 300 bp sequencing read length on an Illumina MiSeq platform following the manufacturer’s recommendations. The FASTQ files generated by the Illumina platform were analyzed by means of the Biomedical Genomics Workbench 2.5.1 (QIAGEN, www.qiagenbioinformatics.com). To determine run performance and o-target reads, the FASTQ sequences were mapped against the whole human reference genome hg19. For variant calling and annotation, the mt-genome (Genebank, accession NC_012920) served as a reference. Using the workflow tool of the Biomedical Genomics Workbench 2.5.1 software in batch mode ensured successive and identical analysis of all samples. The minimum read depth was set to 30, with the minimum variant frequency set to 5%. Furthermore, variant calling was restricted to loci with a balanced forward-backward performance (> 0.2). Polymorphisms were recognized using the MITOMAP (http:// www.mitomap.org/bin/view.pl/MITO-MAP/HumanMitoSeq), dbSNP-v138), (http://www.ncbi.nlm.nih.gov/ SNP/_id=138), and HAPMAP_phase_3 http://hapmap.ncbi.nlm.nih.gov/hapmap3r3_B36/) databases. Spurious calls were subsequently filtered by manual analysis. Variants, which occur in different sample sets but with a similar frequency as well as variants that were located in repetitive or highly homologous regions of the mt-genome, in high background noise regions, or at the end of the amplicons were considered as putative false variants. Potential false positive variants were either deleted when they were clearly recognizable as artifacts or were further reassessed by Sanger sequencing. In addition, whenever DNA was still available, the mtDNA regions carrying a variant in 1 lesion sample but not in another of the same patient sample set were subsequently reanalyzed by conventional Sanger sequencing (Supplemental Table 15 and Supplemental Figure 1B). The frequency of variants represents the number of variants normalized to sample size (19).

### Methylome-Seq.

Methylome profiling was performed using Infinium Human Methylation 450k BeadChips analyses and deposited at the Bioproject database (https://www.ncbi.nlm.nih.gov/geo/query/acc.cgi?acc=GSE146286). DMGRs were identified in comparison to noninfected NL (*n* = 10) samples that were provided by Jesper B. Andersen (BRIC, Department of Health and Medical Sciences, University of Copenhagen) and defined with a minimum β-value difference of greater than or equal to 0.2 and a (maximal) FDR of 0.05 using the R package nlme (version 3.1-141). The patient id was included as random effect. Cluster analysis was performed using the stats R package (version 3.1-141)using Euclidean distance.

Functional classification and network analysis were performed using Ingenuity Pathway Analysis (Qiagen). Significantly differentially methylated genes of each stage of the disease were uploaded and a comparison analysis based on log ratio was performed. The analysis determines the most significantly affected pathways displayed by a –log (*P* value). *P* values of less than or equal to 0.05 were considered statistically significant. Upset plots were generated using the Package UpSetR 1.4.0 tool.

### RNA-Seq.

RNA-Seq was performed using Illumina HiSeq2000 and Illumina HiSeq4000. Raw reads were filtered by removing adapter sequences, contamination, and low-quality reads. The reads were then mapped with human genome reference sequence (GRCh37.82) using HISAT2 (hisat2-2.0.2-β) followed by read summarization with feature counts (subread-1.5.0-p1) (47–49). All data analysis was performed using R programming language and related packages. The output matrix from feature counts was input into the Bioconductor package DESeq2 for differential expression analysis (50). Significance testing was performed using Wald Test statistics. The IPA online tool provided by Qiagen was used for functional classification and pathways analyses. Significantly differentially expressed genes of each stage of disease were uploaded and a comparison analysis based on log ratio was performed. The analysis determines the most significantly affected pathways displayed by a –log (*P* value). *P* values of less than or equal to 0.05 were considered statistically significant. The significance of each network, function, and pathway was determined by the scoring system provided by the IPA tool.

### Integrative analyses.

Venn Diagrams for integration of methylome and transcriptome data were generated using the VENNY software by (bioinfogp.cnb.csic.es/tools/venny/index.html).

Cluster analysis of methylome and transcriptome data was performed using the R package iCluster (version 2.1.0). The top 150 differentially expressed genes and the top 150 differentially methylated genes from each pairwise comparison were intersected and the top 100 genes and the top 100 CpGs were selected by *P* values as input for iCluster (Supplemental Table 4). Estimation of the immune cell composition based on gene expression data was performed using the Cibersort-Tool (51).

### Validation of DNA methylation analysis using TCGA-LIHC consortium and publicly available databases.

Infinium Human Methylation 450k BeadChip data were analyzed for the following sample types: normal liver (*n* = 8; GSE69852; ref. 52; GSE48472; ref. 53), liver from obese patients (*n* = 67; GSE61446; ref. 54), liver tumors from TCGA-LIHC consortium (*n* = 366; NCI Genomic Data Commons) (35). Raw IDAT files were analyzed using RnBeads package (v3.8) (55), including extensive probe filters (exclusion of poor-quality probes, sex chromosome probes, and probes containing more than 2 common SNPs in their genomic mapping region) followed by BMIQ normalization. Methylation was quantified using the β-value metric, ranging from 0 (0% methylation) to 1 (100% methylation). DMPs were defined as those with an FDR of less than or equal to 0.05 when compared between 2 groups and/or a minimum β-value difference greater than or equal to 0.2. Differential expression between tumor and adjacent normal tissues for selected DMGR of Panels 1–3 has been investigated using the DiffExp module of TIMER-Tool (https://cistrome.shinyapps.io/timer/). The impact on overall survival has been analyzed by survival analyses with a group cutoff at the median using the GEPIA2-Tool (http://gepia.cancer-pku.cn/index.html). Significance of genes for outcome was further evaluated by the Cox proportional hazard model using the Gene_Outcome and Gene_Surv module of the TIMER2.0 tool (http://timer.cistrome.org) including clinical factors (i.e., age, gender, race, and stage of disease) of the TCGA-LIHC cohort.

### IHC of tissue microarrays.

A tissue microarray containing samples from 571 patients with HCC who underwent tumor resection at the University Medical Center Mainz from 1997–2018 was established. The human tissue samples were provided by the Tissue Bank of the University Medical Center Mainz after approval by the local ethics committee (Ethik-Kommission der Landesärztekammer Rheinland-Pfalz, 837.146.17, 10980, as well as addendum 2018-13857_1 to DAR and BKS). After heat-induced antigen retrieval tissue, microarray slides were stained with a mouse anti-STRN4 antibody (Abnova, MAB12008, dilution 1:2500; Supplemental Table 16). Staining was performed using an automated staining system (DAKO Autostainer plus, Agilent Technologies) and the Dako EnVision FLEX staining system (Agilent Technologies) in accordance with the manufacturer’s instructions. Prior to image analysis, TMA slides were digitalized using the NanoZoomer-Series Digital slide scanner (Hamamatsu Photonics). Digital image analysis was performed using the HALO platform from Indica Labs including the TMA module and the CytoNuclear v1.6 module. Missing or erroneous cores, e.g., with extensive tumor necrosis, were excluded from the analysis. Cytoplasmic optical density was determined as the target parameter and was correlated with clinical data.

### IHC of immune cells.

From a total of 53 patients with HCC with chronic HCV infection who underwent tumor resection at the University Medical Center of Cologne, different areas with cirrhosis, DNs, and hepatocellular carcinoma were studied by IHC. All immunostainings were conducted using the BOND MAX automated staining system (Leica Biosystems). Macrophages were stained with CD68 antibodies (Agilent Technologies, dilution 1:400) and tumor-associated macrophages with CD163 (Cell Marque, dilution 1:100). For IHC of B cells, CD20 antibodies (Agilent Technologies, dilution 1:1250) were used. For IHC of the T cell populations, a CD3 antibody (Thermo Fisher Scientific, dilution 1:50) and a CD8 antibody (Agilent Technologies, dilution 1:200) were used. Images were acquired using the Olympus DP74 camera system and the cellSens standard software (Olympus). Immunostained cells were counted in representative areas between 0.1–0.7 mm^2^ by means of the Image J (NIH) cell counter plugin tool (https://biii.eu/cell-counter-imagej) and expressed as cell numbers per mm^2^. A Kruskal-Wallis test was used to assess statistical significance.

### Cell lines, siRNA, and 5-Azacytidine treatment.

Human hepatoma cell lines Hep3B and Huh7 were cultured in DMEM, supplemented with 2 mM L-glutamine, 1 unit/mL penicillin/streptomycin, and 10% FCS at 37°C and 5% CO_2_ as recommended (56). Huh7 were obtained from the cell lines service (RIKEN) and Hep3B from the global bioresource center (ATCC). Cells were treated for 6 hours with silencing RNA against STRN4 at the concentration of 60 nM. Three siRNAs were tested (Silencer Select Predesigned siRNA from Ambion, 1. ID: 134123; 2. ID: 134124; 3. 134125) and showed effective downregulation of STRN4 (Supplemental Figure 7A). siSTRN4-3 has been used for further experiments. After 6 hours of incubation, siSTRN4-3 has been removed and cells were incubated for an additional 72 hours before functional analyses. For 5-Azacytidine treatment, Huh7 cells were treated for 72 hours with 5-Azacytidine (Selleckchem, S1782) with IC50 concentration (200 μM) before expression analyses by Western blotting. All experiments were performed in 3 independent replicates.

### Real-time PCR.

A 2-step real-time qPCR, cDNA synthesis using Superscript III (Invitrogen), SYBR Green MasterMix (Bio-Rad), and *iQ5 or CFX Connect* System was performed. Oligonucleotide primers were designed using Primer3 v.0.4.0 (http://frodo.wi.mit.edu/primer3/) as described previously (27). The amplification protocol was as follows: 95°C for 3 minutes, followed by 40 cycles of 95°C for 15 seconds and 1 minute at 60°C, completed by a dissociation curve to identify false positive amplicons. GAPDH was used as a reference. The relative expression level of each gene was normalized to untreated cells and calculated using the formula 2^(−ΔΔCt)^. All experiments were performed in 3 independent replicates.

### Western blotting.

Monolayer cultures of each cell line were exposed to siRNA as described. Cell lysates were prepared from frozen cells using M-PER Tissue extraction Buffer (Pierce) containing complete protease inhibitor cocktail (Roche). Protein concentrations were determined by the BCA protein assay (Thermo Fisher Scientific) following the manufacturer’s protocol. In total, 25 μg were used for Western blotting, separated by SDS-PAGE and transferred onto a nitrocellulose membrane (Hartenstein) as described previously (57). PageRuler Prestained Protein Ladder (Thermo Fisher Scientific) was used on the left site. Membranes were probed with the indicated antibodies. Antibodies were diluted 1:1000 for β-actin clone B43R (Biovision, mouse, 3598R-100) and 1:500 for STRN4 using rabbit polyclonal antibodies (Sigma-Aldrich, HPA043051). Quantification of expression levels was performed by densitometric analyses using ImageJ on original scanned membranes. All experiments were performed in 3 independent replicates.

### Colony and sphere formation assays.

Cells were treated with siRNA as described. After treatment, we seeded 1 × 10^3^ cells per plated on 6-well plates for colony formation assay and 1 × 10^3^ cells on 48-well plates for sphere formation assay on agarose at 2%. Colonies and spheres were calculated at day 14 and represented as a percentage of colonies/spheres of control. Hep3B cells did not form spheres. All experiments were performed in 3 independent replicates.

### Availability of data and materials.

The data set supporting the conclusions of this article is available in the Bioproject database (https://www.ncbi.nlm.nih.gov/geo/query/acc.cgi?acc=GSE146286).

### Statistics.

Statistical analysis was performed using 2-tailed Student’s *t* test or Mann-Whitney *U* test and for multiple group comparisons 1-way ANOVA (Bonferroni Correction) as indicated. *P* values ≤ 0.05 were considered statistically significant. Results are presented as means ± SD.

For integration of patients, publicly available expression data sets were used (24). Hierarchical cluster analyses were performed using Euclidean distance by the Bioconductor package multiClust (version 1.4.0). Missing values were computed by k-Nearest Neighbor imputation with CRAN package VIM (version 4.7.0) (58). Survival analyses were performed by CRAN package survival and survminer (version 0.4.3) using log rank tests.

STRN4 protein expression was dichotomized utilizing the Charité Cut‑off finder functions to provide a significant distinction between the high and low STRN4 protein expression levels based on survival outcome (59).

### Study approval.

All analyses were approved by the local authorities and prior patient consent was obtained. Human tissue samples were provided by the Tissue Bank of the University Medical Center Mainz after approval by the local ethics committee (Ethik-Kommission der Landesärztekammer Rheinland-Pfalz, 837.146.17, 10980, as well as addendum 2018-13857_1 to DAR and BKS).

## Author contributions

The following authors designed the experiments: CC, JUM, AP, CJO, DB, SST, MO, DAR, BKS, HB, YNP, and PRG. CC, AP, JUM, DC, BPA, DB, SHH, WA, CJO, DAR, MS, and UD performed the experiments. CC, AP, DB, BPA, MO, JBA, CJO, HB, MS, DAR, BKS, PRG, SST, YNP, and JUM analyzed the data. The paper was written by CC and JUM. All authors discussed the results and critically commented on the manuscript. All authors had access to the study data and reviewed and approved the final manuscript.

## Supplementary Material

Supplemental Figures and Tables

Supplemental table 15

Supplemental table 16

## Figures and Tables

**Table 1:**
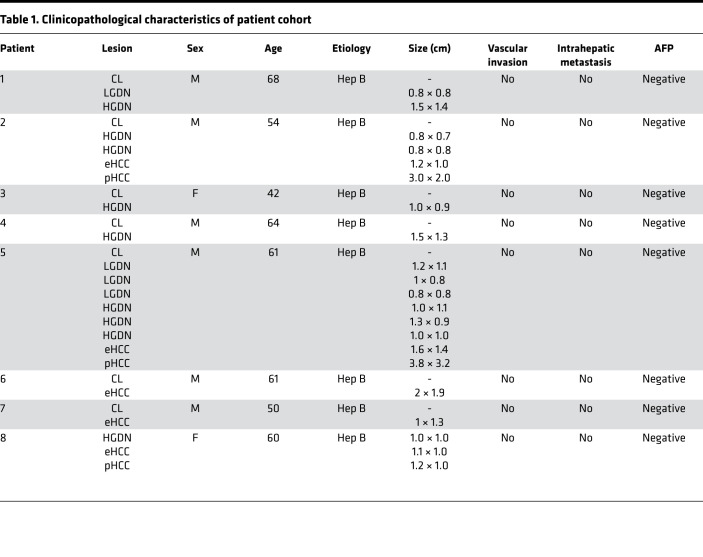
Clinicopathological characteristics of patient cohort

## Version 1 08/10/2021

In-Press Preview

## Version 2 09/08/2021

Electronic publication
