# Supplementary material for: Epigenetic modifications precede molecular alterations and drive human hepatocarcinogenesis
Source: JCI Insight. 2021 Sep 8;6(17):e146196. doi: 10.1172/jci.insight.146196 (PMC8492348; doi:10.1172/jci.insight.146196)
Supplement: Supplemental Figures and Tables [file jciinsight-6-146196-s159.pdf]

Supplemental Figure 1

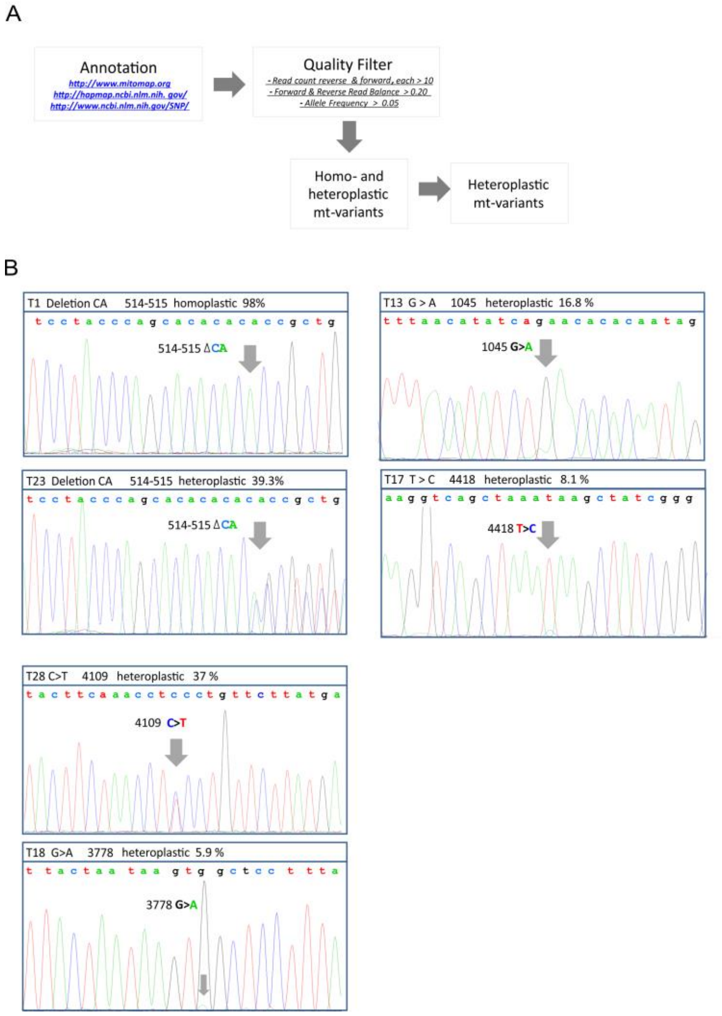

Supplemental Figure 1

A) Workflow of mitochondrial sequencing data interpretation and mt-variant frequencies.

B) Representative images of Sanger sequencing.

Supplemental Figure 2

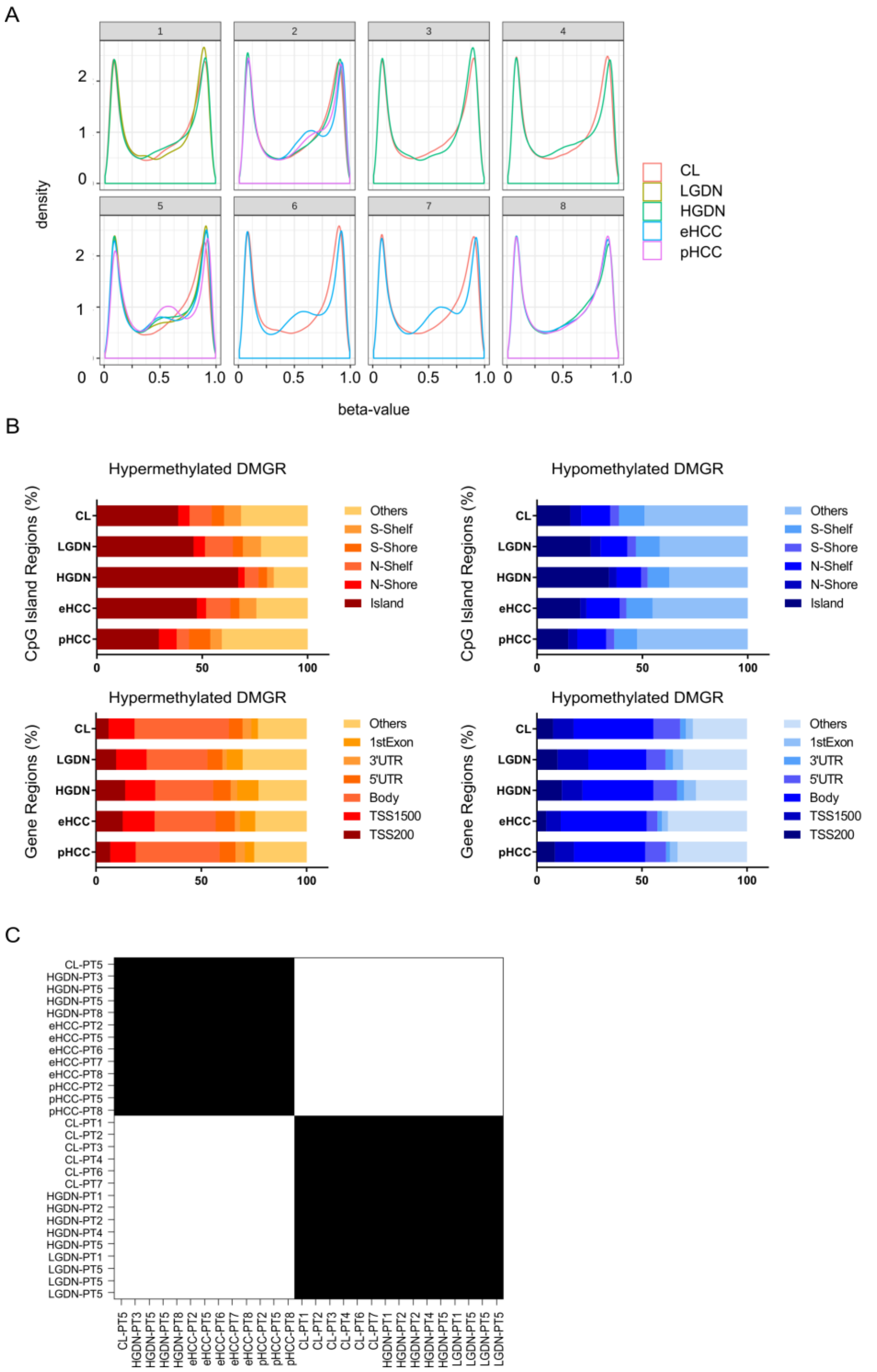

Supplemental Figure 2

A) Methylation variance in cirrhotic liver (CL), preneoplastic lesions (LGDN, HGDN) and cancerous lesions (eHCC, pHCC) of each patient (1-8) demonstrated by the beta-value density. B) Localization of hyper- and hypomethylated DMGR in CpG island regions and gene regions expressed as percentage. C) Cluster separability plots defined by iCluster analyses.

Supplemental Figure 3

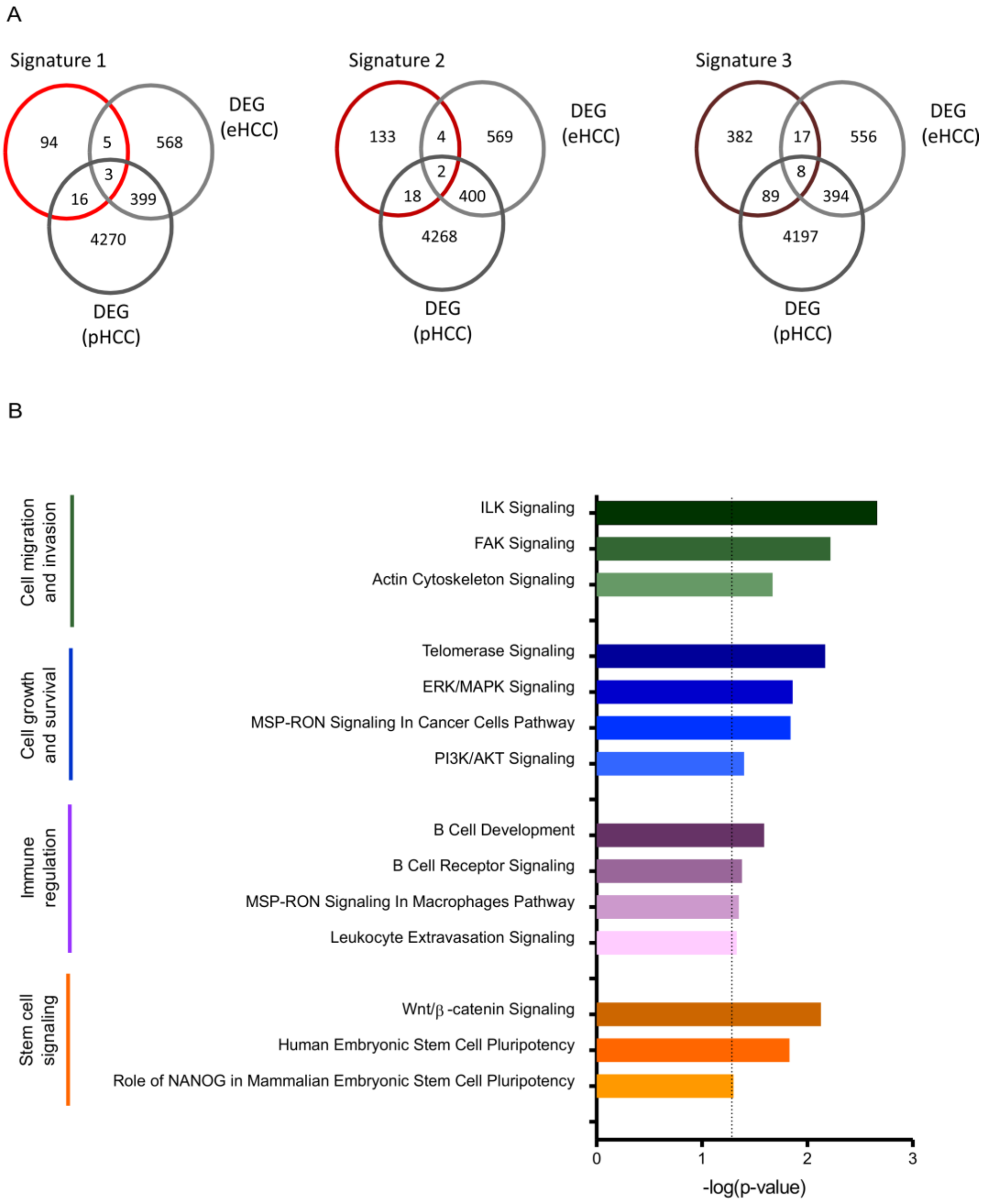

Supplemental Figure 3

A) Venn diagrams of Signature 1-3 and differentially expressed genes (DEG) of eHCC and pHCC. B) Signaling pathway regulation during sequential evolution of HCC analyzed by ingenuity pathway analyses based 162 identified genes by the overlap of DMGR and DEG. Significance of each pathway was determined by scoring system provided by Ingenuity Pathway Analysis tool.

Supplemental Figure 4

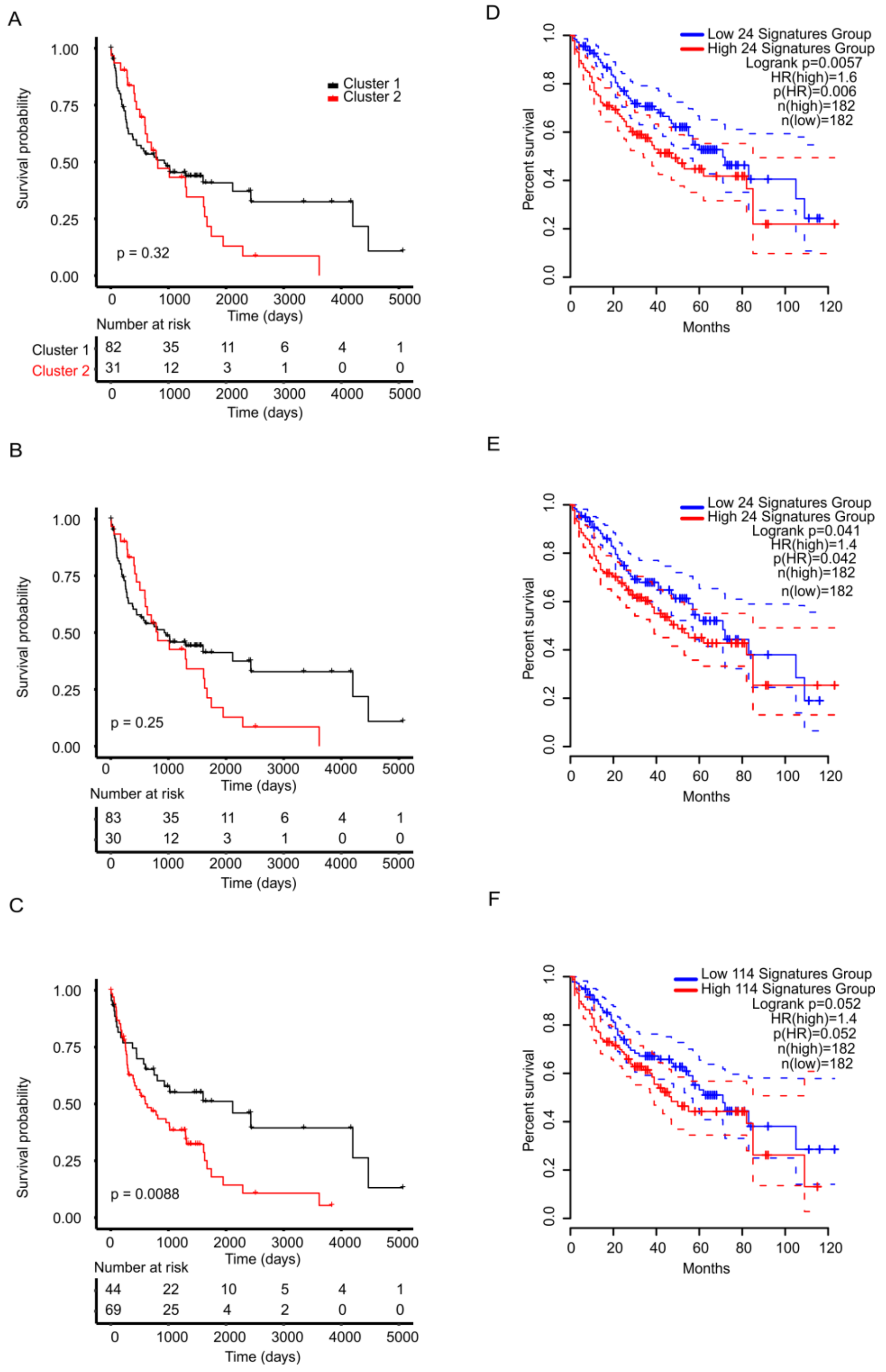

Supplemental Figure 4

A-C) Kaplan-Meyer-Analyses based on the specific transcriptome profile of Panel 1 in A., Panel 2 in B. and Panel 3 in C. in tumor tissue using public available data from authentic human HCC of 139 patients from Lee et al. cohort (24). Survival analyses were performed by CRAN package survival und survminer (version 0.4.3) using log rank tests. D-F) Kaplan-Meyer-Analyses based on the specific transcriptome profile of Panel 1 in D., Panel 2 in E. and Panel 3 in F. in tumor tissue using public available data from authentic human of the TCGA-LIHC cohort using the GEPIA.2 tool.

Supplemental Figure 5

A

ID	Associated Network Function	Score
1	Cancer, Organismal Injury and Abnormalities, Connective Tissue Development and Function	30
2	Cell Cycle, Gene Expression, Cancer	27

B

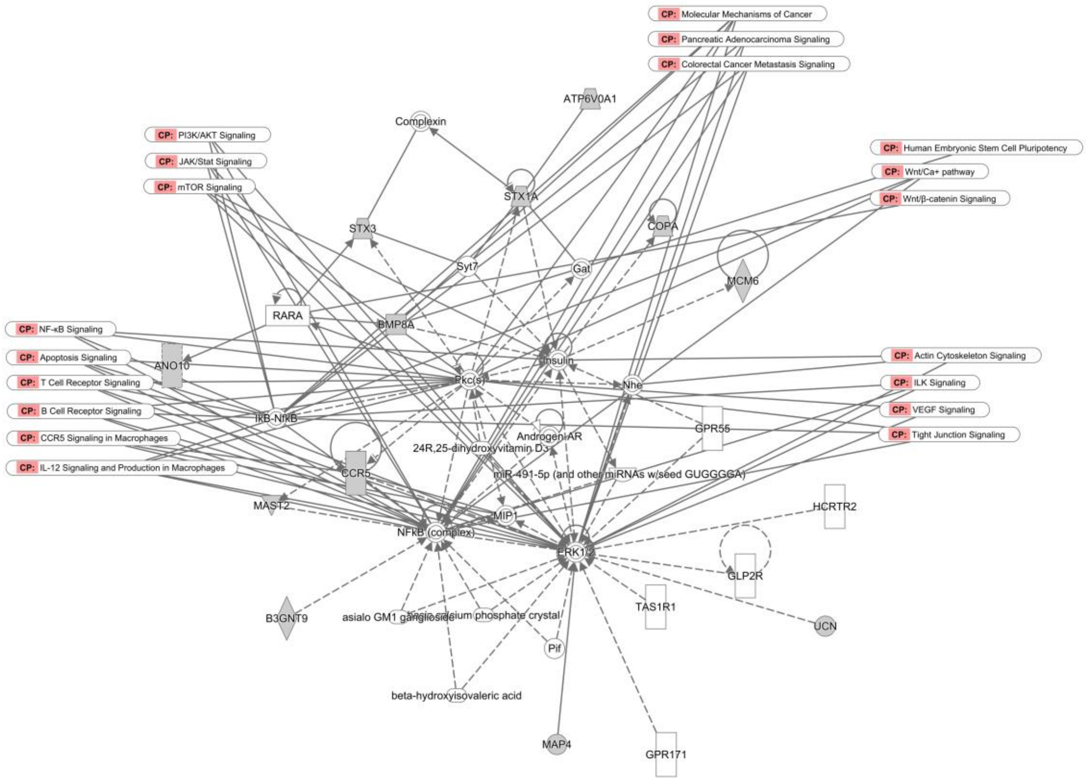

Supplemental Figure 5

A) Top Network Association of Epi-driver Panel (n=23) analyzed by ingenuity pathway analyses. B) Network of Epi-Drivers including pathway regulations analyzed and designed by ingenuity pathway analyses.

Supplemental Figure 6

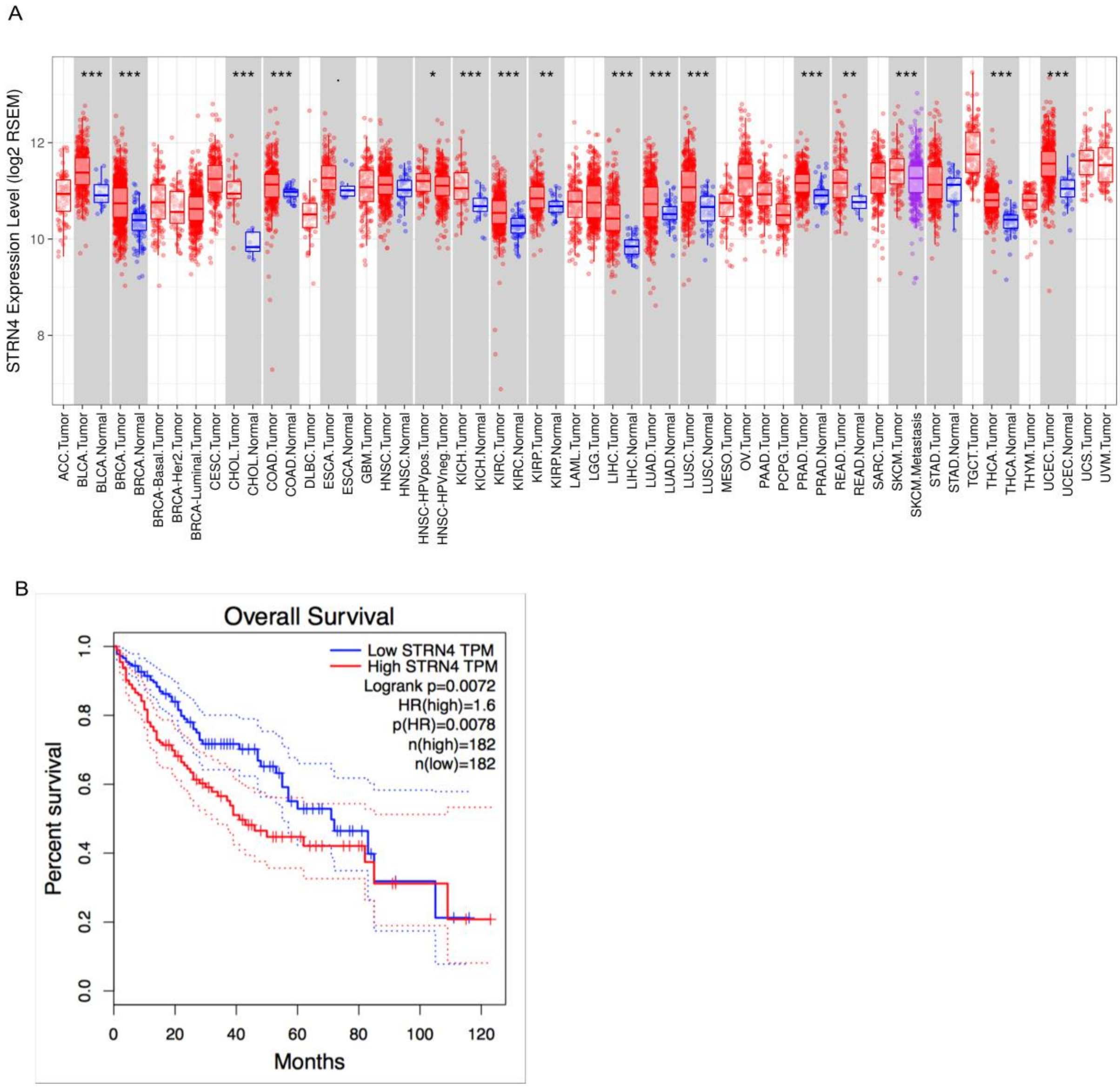

Supplemental Figure 6

A) Expression status of STRN4 in different tumor etiologies based on expression in the TCGA database using “DiffExp” module of TIMER-Tool. B) Impact on overall survival of STRN4 for HCC patients analyzed by survival analyses with a group cut-off at the median using GEPIA-Tool.

Supplemental Figure 7

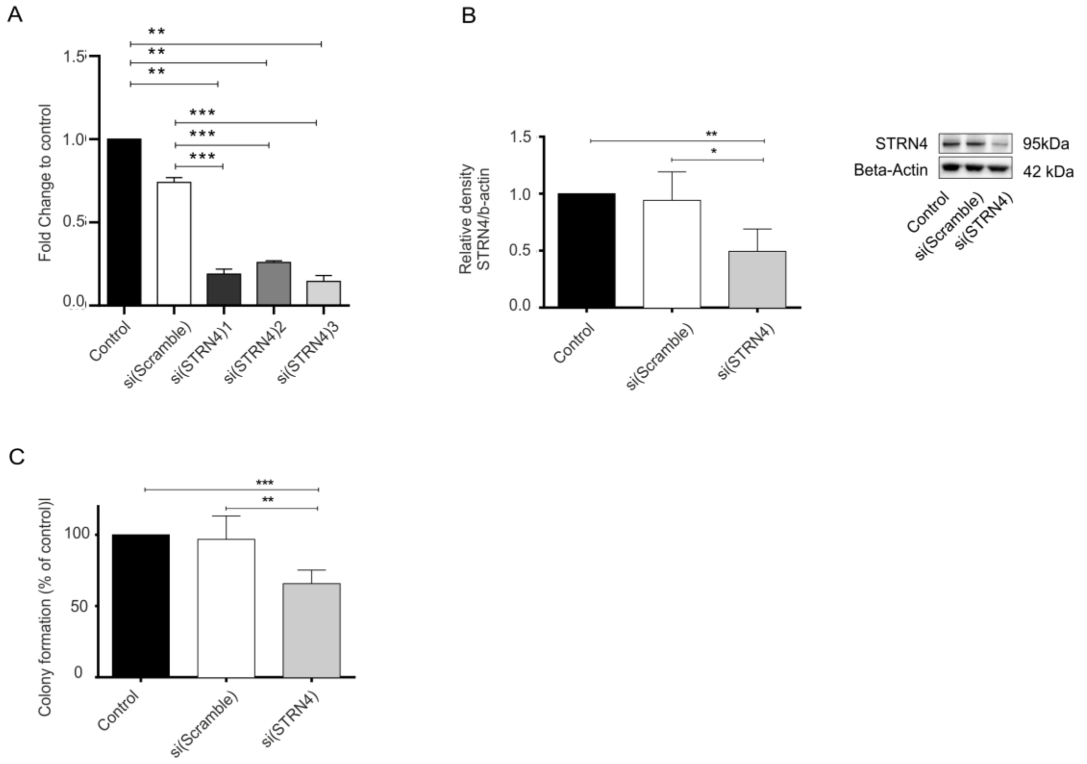

Supplemental Figure 7

A. Expression of STRN4 in Hep3B cell lines in non-treated (control), si(Scramble) and three different si(STRN4) 1-3 treated cells on mRNA level. B. Expression of STRN4 in Hep3B cell lines in non-treated (control), si(Scramble) and si(STRN4) 3 treated cells on protein level. C. Colony formation of Hep3B cell lines in non-treated (control), si(Scramble) and si(STRN4) treated cells; one-way anova analyses: *p-value <0.05; **p-value <0.01; ***p<0.001.

Supplemental Table 1: Mt-variants associated with cancer

Supplemental Table 2: Mt-variants detected by Mt-sequencing analyses

Supplemental Table 3: Signaling pathway analyses of DEG during hepatocarcinogenesis according to Figure 2A.

Supplemental Table 4: Top 100 genes of methylation and RNA-Seq-data for iclustering

Supplemental Table 5: Signaling pathway analyses of DMGR during hepatocarcinogenesis according to Figure 3.

Supplemental Table 6: DMGR signatures of early (1), late (2) hepatocarcinogenesis and of HCC progression (3).

Supplemental Table 7: Panel annotation of identified DMGR with expression changes in eHCC and/or pHCC.

Supplemental Table 8: Top network analyses and molecular and cellular functions of 162 gene signature.

Supplemental Table 9: Results on DMGR validation analyses.

Supplemental Table 10: Validation of differential expression analyses of DMGR in HCC.

Supplemental Table 11: Association to overall survival of DMGR using the GEPIA tool.

Supplemental Table 12: Multivariate analyses using the TIMER2.0 Gen_Surv module.

Supplemental Table 13: Clinico-pathological data of patients (validation cohort Mainz)

Supplemental Table 14: Large and small liver cell changes

Supplemental Table 15: Sanger validation of mitochondrial variants

Supplemental Table 16: Antibodies

Supplemental Table 1

Sample	Patient	Allele	Mutation	Count	Coverage	gene_biotype	Coding region change	Amino acid change	Site Cosmic_v70	Histology Cosmic_v70	Somatic status Cosmic_v70	Sample source Cosmic_v70	Tumour origin Cosmic_v70
T8	P3	4216	SNV	T	C	MT-ND1	MT-ND1	protein_coding	ENST00000361390:c.910T>C	Yes	Y304H, Y304H	endometrium;kidney, endometrium;kidney	clear_cell_renal_cell_carcinoma/endometrioid_carcinoma
T9	P3	4216	SNV	T	C	MT-ND1	MT-ND1	protein_coding	ENST00000361390:c.910T>C	Yes	Y304H, Y304H	endometrium;kidney, endometrium;kidney	clear_cell_renal_cell_carcinoma/endometrioid_carcinoma
T24	P2	7028	SNV	C	T	MT-CO1	MT-CO1	protein_coding	ENST00000361624:c.1125C>T	No	A375A, A375A	breast, breast	carcinoma, carcinoma
T25	P5	7028	SNV	C	T	MT-CO1	MT-CO1	protein_coding	ENST00000361624:c.1125C>T	No	A375A, A375A	breast, breast	carcinoma, carcinoma
T26	P8	7028	SNV	C	T	MT-CO1	MT-CO1	protein_coding	ENST00000361624:c.1125C>T	No	A375A, A375A	breast, breast	carcinoma, carcinoma
T1	P1	7028	SNV	C	T	MT-CO1	MT-CO1	protein_coding	ENST00000361624:c.1125C>T	No	A375A, A375A	breast, breast	carcinoma, carcinoma
T4	P2	7028	SNV	C	T	MT-CO1	MT-CO1	protein_coding	ENST00000361624:c.1125C>T	No	A375A, A375A	breast, breast	carcinoma, carcinoma
T8	P3	7028	SNV	C	T	MT-CO1	MT-CO1	protein_coding	ENST00000361624:c.1125C>T	No	A375A, A375A	breast, breast	carcinoma, carcinoma
T10	P4	7028	SNV	C	T	MT-CO1	MT-CO1	protein_coding	ENST00000361624:c.1125C>T	No	A375A, A375A	breast, breast	carcinoma, carcinoma
T12	P5	7028	SNV	C	T	MT-CO1	MT-CO1	protein_coding	ENST00000361624:c.1125C>T	No	A375A, A375A	breast, breast	carcinoma, carcinoma
T20	P6	7028	SNV	C	T	MT-CO1	MT-CO1	protein_coding	ENST00000361624:c.	No	A375A, A375A	breast, breast	carcinoma, carcinoma

									1125C>T				
T22	P7	7028	SNV	C	T	MT-CO1	MT-CO1	protein_coding	ENST00000361624:c. 1125C>T	No	A375A, A375A	breast, breast	carcinoma, carcinoma
T3	P1	7028	SNV	C	T	MT-CO1	MT-CO1	protein_coding	ENST00000361624:c. 1125C>T	No	A375A, A375A	breast, breast	carcinoma, carcinoma
T13	P5	7028	SNV	C	T	MT-CO1	MT-CO1	protein_coding	ENST00000361624:c. 1125C>T	No	A375A, A375A	breast, breast	carcinoma, carcinoma
T17	P5	7028	SNV	C	T	MT-CO1	MT-CO1	protein_coding	ENST00000361624:c. 1125C>T	No	A375A, A375A	breast, breast	carcinoma, carcinoma
T19	P5	7028	SNV	C	T	MT-CO1	MT-CO1	protein_coding	ENST00000361624:c. 1125C>T	No	A375A, A375A	breast, breast	carcinoma, carcinoma
T2	P1	7028	SNV	C	T	MT-CO1	MT-CO1	protein_coding	ENST00000361624:c. 1125C>T	No	A375A, A375A	breast, breast	carcinoma, carcinoma
T6	P2	7028	SNV	C	T	MT-CO1	MT-CO1	protein_coding	ENST00000361624:c. 1125C>T	No	A375A, A375A	breast, breast	carcinoma, carcinoma
T7	P2	7028	SNV	C	T	MT-CO1	MT-CO1	protein_coding	ENST00000361624:c. 1125C>T	No	A375A, A375A	breast, breast	carcinoma, carcinoma
T9	P3	7028	SNV	C	T	MT-CO1	MT-CO1	protein_coding	ENST00000361624:c. 1125C>T	No	A375A, A375A	breast, breast	carcinoma, carcinoma
T11	P4	7028	SNV	C	T	MT-CO1	MT-CO1	protein_coding	ENST00000361624:c. 1125C>T	No	A375A, A375A	breast, breast	carcinoma, carcinoma
T14	P5	7028	SNV	C	T	MT-CO1	MT-CO1	protein_coding	ENST00000361624:c. 1125C>T	No	A375A, A375A	breast, breast	carcinoma, carcinoma
T16	P5	7028	SNV	C	T	MT-CO1	MT-CO1	protein_coding	ENST00000361624:c. 1125C>T	No	A375A, A375A	breast, breast	carcinoma, carcinoma
T18	P5	7028	SNV	C	T	MT-CO1	MT-CO1	protein_coding	ENST00000361624:c. 1125C>T	No	A375A, A375A	breast, breast	carcinoma, carcinoma
T28	P8	7028	SNV	C	T	MT-CO1	MT-CO1	protein_coding	ENST00000361624:c. 1125C>T	No	A375A, A375A	breast, breast	carcinoma, carcinoma
T5	P2	7028	SNV	C	T	MT-CO1	MT-CO1	protein_coding	ENST00000361624:c. 1125C>T	No	A375A, A375A	breast, breast	carcinoma, carcinoma
T15	P5	7028	SNV	C	T	MT-CO1	MT-CO1	protein_coding	ENST00000361624:c. 1125C>T	No	A375A, A375A	breast, breast	carcinoma, carcinoma

T21	P6	7028	SNV	C	T	MT-CO1	MT-CO1	protein_coding	ENST00000361624:c.1125C>T	No	A375A, A375A	breast, breast	carcinoma, carcinoma
T23	P7	7028	SNV	C	T	MT-CO1	MT-CO1	protein_coding	ENST00000361624:c.1125C>T	No	A375A, A375A	breast, breast	carcinoma, carcinoma
T27	P8	7028	SNV	C	T	MT-CO1	MT-CO1	protein_coding	ENST00000361624:c.1125C>T	No	A375A, A375A	breast, breast	carcinoma, carcinoma
T20	P6	8200	SNV	T	C	MT-CO2	MT-CO2	protein_coding	ENST00000361739:c.615T>C	No	S205S	kidney	carcinoma/clear_cell_renal_cell_carcinoma
T3	P1	8200	SNV	T	C	MT-CO2	MT-CO2	protein_coding	ENST00000361739:c.615T>C	No	S205S	kidney	carcinoma/clear_cell_renal_cell_carcinoma
T2	P1	8200	SNV	T	C	MT-CO2	MT-CO2	protein_coding	ENST00000361739:c.615T>C	No	S205S	kidney	carcinoma/clear_cell_renal_cell_carcinoma
T21	P6	8200	SNV	T	C	MT-CO2	MT-CO2	protein_coding	ENST00000361739:c.615T>C	No	S205S	kidney	carcinoma/clear_cell_renal_cell_carcinoma
T24	P2	8701	SNV	A	G	MT-ATP6	MT-ATP6	protein_coding	ENST00000361899:c.175A>G	Yes	T59A, T59A	breast, breast	carcinoma, carcinoma
T26	P8	8701	SNV	A	G	MT-ATP6	MT-ATP6	protein_coding	ENST00000361899:c.175A>G	Yes	T59A, T59A	breast, breast	carcinoma, carcinoma
T4	P2	8701	SNV	A	G	MT-ATP6	MT-ATP6	protein_coding	ENST00000361899:c.175A>G	Yes	T59A, T59A	breast, breast	carcinoma, carcinoma
T8	P3	8701	SNV	A	G	MT-ATP6	MT-ATP6	protein_coding	ENST00000361899:c.175A>G	Yes	T59A, T59A	breast, breast	carcinoma, carcinoma
T10	P4	8701	SNV	A	G	MT-ATP6	MT-ATP6	protein_coding	ENST00000361899:c.175A>G	Yes	T59A, T59A	breast, breast	carcinoma, carcinoma
T20	P6	8701	SNV	A	G	MT-ATP6	MT-ATP6	protein_coding	ENST00000361899:c.175A>G	Yes	T59A, T59A	breast, breast	carcinoma, carcinoma
T3	P1	8701	SNV	A	G	MT-ATP6	MT-ATP6	protein_coding	ENST00000361899:c.175A>G	Yes	T59A, T59A	breast, breast	carcinoma, carcinoma
T2	P1	8701	SNV	A	G	MT-ATP6	MT-ATP6	protein_coding	ENST00000361899:c.	Yes	T59A, T59A	breast, breast	carcinoma, carcinoma

									175A>G				
T6	P2	8701	SNV	A	G	MT-ATP6	MT-ATP6	protein_coding	ENST00000361899:c. 175A>G	Yes	T59A, T59A	breast, breast	carcinoma, carcinoma
T7	P2	8701	SNV	A	G	MT-ATP6	MT-ATP6	protein_coding	ENST00000361899:c. 175A>G	Yes	T59A, T59A	breast, breast	carcinoma, carcinoma
T9	P3	8701	SNV	A	G	MT-ATP6	MT-ATP6	protein_coding	ENST00000361899:c. 175A>G	Yes	T59A, T59A	breast, breast	carcinoma, carcinoma
T11	P4	8701	SNV	A	G	MT-ATP6	MT-ATP6	protein_coding	ENST00000361899:c. 175A>G	Yes	T59A, T59A	breast, breast	carcinoma, carcinoma
T28	P8	8701	SNV	A	G	MT-ATP6	MT-ATP6	protein_coding	ENST00000361899:c. 175A>G	Yes	T59A, T59A	breast, breast	carcinoma, carcinoma
T5	P2	8701	SNV	A	G	MT-ATP6	MT-ATP6	protein_coding	ENST00000361899:c. 175A>G	Yes	T59A, T59A	breast, breast	carcinoma, carcinoma
T21	P6	8701	SNV	A	G	MT-ATP6	MT-ATP6	protein_coding	ENST00000361899:c. 175A>G	Yes	T59A, T59A	breast, breast	carcinoma, carcinoma
T27	P8	8701	SNV	A	G	MT-ATP6	MT-ATP6	protein_coding	ENST00000361899:c. 175A>G	Yes	T59A, T59A	breast, breast	carcinoma, carcinoma
T22	P7	9123	SNV	G	A	MT-ATP6	MT-ATP6	protein_coding	ENST00000361899:c. 597G>A	No	L199L, L199L	endometrium, endometrium	carcinoma/endometrioid_carcinoma, carcinoma/endometrioid_carcinoma
T23	P7	9123	SNV	G	A	MT-ATP6	MT-ATP6	protein_coding	ENST00000361899:c. 597G>A	No	L199L, L199L	endometrium, endometrium	carcinoma/endometrioid_carcinoma, carcinoma/endometrioid_carcinoma
T19	P5	10197	SNV	G	A	MT-ND3	MT-ND3	protein_coding	ENST00000361227:c. 139G>A	Yes	A47T	breast	carcinoma
T24	P2	11719	SNV	G	A	MT-ND4	MT-ND4	protein_coding	ENST00000361381:c. 960G>A	No	G320G, G320G	breast, breast	carcinoma, carcinoma
T25	P5	11719	SNV	G	A	MT-ND4	MT-ND4	protein_coding	ENST00000361381:c.	No	G320G, G320G	breast, breast	carcinoma, carcinoma

									960G>A				
T26	P8	11719	SNV	G	A	MT-ND4	MT-ND4	protein_coding	ENST00000361381:c.960G>A	No	G320G, G320G	breast, breast	carcinoma, carcinoma
T1	P1	11719	SNV	G	A	MT-ND4	MT-ND4	protein_coding	ENST00000361381:c.960G>A	No	G320G, G320G	breast, breast	carcinoma, carcinoma
T4	P2	11719	SNV	G	A	MT-ND4	MT-ND4	protein_coding	ENST00000361381:c.960G>A	No	G320G, G320G	breast, breast	carcinoma, carcinoma
T8	P3	11719	SNV	G	A	MT-ND4	MT-ND4	protein_coding	ENST00000361381:c.960G>A	No	G320G, G320G	breast, breast	carcinoma, carcinoma
T10	P4	11719	SNV	G	A	MT-ND4	MT-ND4	protein_coding	ENST00000361381:c.960G>A	No	G320G, G320G	breast, breast	carcinoma, carcinoma
T12	P5	11719	SNV	G	A	MT-ND4	MT-ND4	protein_coding	ENST00000361381:c.960G>A	No	G320G, G320G	breast, breast	carcinoma, carcinoma
T20	P6	11719	SNV	G	A	MT-ND4	MT-ND4	protein_coding	ENST00000361381:c.960G>A	No	G320G, G320G	breast, breast	carcinoma, carcinoma
T22	P7	11719	SNV	G	A	MT-ND4	MT-ND4	protein_coding	ENST00000361381:c.960G>A	No	G320G, G320G	breast, breast	carcinoma, carcinoma
T3	P1	11719	SNV	G	A	MT-ND4	MT-ND4	protein_coding	ENST00000361381:c.960G>A	No	G320G, G320G	breast, breast	carcinoma, carcinoma
T13	P5	11719	SNV	G	A	MT-ND4	MT-ND4	protein_coding	ENST00000361381:c.960G>A	No	G320G, G320G	breast, breast	carcinoma, carcinoma
T17	P5	11719	SNV	G	A	MT-ND4	MT-ND4	protein_coding	ENST00000361381:c.960G>A	No	G320G, G320G	breast, breast	carcinoma, carcinoma
T19	P5	11719	SNV	G	A	MT-ND4	MT-ND4	protein_coding	ENST00000361381:c.960G>A	No	G320G, G320G	breast, breast	carcinoma, carcinoma
T2	P1	11719	SNV	G	A	MT-ND4	MT-ND4	protein_coding	ENST00000361381:c.960G>A	No	G320G, G320G	breast, breast	carcinoma, carcinoma
T6	P2	11719	SNV	G	A	MT-ND4	MT-ND4	protein_coding	ENST00000361381:c.960G>A	No	G320G, G320G	breast, breast	carcinoma, carcinoma
T7	P2	11719	SNV	G	A	MT-ND4	MT-ND4	protein_coding	ENST00000361381:c.960G>A	No	G320G, G320G	breast, breast	carcinoma, carcinoma
T9	P3	11719	SNV	G	A	MT-ND4	MT-ND4	protein_coding	ENST00000361381:c.960G>A	No	G320G, G320G	breast, breast	carcinoma, carcinoma

T11	P4	11719	SNV	G	A	MT-ND4	MT-ND4	protein_coding	ENST00000361381:c.960G>A	No	G320G, G320G	breast, breast	carcinoma, carcinoma
T14	P5	11719	SNV	G	A	MT-ND4	MT-ND4	protein_coding	ENST00000361381:c.960G>A	No	G320G, G320G	breast, breast	carcinoma, carcinoma
T16	P5	11719	SNV	G	A	MT-ND4	MT-ND4	protein_coding	ENST00000361381:c.960G>A	No	G320G, G320G	breast, breast	carcinoma, carcinoma
T18	P5	11719	SNV	G	A	MT-ND4	MT-ND4	protein_coding	ENST00000361381:c.960G>A	No	G320G, G320G	breast, breast	carcinoma, carcinoma
T28	P8	11719	SNV	G	A	MT-ND4	MT-ND4	protein_coding	ENST00000361381:c.960G>A	No	G320G, G320G	breast, breast	carcinoma, carcinoma
T5	P2	11719	SNV	G	A	MT-ND4	MT-ND4	protein_coding	ENST00000361381:c.960G>A	No	G320G, G320G	breast, breast	carcinoma, carcinoma
T15	P5	11719	SNV	G	A	MT-ND4	MT-ND4	protein_coding	ENST00000361381:c.960G>A	No	G320G, G320G	breast, breast	carcinoma, carcinoma
T21	P6	11719	SNV	G	A	MT-ND4	MT-ND4	protein_coding	ENST00000361381:c.960G>A	No	G320G, G320G	breast, breast	carcinoma, carcinoma
T23	P7	11719	SNV	G	A	MT-ND4	MT-ND4	protein_coding	ENST00000361381:c.960G>A	No	G320G, G320G	breast, breast	carcinoma, carcinoma
T27	P8	11719	SNV	G	A	MT-ND4	MT-ND4	protein_coding	ENST00000361381:c.960G>A	No	G320G, G320G	breast, breast	carcinoma, carcinoma
T24	P2	12705	SNV	C	T	MT-ND5	MT-ND5	protein_coding	ENST00000361567:c.369C>T	No	I123I	endometrium	carcinoma/endometrioid_carcinoma
T26	P8	12705	SNV	C	T	MT-ND5	MT-ND5	protein_coding	ENST00000361567:c.369C>T	No	I123I	endometrium	carcinoma/endometrioid_carcinoma
T1	P1	12705	SNV	C	T	MT-ND5	MT-ND5	protein_coding	ENST00000361567:c.369C>T	No	I123I	endometrium	carcinoma/endometrioid_carcinoma
T4	P2	12705	SNV	C	T	MT-ND5	MT-ND5	protein_coding	ENST00000361567:c.369C>T	No	I123I	endometrium	carcinoma/endometrioid_carcinoma
T8	P3	12705	SNV	C	T	MT-ND5	MT-ND5	protein_coding	ENST00000361567:c.369C>T	No	I123I	endometrium	carcinoma/endometrioid_carcinoma

T10	P4	12705	SNV	C	T	MT-ND5	MT-ND5	protein_coding	ENST00000361567:c.369C>T	No	I123I	endometrium	carcinoma/endometrioid_carcinoma
T20	P6	12705	SNV	C	T	MT-ND5	MT-ND5	protein_coding	ENST00000361567:c.369C>T	No	I123I	endometrium	carcinoma/endometrioid_carcinoma
T3	P1	12705	SNV	C	T	MT-ND5	MT-ND5	protein_coding	ENST00000361567:c.369C>T	No	I123I	endometrium	carcinoma/endometrioid_carcinoma
T2	P1	12705	SNV	C	T	MT-ND5	MT-ND5	protein_coding	ENST00000361567:c.369C>T	No	I123I	endometrium	carcinoma/endometrioid_carcinoma
T6	P2	12705	SNV	C	T	MT-ND5	MT-ND5	protein_coding	ENST00000361567:c.369C>T	No	I123I	endometrium	carcinoma/endometrioid_carcinoma
T7	P2	12705	SNV	C	T	MT-ND5	MT-ND5	protein_coding	ENST00000361567:c.369C>T	No	I123I	endometrium	carcinoma/endometrioid_carcinoma
T9	P3	12705	SNV	C	T	MT-ND5	MT-ND5	protein_coding	ENST00000361567:c.369C>T	No	I123I	endometrium	carcinoma/endometrioid_carcinoma
T11	P4	12705	SNV	C	T	MT-ND5	MT-ND5	protein_coding	ENST00000361567:c.369C>T	No	I123I	endometrium	carcinoma/endometrioid_carcinoma
T28	P8	12705	SNV	C	T	MT-ND5	MT-ND5	protein_coding	ENST00000361567:c.369C>T	No	I123I	endometrium	carcinoma/endometrioid_carcinoma
T5	P2	12705	SNV	C	T	MT-ND5	MT-ND5	protein_coding	ENST00000361567:c.369C>T	No	I123I	endometrium	carcinoma/endometrioid_carcinoma
T21	P6	12705	SNV	C	T	MT-ND5	MT-ND5	protein_coding	ENST00000361567:c.369C>T	No	I123I	endometrium	carcinoma/endometrioid_carcinoma
T27	P8	12705	SNV	C	T	MT-ND5	MT-ND5	protein_coding	ENST00000361567:c.369C>T	No	I123I	endometrium	carcinoma/endometrioid_carcinoma
T20	P6	14569	SNV	G	A	MT-ND6	MT-ND6	protein_coding	ENST00000361681:c.105C>T	No	S35S, S35S	kidney, kidney	carcinoma/clear_cell_renal_cell_carcinoma, carcinoma/clear_cell_renal_cell_carcinoma
T3	P1	14569	SNV	G	A	MT-ND6	MT-ND6	protein_coding	ENST00000361681:c.105C>T	No	S35S, S35S	kidney, kidney	carcinoma/clear_cell_renal_cell_carcinoma, carcinoma/clear_cell_renal_cell_carcinoma

T2	P1	14569	SNV	G	A	MT-ND6	MT-ND6	protein_coding	ENST00000361681:c.105C>T	No	S35S, S35S	kidney, kidney	carcinoma/clear_cell_renal_cell_carcinoma, carcinoma/clear_cell_renal_cell_carcinoma
T21	P6	14569	SNV	G	A	MT-ND6	MT-ND6	protein_coding	ENST00000361681:c.105C>T	No	S35S, S35S	kidney, kidney	carcinoma/clear_cell_renal_cell_carcinoma, carcinoma/clear_cell_renal_cell_carcinoma
T10	P4	15045	SNV	G	A	MT-CYB	MT-CYB	protein_coding	ENST00000361789:c.299G>A	Yes	R100Q, R100Q	kidney, kidney	carcinoma/clear_cell_renal_cell_carcinoma, carcinoma/clear_cell_renal_cell_carcinoma
T20	P6	15323	SNV	G	A	MT-CYB	MT-CYB	protein_coding	ENST00000361789:c.577G>A	Yes	A193T, A193T	breast, breast	carcinoma, carcinoma
T3	P1	15323	SNV	G	A	MT-CYB	MT-CYB	protein_coding	ENST00000361789:c.577G>A	Yes	A193T, A193T	breast, breast	carcinoma, carcinoma
T2	P1	15323	SNV	G	A	MT-CYB	MT-CYB	protein_coding	ENST00000361789:c.577G>A	Yes	A193T, A193T	breast, breast	carcinoma, carcinoma
T21	P6	15323	SNV	G	A	MT-CYB	MT-CYB	protein_coding	ENST00000361789:c.577G>A	Yes	A193T, A193T	breast, breast	carcinoma, carcinoma

Supplemental Table 2

Patient	Sample ID	Diagnosis	MT-Region	Mutation Type	Ref. Allele	Mut. Allele	Frequency	Gene Cards	Coding region change	Amino acid change	Non-synonym
1	T3	HGDN	11866^11867	Insertion	-	C	5,162622612	MT-ND4	ENST00000361381:c.1107_1108insC	ENSP00000354961:p.Thr372fs	Yes
1	T2	LGDN	12418	Deletion	A	-	6,059390048	MT-ND5	ENST00000361567:c.82delA	ENSP00000354813:p.Asn30fs	Yes
1	T2	LGDN	15050	Replacement	C	AGA	7,404844291	MT-CYB	ENST00000361789:c.304delCinsAGA	ENSP00000354554:p.Leu102fs	Yes
1	T2	LGDN	13958	SNV	G	A	9,875690608	MT-ND5	ENST00000361567:c.1622G>A	ENSP00000354813:p.Gly541Asp	Yes
1	T3	HGDN	310	SNV	T	C	14,6031746				-
1	T2	LGDN	310	SNV	T	C	16,11721612				-
1	T2	LGDN	899	SNV	G	A	18,18471338	MT-RNR1			-
1	T1	CL	302^303	Insertion	-	CC	59,15492958				-
1	T3	HGDN	8701	SNV	A	G	78,91804457	MT-ATP6	ENST00000361899:c.175A>G	ENSP00000354632:p.Thr59Ala	Yes
1	T2	LGDN	8701	SNV	A	G	79,06643541	MT-ATP6	ENST00000361899:c.175A>G	ENSP00000354632:p.Thr59Ala	Yes
1	T2	LGDN	2706	SNV	A	G	94,03892944	MT-RNR2			-
1	T3	HGDN	2706	SNV	A	G	94,47300771	MT-RNR2			-
1	T1	CL	2706	SNV	A	G	95,0902387	MT-RNR2			-
2	T4	CL	15050	Replacement	C	AGA	5,151086764	MT-CYB	ENST00000361789:c.304delCinsAGA	ENSP00000354554:p.Leu102fs	Yes
2	T4	CL	12823	SNV	G	A	6,327160494	MT-ND5	ENST00000361567:c.487G>A	ENSP00000354813:p.Asp163Asn	Yes
2	T5	HGDN	1210	SNV	T	C	6,388459557	MT-RNR1			-
2	T4	CL	57	Deletion	T	-	6,891175553				-
2	T5	HGDN	310	SNV	T	C	10,17964072				-
2	T4	CL	14518	SNV	A	G	16,37931034	MT-ND6	ENST00000361681:c.156T>C		No

2	T5	HGDN	14518	SNV	A	G	18,19990055	MT-ND6	ENST00000361681:c.156T>C		No
2	T6	HGDN	14518	SNV	A	G	39,30276982	MT-ND6	ENST00000361681:c.156T>C		No
2	T5	HGDN	16145	SNV	G	A	52,05681609				-
2	T5	eHCC	310..316	Deletion	TCCCCCG	-	62,93577982				-
2	T7	eHCC	319	SNV	T	C	65,81196581				-
2	T5	HGDN	302^303	Insertion	-	C	70,81174439				-
2	T4	CL	302^303	Insertion	-	C	71,24183007				-
2	T8	pHCC	302^303	Insertion	-	C	76,27737226				-
2	T8	pHCC	8701	SNV	A	G	77,19760129	MT-ATP6	ENST00000361899:c.175A>G	ENSP00000354632:p.Thr59Ala	Yes
2	T6	HGDN	302^303	Insertion	-	C	77,42663657				-
2	T7	eHCC	8701	SNV	A	G	79,35022355	MT-ATP6	ENST00000361899:c.175A>G	ENSP00000354632:p.Thr59Ala	Yes
2	T6	HGDN	8701	SNV	A	G	79,42515864	MT-ATP6	ENST00000361899:c.175A>G	ENSP00000354632:p.Thr59Ala	Yes
2	T4	CL	8701	SNV	A	G	84,45665188	MT-ATP6	ENST00000361899:c.175A>G	ENSP00000354632:p.Thr59Ala	Yes
2	T5	HGDN	8701	SNV	A	G	84,84073002	MT-ATP6	ENST00000361899:c.175A>G	ENSP00000354632:p.Thr59Ala	Yes
2	T4	CL	2706	SNV	A	G	93,96306818	MT-RNR2			-
2	T7	eHCC	2706	SNV	A	G	93,96507003	MT-RNR2			-
2	T6	HGDN	2706	SNV	A	G	94,31937724	MT-RNR2			-
2	T5	HGDN	2706	SNV	A	G	94,34803375	MT-RNR2			-
2	T8	pHCC	2706	SNV	A	G	94,95242036	MT-RNR2			-
3	T10	HGDN	12236	SNV	G	A	5,226174791	MT-TS2			-
3	T9	CL	16189^16190	Insertion	-	C	6,731813246				-
3	T9	CL	15050	Replacement	C	AGA	7,775951448	MT-CYB	ENST00000361789:c.304delCinsAGA	ENSP00000354554:p.Leu102fs	Yes

3	T10	HGDN	310	SNV	T	C	11,2804878				-
3	T10	HGDN	5400	SNV	G	A	25,01781896	MT-ND2	ENST00000361453:c.931G>A	ENSP00000355046:p.Val311Met	Yes
3	T10	HGDN	8701	SNV	A	G	79,20731291	MT-ATP6	ENST00000361899:c.175A>G	ENSP00000354632:p.Thr59Ala	Yes
3	T9	CL	8701	SNV	A	G	81,46943541	MT-ATP6	ENST00000361899:c.175A>G	ENSP00000354632:p.Thr59Ala	Yes
3	T9	CL	16183^16184	Insertion	-	C	93,7133724				-
3	T10	HGDN	2706	SNV	A	G	93,9107903	MT-RNR2			-
3	T10	HGDN	16183^16184	Insertion	-	C	94,17062797				-
3	T9	CL	2706	SNV	A	G	94,6	MT-RNR2			-
3	T10	HGDN	16189^16190	Insertion	-	C	94,94640123				-
4	T12	HGDN	70	SNV	G	A	5,289421158				-
4	T11	CL	15045	SNV	G	A	7,130305488	MT-CYB	ENST00000361789:c.299G>A	ENSP00000354554:p.Arg100Gln	Yes
4	T11	CL	1608	SNV	G	A	8,428093645	MT-TV			-
4	T11	CL	310^311	Insertion	-	C	12,3388582				-
4	T11	CL	302	Replacement	A	CC	18,19354839				-
4	T11	CL	302	SNV	A	C	30,83870968				-
4	T12	HGDN	302	Replacement	A	CC	32,3943662				-
4	T11	CL	8701	SNV	A	G	80,13850416	MT-ATP6	ENST00000361899:c.175A>G	ENSP00000354632:p.Thr59Ala	Yes
4	T12	HGDN	8701	SNV	A	G	81,78076554	MT-ATP6	ENST00000361899:c.175A>G	ENSP00000354632:p.Thr59Ala	Yes
4	T12	HGDN	310	SNV	T	C	84,47488584				-
4	T12	HGDN	2706	SNV	A	G	93,62859363	MT-RNR2			-
4	T11	CL	2706	SNV	A	G	93,71618083	MT-RNR2			-
4	T11	CL	310	SNV	T	C	93,94736842				-
5	T14	LGDN	16129	SNV	G	A	5,438666137				-

5	T16	LGDN	6582	SNV	G	A	5,882352941	MT-CO1	ENST00000361624:c.679G>A	ENSP00000354499:p.Asp227Asn	Yes
5	T19	HGDN	3778	SNV	G	A	5,941389	MT-ND1	ENST00000361390:c.472G>A	ENSP00000354687:p.Gly158Ser	Yes
5	T16	LGDN	3550	SNV	G	A	6,092368162	MT-ND1	ENST00000361390:c.244G>A	ENSP00000354687:p.Ala82Thr	Yes
5	T13	CL	8902	SNV	G	A	7,976961843	MT-ATP6	ENST00000361899:c.376G>A	ENSP00000354632:p.Ala126Thr	Yes
5	T21	pHCC	14944	SNV	C	A	8,037773142	MT-CYB	ENST00000361789:c.198C>A	ENSP00000354554:p.Ile66Met	Yes
5	T18	HGDN	4418	SNV	T	C	8,090768682	MT-TM			-
5	T14	LGDN	4037	SNV	G	A	10,37924152	MT-ND1	ENST00000361390:c.731G>A	ENSP00000354687:p.Gly244Glu	Yes
5	T13	CL	1045	SNV	G	A	16,82058047	MT-RNR1			-
5	T17	HGDN	16108	SNV	C	T	17,65292553				-
5	T16	LGDN	10197	SNV	G	A	67,53975678	MT-ND3	ENST00000361227:c.139G>A	ENSP00000355206:p.Ala47Thr	Yes
5	T13	CL	302^303	Insertion	-	C	78,37078652				-
5	T16	LGDN	302^303	Insertion	-	C	79,66101695				-
5	T19	HGDN	302^303	Insertion	-	C	79,85074627				-
5	T14	LGDN	302^303	Insertion	-	C	82,90909091				-
5	T17	HGDN	310^311	Insertion	-	C	89,20863309				-
5	T16	LGDN	514..515	Deletion	CA	-	93,22916667				-
5	T17	HGDN	2706	SNV	A	G	93,42530085	MT-RNR2			-
5	T19	HGDN	2706	SNV	A	G	93,57208448	MT-RNR2			-
5	T16	LGDN	2706	SNV	A	G	93,65689572	MT-RNR2			-
5	T21	pHCC	2706	SNV	A	G	93,76884422	MT-RNR2			-
5	T18	HGDN	2706	SNV	A	G	93,83378016	MT-RNR2			-
5	T15	LGDN	2706	SNV	A	G	93,83378016	MT-RNR2			-
5	T19	HGDN	514..515	Deletion	CA	-	94,1362916				-
5	T14	LGDN	2706	SNV	A	G	94,19525066	MT-RNR2			-
5	T17	HGDN	514..515	Deletion	CA	-	94,22799423				-

5	T13	CL	2706	SNV	A	G	94,84213063	MT-RNR2			-
5	T20	eHCC	2706	SNV	A	G	95,02913953	MT-RNR2			-
5	T13	CL	514..515	Deletion	CA	-	95,05178366				-
5	T20	eHCC	514..515	Deletion	CA	-	95,12635379				-
5	T18	HGDN	514..515	Deletion	CA	-	95,52572707				-
6	T23	eHCC	15050	Replacement	C	AGA	6,896551724	MT-CYB	ENST00000361789:c.<br>304delCinsAGA	ENSP00000354554:<br>p.Leu102fs	Yes
6	T22	CL	203	SNV	G	A	13,93293475				-
6	T22	CL	5579	SNV	G	A	20,53571429	MT-TW			-
6	T22	CL	15650	SNV	G	A	21,20945134	MT-CYB	ENST00000361789:c.<br>904G>A	ENSP00000354554:<br>p.Ala302Thr	Yes
6	T22	CL	514..515	Deletion	CA	-	39,30434783				-
6	T23	eHCC	16519	SNV	T	C	46,65948276				-
6	T22	CL	8701	SNV	A	G	76,13513837	MT-ATP6	ENST00000361899:c.<br>175A>G	ENSP00000354632:<br>p.Thr59Ala	Yes
6	T23	eHCC	302^303	Insertion	-	C	81,73374613				-
6	T23	eHCC	8701	SNV	A	G	81,85952501	MT-ATP6	ENST00000361899:c.<br>175A>G	ENSP00000354632:<br>p.Thr59Ala	Yes
6	T23	eHCC	2706	SNV	A	G	94,26917511	MT-RNR2			-
6	T22	CL	2706	SNV	A	G	94,96717724	MT-RNR2			-
7	T24	CL	516..517	Replacement	CA	T	5,667276051				-
7	T24	CL	3779	SNV	G	A	6,036121673	MT-ND1	ENST00000361390:c.<br>473G>A	ENSP00000354687:<br>p.Gly158Asp	Yes
7	T25	eHCC	16180..16<br>181	Deletion	AA	-	7,122507123				-
7	T24	CL	16180..16<br>181	Deletion	AA	-	7,931316435				-
7	T24	CL	72..73	MNV	TA	CG	13,10904872				-
7	T25	eHCC	16182..16<br>183	MNV	AA	CC	14,12103746				-
7	T24	CL	16182..16<br>183	MNV	AA	CC	18,96974652				-

7	T24	CL	13965	SNV	T	C	22,76803119	MT-ND5	ENST00000361567:c.<br>1629T>C		No
7	T24	CL	16183	SNV	A	C	66,14881439				-
7	T25	eHCC	16180	Deletion	A	-	67,14150047				-
7	T24	CL	16180	Deletion	A	-	68,35650041				-
7	T25	eHCC	8271..827<br>9	Deletion	ACCCC<br>CTCT	-	68,75				-
7	T25	eHCC	302^303	Insertion	-	C	72,77486911				-
7	T25	eHCC	16183	SNV	A	C	73,9673391				-
7	T24	CL	73	SNV	A	G	86,75174014				-
7	T25	eHCC	2706	SNV	A	G	92,45614035	MT-RNR2			-
7	T24	CL	514..515	Deletion	CA	-	92,73743017				-
7	T25	eHCC	514..515	Deletion	CA	-	93,19470699				-
7	T24	CL	2706	SNV	A	G	93,82463323	MT-RNR2			-
7	T25	eHCC	16189	SNV	T	C	95,44419134				-
7	T25	eHCC	16154	SNV	T	C	96,41185647				-
8	T26	HGDN	15170	SNV	G	A	6,635273973	MT-CYB	ENST00000361789:c.<br>424G>A	ENSP00000354554:<br>p.Gly142*	Yes
8	T26	HGDN	8903	SNV	C	T	23,03688617	MT-ATP6	ENST00000361899:c.<br>377C>T	ENSP00000354632:<br>p.Ala126Val	Yes
8	T26	HGDN	4109	SNV	C	T	37,29903537	MT-ND1	ENST00000361390:c.<br>803C>T	ENSP00000354687:<br>p.Ser268Phe	Yes
8	T28	pHCC	302^303	Insertion	-	C	73,29192547				-
8	T28	pHCC	8701	SNV	A	G	75,65775169	MT-ATP6	ENST00000361899:c.<br>175A>G	ENSP00000354632:<br>p.Thr59Ala	Yes
8	T27	eHCC	8701	SNV	A	G	77,11769813	MT-ATP6	ENST00000361899:c.<br>175A>G	ENSP00000354632:<br>p.Thr59Ala	Yes
8	T26	HGDN	8701	SNV	A	G	77,7721583	MT-ATP6	ENST00000361899:c.<br>175A>G	ENSP00000354632:<br>p.Thr59Ala	Yes
8	T28	pHCC	2706	SNV	A	G	93,55503237	MT-RNR2			-
8	T27	eHCC	2706	SNV	A	G	94,61031408	MT-RNR2			-
8	T26	HGDN	2706	SNV	A	G	95,11726259	MT-RNR2			-

Supplemental Table 3

Top 100 Genes - Methylation-Data			Top 100 Genes - RNA-Seq-Data		
1	cg04773745	<NA>	1	ENSG00000138778	CENPE
2	cg04341210	C13orf36	2	ENSG00000270728	RP4-657E11.10
3	cg10916429	ZNF609	3	ENSG00000152760	TCTEX1D1
4	cg05766510	PTPRN2	4	ENSG00000183054	RGPD6
5	cg18620300	FBXL7	5	ENSG00000189334	S100A14
6	cg02514000	WBSCR17	6	ENSG00000232956	SNHG15
7	cg04227077	<NA>	7	ENSG00000107807	TLX1
8	cg05765580	NOS2	8	ENSG00000237978	RP11-385J1.2
9	cg27430369	<NA>	9	ENSG00000204832	ST8SIA6-AS1
10	cg04515534	RADIL	10	ENSG00000107187	LHX3
11	cg00949578	C18orf62	11	ENSG00000232233	RP11-573D15.2
12	cg08611472	LOC285696	12	ENSG00000124564	SLC17A3
13	cg17836612	LGALS3BP	13	ENSG00000126838	PZP
14	cg06720232	SDK1	14	ENSG00000009694	TENM1
15	cg12680822	NCRNA00029	15	ENSG00000196367	TRRAP
16	cg26150533	FABP4	16	ENSG00000214022	REPIN1
17	cg10173787	GPR35	17	ENSG00000265735	RN7SL5P
18	cg04350355	CACNG2	18	ENSG00000074276	CDHR2
19	cg09676669	AQP1	19	ENSG00000009954	BAZ1B
20	cg24500428	PTPRN2	20	ENSG00000144136	SLC20A1
21	cg17623116	LRRC3B	21	ENSG00000106031	HOXA13
22	cg15825065	LRRC6	22	ENSG00000128602	SMO
23	cg10257673	PTPRN2	23	ENSG00000166900	STX3
24	cg22165480	<NA>	24	ENSG00000146833	TRIM4
25	cg27219362	<NA>	25	ENSG00000120440	TTLL2
26	cg17499241	MEX3A	26	ENSG00000231574	RP11-91K9.1
27	cg01351041	SLC25A36	27	ENSG00000182095	TNRC18
28	cg27227804	CLEC16A	28	ENSG00000134389	CFHR5
29	cg02462253	MAPK10	29	ENSG00000164944	KIAA1429
30	cg17015522	C1QTNF1	30	ENSG00000257139	RP11-320P7.2
31	cg10518481	PTPRS	31	ENSG00000130829	DUSP9
32	cg05384255	<NA>	32	ENSG00000265150	RN7SL2
33	cg12358524	LRRC34	33	ENSG00000168310	IRF2
34	cg20923085	PCDH9	34	ENSG00000167780	SOAT2
35	cg06428055	ELF4	35	ENSG00000205835	GMNC
36	cg12333737	POM121L12	36	ENSG00000227471	AKR1B15
37	cg20218460	LRRFIP1	37	ENSG00000201185	RNA5SP202
38	cg21417204	SMAD4	38	ENSG00000185168	LINC00482
39	cg03853593	SOX30	39	ENSG00000008300	CELSR3
40	cg00756172	LOC340094	40	ENSG00000163884	KLF15
41	cg13909612	PTPRN2	41	ENSG00000169629	RGPD8
42	cg03103218	MACROD1	42	ENSG00000169855	ROBO1
43	cg25692928	CLSTN2	43	ENSG00000196754	S100A2
44	cg19966212	CD74	44	ENSG00000095932	C19orf77
45	cg14517323	<NA>	45	ENSG00000179930	ZNF648
46	cg23140050	<NA>	46	ENSG00000236698	EIF1AXP1

47	cg07791578	KAAG1	47	ENSG00000105825	TFPI2
48	cg27451920	COL6A3	48	ENSG00000163125	RPRD2
49	cg22110922	<NA>	49	ENSG00000043355	ZIC2
50	cg12441957	<NA>	50	ENSG00000177294	FBXO39
51	cg11188837	H2BFWT	51	ENSG00000168925	CTRB1
52	cg25316193	<NA>	52	ENSG00000165905	GYLTL1B
53	cg18756179	FLJ26850	53	ENSG00000116128	BCL9
54	cg12050054	C14orf80	54	ENSG00000272405	RP11-284F21.10
55	cg01485938	UNC5A	55	ENSG00000176563	CNTD1
56	cg14032033	RGS7	56	ENSG00000145826	LECT2
57	cg06621425	PTPRN2	57	ENSG00000226284	ARPC3P1
58	cg06051619	DIP2C	58	ENSG00000260518	BMS1P8
59	cg12504098	<NA>	59	ENSG00000178980	SEPW1
60	cg02747950	RAB8B	60	ENSG00000248596	RP11-844P9.2
61	cg20931907	CXorf67	61	ENSG00000136715	SAP130
62	cg13246744	<NA>	62	ENSG00000258486	RN7SL1
63	cg00322623	<NA>	63	ENSG00000139428	MMAB
64	cg10206440	CLCNKA	64	ENSG00000137076	TLN1
65	cg20129213	RIMS2	65	ENSG00000207005	RNU1-2
66	cg20762313	TMED3	66	ENSG00000141570	CBX8
67	cg26797124	<NA>	67	ENSG00000177599	ZNF491
68	cg16795307	STARD13	68	ENSG00000119042	SATB2
69	cg13891181	<NA>	69	ENSG00000135638	EMX1
70	cg19006211	DPP10	70	ENSG00000107959	PITRM1
71	cg03720897	<NA>	71	ENSG00000215217	C5orf49
72	cg17325866	<NA>	72	ENSG00000119326	CTNNAL1
73	cg26181743	PYCR1	73	ENSG00000248587	GDNF-AS1
74	cg03807917	MIR520G	74	ENSG00000155666	KDM8
75	cg26267483	SGCD	75	ENSG00000108830	RND2
76	cg02832697	TRIM2	76	ENSG00000259001	RPPH1
77	cg01297721	HAPLN4	77	ENSG00000113303	BTNL8
78	cg20934215	PDZRN3	78	ENSG00000148773	MKI67
79	cg25178683	LGALS3BP	79	ENSG00000258938	RP11-317N8.5
80	cg16602369	ANKRD33B	80	ENSG00000272523	LINC01023
81	cg25740250	PTPRN2	81	ENSG00000253696	KBTBD11-OT1
82	cg18417772	<NA>	82	ENSG00000177182	CLVS1
83	cg04527202	CDC14C	83	ENSG00000204778	RP11-15J10.1
84	cg21263567	<NA>	84	ENSG00000184363	PKP3
85	cg16991316	<NA>	85	ENSG00000160691	SHC1
86	cg11225357	<NA>	86	ENSG00000068305	MEF2A
87	cg00785170	PHLDA3	87	ENSG00000010292	NCAPD2
88	cg27519145	FAM78B	88	ENSG00000263316	RP11-530N7.3
89	cg00541683	TINAGL1	89	ENSG00000222011	FAM185A
90	cg05639937	DCLK1	90	ENSG00000047410	TPR
91	cg09394681	<NA>	91	ENSG00000250305	KIAA1456
92	cg13403271	PTPRN2	92	ENSG00000119913	TECTB
93	cg09142578	AATK	93	ENSG00000186376	ZNF75D
94	cg07316217	GABRB3	94	ENSG00000178977	LINC00324
95	cg24804172	<NA>	95	ENSG00000106125	FAM188B

96	cg00120157	<NA>	96	ENSG00000100207	TCF20
97	cg10482224	PTPN14	97	ENSG00000197362	ZNF786
98	cg18579809	<NA>	98	ENSG00000105088	OLFM2
99	cg06888271	<NA>	99	ENSG00000227063	RPL41P1
100	cg13016237	DPP6	100	ENSG00000136720	HS6ST1

Supplemental Table 4

Top 100 Genes - Methylation-Data			Top 100 Genes - RNA-Seq-Data		
1	cg04773745	<NA>	1	ENSG00000138778	CENPE
2	cg04341210	C13orf36	2	ENSG00000270728	RP4-657E11.10
3	cg10916429	ZNF609	3	ENSG00000152760	TCTEX1D1
4	cg05766510	PTPRN2	4	ENSG00000183054	RGPD6
5	cg18620300	FBXL7	5	ENSG00000189334	S100A14
6	cg02514000	WBSCR17	6	ENSG00000232956	SNHG15
7	cg04227077	<NA>	7	ENSG00000107807	TLX1
8	cg05765580	NOS2	8	ENSG00000237978	RP11-385J1.2
9	cg27430369	<NA>	9	ENSG00000204832	ST8SIA6-AS1
10	cg04515534	RADIL	10	ENSG00000107187	LHX3
11	cg00949578	C18orf62	11	ENSG00000232233	RP11-573D15.2
12	cg08611472	LOC285696	12	ENSG00000124564	SLC17A3
13	cg17836612	LGALS3BP	13	ENSG00000126838	PZP
14	cg06720232	SDK1	14	ENSG00000009694	TENM1
15	cg12680822	NCRNA00029	15	ENSG00000196367	TRRAP
16	cg26150533	FABP4	16	ENSG00000214022	REPIN1
17	cg10173787	GPR35	17	ENSG00000265735	RN7SL5P
18	cg04350355	CACNG2	18	ENSG00000074276	CDHR2
19	cg09676669	AQP1	19	ENSG00000009954	BAZ1B
20	cg24500428	PTPRN2	20	ENSG00000144136	SLC20A1
21	cg17623116	LRRC3B	21	ENSG00000106031	HOXA13
22	cg15825065	LRRC6	22	ENSG00000128602	SMO
23	cg10257673	PTPRN2	23	ENSG00000166900	STX3
24	cg22165480	<NA>	24	ENSG00000146833	TRIM4
25	cg27219362	<NA>	25	ENSG00000120440	TTLL2
26	cg17499241	MEX3A	26	ENSG00000231574	RP11-91K9.1
27	cg01351041	SLC25A36	27	ENSG00000182095	TNRC18
28	cg27227804	CLEC16A	28	ENSG00000134389	CFHR5
29	cg02462253	MAPK10	29	ENSG00000164944	KIAA1429
30	cg17015522	C1QTNF1	30	ENSG00000257139	RP11-320P7.2
31	cg10518481	PTPRS	31	ENSG00000130829	DUSP9
32	cg05384255	<NA>	32	ENSG00000265150	RN7SL2
33	cg12358524	LRRC34	33	ENSG00000168310	IRF2
34	cg20923085	PCDH9	34	ENSG00000167780	SOAT2
35	cg06428055	ELF4	35	ENSG00000205835	GMNC
36	cg12333737	POM121L12	36	ENSG00000227471	AKR1B15
37	cg20218460	LRRFIP1	37	ENSG00000201185	RNA5SP202
38	cg21417204	SMAD4	38	ENSG00000185168	LINC00482
39	cg03853593	SOX30	39	ENSG00000008300	CELSR3
40	cg00756172	LOC340094	40	ENSG00000163884	KLF15
41	cg13909612	PTPRN2	41	ENSG00000169629	RGPD8
42	cg03103218	MACROD1	42	ENSG00000169855	ROBO1
43	cg25692928	CLSTN2	43	ENSG00000196754	S100A2
44	cg19966212	CD74	44	ENSG00000095932	C19orf77
45	cg14517323	<NA>	45	ENSG00000179930	ZNF648
46	cg23140050	<NA>	46	ENSG00000236698	EIF1AXP1

47	cg07791578	KAAG1	47	ENSG00000105825	TFPI2
48	cg27451920	COL6A3	48	ENSG00000163125	RPRD2
49	cg22110922	<NA>	49	ENSG00000043355	ZIC2
50	cg12441957	<NA>	50	ENSG00000177294	FBXO39
51	cg11188837	H2BFWT	51	ENSG00000168925	CTRB1
52	cg25316193	<NA>	52	ENSG00000165905	GYLTL1B
53	cg18756179	FLJ26850	53	ENSG00000116128	BCL9
54	cg12050054	C14orf80	54	ENSG00000272405	RP11-284F21.10
55	cg01485938	UNC5A	55	ENSG00000176563	CNTD1
56	cg14032033	RGS7	56	ENSG00000145826	LECT2
57	cg06621425	PTPRN2	57	ENSG00000226284	ARPC3P1
58	cg06051619	DIP2C	58	ENSG00000260518	BMS1P8
59	cg12504098	<NA>	59	ENSG00000178980	SEPW1
60	cg02747950	RAB8B	60	ENSG00000248596	RP11-844P9.2
61	cg20931907	CXorf67	61	ENSG00000136715	SAP130
62	cg13246744	<NA>	62	ENSG00000258486	RN7SL1
63	cg00322623	<NA>	63	ENSG00000139428	MMAB
64	cg10206440	CLCNKA	64	ENSG00000137076	TLN1
65	cg20129213	RIMS2	65	ENSG00000207005	RNU1-2
66	cg20762313	TMED3	66	ENSG00000141570	CBX8
67	cg26797124	<NA>	67	ENSG00000177599	ZNF491
68	cg16795307	STARD13	68	ENSG00000119042	SATB2
69	cg13891181	<NA>	69	ENSG00000135638	EMX1
70	cg19006211	DPP10	70	ENSG00000107959	PITRM1
71	cg03720897	<NA>	71	ENSG00000215217	C5orf49
72	cg17325866	<NA>	72	ENSG00000119326	CTNNAL1
73	cg26181743	PYCR1	73	ENSG00000248587	GDNF-AS1
74	cg03807917	MIR520G	74	ENSG00000155666	KDM8
75	cg26267483	SGCD	75	ENSG00000108830	RND2
76	cg02832697	TRIM2	76	ENSG00000259001	RPPH1
77	cg01297721	HAPLN4	77	ENSG00000113303	BTNL8
78	cg20934215	PDZRN3	78	ENSG00000148773	MKI67
79	cg25178683	LGALS3BP	79	ENSG00000258938	RP11-317N8.5
80	cg16602369	ANKRD33B	80	ENSG00000272523	LINC01023
81	cg25740250	PTPRN2	81	ENSG00000253696	KBTBD11-OT1
82	cg18417772	<NA>	82	ENSG00000177182	CLVS1
83	cg04527202	CDC14C	83	ENSG00000204778	RP11-15J10.1
84	cg21263567	<NA>	84	ENSG00000184363	PKP3
85	cg16991316	<NA>	85	ENSG00000160691	SHC1
86	cg11225357	<NA>	86	ENSG00000068305	MEF2A
87	cg00785170	PHLDA3	87	ENSG00000010292	NCAPD2
88	cg27519145	FAM78B	88	ENSG00000263316	RP11-530N7.3
89	cg00541683	TINAGL1	89	ENSG00000222011	FAM185A
90	cg05639937	DCLK1	90	ENSG00000047410	TPR
91	cg09394681	<NA>	91	ENSG00000250305	KIAA1456
92	cg13403271	PTPRN2	92	ENSG00000119913	TECTB
93	cg09142578	AATK	93	ENSG00000186376	ZNF75D
94	cg07316217	GABRB3	94	ENSG00000178977	LINC00324
95	cg24804172	<NA>	95	ENSG00000106125	FAM188B

96	cg00120157	<NA>	96	ENSG00000100207	TCF20
97	cg10482224	PTPN14	97	ENSG00000197362	ZNF786
98	cg18579809	<NA>	98	ENSG00000105088	OLFM2
99	cg06888271	<NA>	99	ENSG00000227063	RPL41P1
100	cg13016237	DPP6	100	ENSG00000136720	HS6ST1

Supplemental Table 5

Panel	Gene_ Symbol	Lesion	Probe_ID	M-value	p-value	adj.p-value	beta-value difference to NL	Chromosome	UCSC_RefGene_ Group	Relation_to_UCSC_CpG_Island
1	ANO10	LGDN	cg11035303	2,25	0,001400969	0,042313245	0,21	3	Body	
		HGDN	cg11035303	2,20	0,001442059	0,021524631	0,21	3	Body	
		eHCC	cg11035303	2,13	0,001992212	0,012922805	0,20	3	Body	
		pHCC	cg11035303	2,30	0,001162462	0,026053992	0,20	3	Body	
1	BMP8A	LGDN	cg11763509	1,23	1,08373E-05	0,003147873	0,20	1	Body	Island
		HGDN	cg11763509	1,45	4,8908E-08	4,82654E-05	0,23	1	Body	Island
		HGDN	cg02575697	2,05	0,004561701	0,042855262	0,25	1	TSS200	Island
		eHCC	cg25139493	1,29	0,001648639	0,011481088	0,21	1	1stExon	Island
		eHCC	cg11763509	1,46	3,42655E-07	0,000115899	0,23	1	Body	Island
		eHCC	cg08748615	2,10	0,006911376	0,029213458	0,28	1	TSS200	Island
		eHCC	cg15947940	3,01	0,001619369	0,011353715	0,32	1	TSS200	Island
		eHCC	cg02575697	3,01	0,000638407	0,006465884	0,39	1	TSS200	Island
		pHCC	cg11763509	1,40	3,2305E-06	0,001076919	0,22	1	Body	Island
1	CAMTA1	LGDN	cg03571764	-1,64	0,001272387	0,040229801	-0,23	1	Body	Island
		HGDN	cg08640609	1,67	0,004537857	0,042711116	0,21	1	Body	Island
		eHCC	cg12138124	-2,46	3,29437E-05	0,001256476	-0,28	1	Body	
		eHCC	cg07008478	-1,63	0,000855058	0,007693041	-0,25	1	Body	S_Shore
		eHCC	cg21942218	-3,20	0,000286391	0,004055616	-0,24	1	Body	Island
		eHCC	cg03571764	-1,72	0,000278676	0,003994987	-0,24	1	Body	Island
		eHCC	cg23021268	-1,55	0,003666873	0,019151776	-0,23	1	Body	
		eHCC	cg17081408	-1,83	0,001496498	0,010815639	-0,23	1	Body	
		eHCC	cg00911446	-1,91	0,000344462	0,004509207	-0,22	1	Body	

		eHCC	cg25196088	-1,43	0,001309575	0,009952399	-0,22	1	Body	
		eHCC	cg06800235	-1,36	0,000279404	0,003999493	-0,22	1	Body	
		eHCC	cg06082897	-1,57	0,000963949	0,008253847	-0,21	1	Body	S_Shore
		eHCC	cg04210471	-1,59	0,004911013	0,023224209	-0,21	1	Body	Island
		eHCC	cg26161885	-1,66	0,000272859	0,003949708	-0,21	1	Body	
		eHCC	cg17143900	-1,34	0,005124651	0,023891622	-0,21	1	Body	
		eHCC	cg10536786	-1,25	0,001130559	0,009085098	-0,21	1	Body	
		eHCC	cg24579970	-1,21	0,00176546	0,011985081	-0,20	1	Body	S_Shore
		eHCC	cg00783553	1,37	0,005594416	0,025319897	0,22	1	Body	S_Shore
		eHCC	cg22488970	1,62	0,010419843	0,038724737	0,23	1	Body	N_Shore
		eHCC	cg21144493	1,76	1,01642E-05	0,000670116	0,29	1	Body	
		eHCC	cg08640609	2,62	0,000359256	0,004613904	0,36	1	Body	Island
		pHCC	cg12138124	-2,71	5,33231E-05	0,004966698	-0,32	1	Body	
		pHCC	cg00808305	-1,40	0,001385864	0,028577018	-0,23	1	Body	
		pHCC	cg06800235	-1,39	0,000808749	0,02161448	-0,22	1	Body	
		pHCC	cg25763306	-1,55	0,003301719	0,044418039	-0,21	1	Body	S_Shore
		pHCC	cg12661316	-1,43	0,000756895	0,020875328	-0,21	1	Body	
		pHCC	cg21583016	-1,25	0,003911524	0,048481596	-0,20	1	Body	
1	FBRSL1	LGDN	cg08818195	-1,38	2,03787E-05	0,004411797	-0,22	12	TSS1500	N_Shore
		HGDN	cg08818195	-1,32	2,0708E-06	0,000368036	-0,21	12	TSS1500	N_Shore
		eHCC	cg18450555	2,04	0,00229889	0,014172797	0,20	12	TSS1500	Island
		eHCC	cg16719582	1,85	6,89348E-05	0,001862659	0,27	12	Body	
		eHCC	cg00370303	2,28	0,002787085	0,016037777	0,30	12	TSS1500	Island
		eHCC	cg23890800	2,40	0,001807458	0,012162088	0,31	12	TSS1500	Island
		pHCC	cg08818195	-1,23	7,46139E-05	0,00598998	-0,20	12	TSS1500	N_Shore
1	GALK2	LGDN	cg00756450	1,35	1,7713E-08	0,000228386	0,20	15	Body	
		HGDN	cg00756450	1,35	4,36882E-10	6,30125E-06	0,20	15	Body	
		eHCC	cg00756450	1,77	7,13323E-11	8,65566E-06	0,25	15	Body	
		pHCC	cg00756450	1,67	1,06812E-09	8,64058E-05	0,24	15	Body	

1	HMBS	LGDN	cg20929545	1,33	3,56028E-06	0,001800117	0,21	11	TSS1500	S_Shelf
		HGDN	cg20929545	1,31	5,8742E-07	0,000171134	0,21	11	TSS1500	S_Shelf
		eHCC	cg20929545	1,64	9,55531E-08	0,000062667	0,25	11	TSS1500	S_Shelf
		pHCC	cg20929545	1,44	1,54236E-06	0,000764673	0,22	11	TSS1500	S_Shelf
1	MAP4	LGDN	cg16509829	-1,37	1,2804E-05	0,003423929	-0,21	3	5'UTR	Island
		HGDN	cg16509829	-1,33	7,33205E-06	0,000785773	-0,20	3	5'UTR	Island
		eHCC	cg16509829	-1,48	3,23268E-06	0,00036566	-0,22	3	5'UTR	Island
		pHCC	cg16509829	-1,35	1,64411E-05	0,002547039	-0,20	3	5'UTR	Island
1	NSD1	LGDN	cg18121224	1,53	2,1862E-05	0,004589217	0,20	5	TSS1500	Island
		LGDN	cg19731612	1,73	1,9278E-05	0,004300077	0,22	5	TSS1500	Island
		HGDN	cg18121224	1,54	9,38425E-07	0,000229232	0,20	5	TSS1500	Island
		HGDN	cg19731612	1,69	1,62325E-06	0,000319755	0,22	5	TSS1500	Island
		HGDN	cg08369368	1,67	0,000997114	0,017168919	0,23	5	TSS200	Island
		eHCC	cg18016826	1,76	0,000408844	0,004974456	0,22	5	TSS200	Island
		eHCC	cg18121224	2,00	2,02075E-07	8,98113E-05	0,24	5	TSS1500	Island
		eHCC	cg19731612	2,18	3,10701E-07	0,000109997	0,27	5	TSS1500	Island
		eHCC	cg08369368	2,59	7,19393E-05	0,001906875	0,38	5	TSS200	Island
		pHCC	cg18121224	1,55	2,13084E-05	0,0029449	0,20	5	TSS1500	Island
		pHCC	cg19731612	1,61	0,00004665	0,004619989	0,21	5	TSS1500	Island
1	PIAS1	LGDN	cg05105016	1,23	1,15585E-07	0,000390356	0,21	15	Body	
		HGDN	cg05105016	1,22	3,95718E-09	1,41969E-05	0,21	15	Body	
		eHCC	cg05105016	1,57	7,80955E-10	1,18765E-05	0,26	15	Body	
		pHCC	cg05105016	1,66	2,43725E-09	9,35193E-05	0,27	15	Body	
1	PLOD3	LGDN	cg16390570	-1,55	4,20407E-06	0,00197153	-0,25	7	1stExon	Island
		HGDN	cg16390570	-1,32	7,97387E-06	0,000827613	-0,22	7	1stExon	Island
		eHCC	cg16390570	-1,34	1,13889E-05	0,000715301	-0,22	7	1stExon	Island

		eHCC	cg18437077	-1,29	1,10252E-07	6,72273E-05	-0,20	7	1stExon	Island
		pHCC	cg16390570	-1,41	1,30962E-05	0,002250628	-0,23	7	1stExon	Island
1	PPP2R5C	LGDN	cg09990596	-1,28	4,45282E-05	0,006709743	-0,21	14	TSS200	Island
		LGDN	cg15321108	-1,22	1,23203E-05	0,003355745	-0,20	14	TSS200	Island
		HGDN	cg09990596	-1,28	7,15987E-06	0,000773813	-0,22	14	TSS200	Island
		HGDN	cg15321108	-1,28	1,00638E-06	0,000238393	-0,21	14	TSS200	Island
		eHCC	cg08163906	-1,51	5,62682E-06	0,00049052	-0,24	14	TSS1500	Island
		eHCC	cg15321108	-1,32	1,79373E-06	0,000269222	-0,22	14	TSS200	Island
		eHCC	cg09990596	-1,26	2,16154E-05	0,000995773	-0,21	14	TSS200	Island
		pHCC	cg27022326	-1,52	0,000244565	0,011486812	-0,25	14	3'UTR	N_Shore
1	PTPRC	LGDN	cg26399994	-1,82	8,92912E-05	0,009753206	-0,22	1	Body	
		HGDN	cg22073152	-1,30	0,002335102	0,028816727	-0,22	1	Body	
		HGDN	cg04214459	-1,25	0,000698215	0,013820458	-0,21	1	Body	
		eHCC	cg22073152	-2,08	0,000162627	0,002957778	-0,34	1	Body	
		eHCC	cg25132230	-1,63	0,001898857	0,012539625	-0,27	1	5'UTR	
		eHCC	cg26399994	-1,83	2,53383E-05	0,001087514	-0,22	1	Body	
		eHCC	cg04214459	-1,21	0,003153217	0,017366658	-0,20	1	Body	
		pHCC	cg25132230	-1,93	0,00158306	0,030591941	-0,32	1	5'UTR	
		pHCC	cg22073152	-1,84	0,001655583	0,031346453	-0,30	1	Body	
		pHCC	cg04214459	-1,42	0,002846064	0,0412013	-0,23	1	Body	
		pHCC	cg26399994	-1,80	0,000135529	0,008284838	-0,22	1	Body	
1	RWDD1	LGDN	cg23280258	1,40	1,24299E-05	0,003378011	0,21	6	Body	S_Shelf
		HGDN	cg23280258	1,41	5,78259E-07	0,000169794	0,21	6	Body	S_Shelf
		eHCC	cg23280258	1,84	1,0504E-07	6,53631E-05	0,26	6	Body	S_Shelf
		pHCC	cg23280258	1,77	8,8973E-07	0,000577338	0,25	6	Body	S_Shelf
1	SAMD11	LGDN	cg13856810	1,24	0,000236207	0,016280636	0,20	1	Body	S_Shore
		LGDN	cg05527507	1,67	0,000157173	0,013155911	0,27	1	5'UTR	Island

		LGDN	cg14324200	2,01	3,62211E-05	0,005983022	0,31	1	5'UTR	Island
		HGDN	cg13904806	2,08	1,06986E-06	0,000248078	0,20	1	Body	N_Shore
		HGDN	cg13856810	1,40	3,4156E-06	0,000494286	0,23	1	Body	S_Shore
		HGDN	cg02439789	1,56	6,78215E-06	0,000748576	0,25	1	Body	Island
		HGDN	cg24362661	1,52	1,15868E-05	0,001050211	0,25	1	Body	Island
		HGDN	cg06531475	1,61	1,42775E-05	0,001198738	0,26	1	Body	Island
		HGDN	cg05527507	1,67	1,05649E-05	0,000990265	0,27	1	5'UTR	Island
		HGDN	cg14324200	1,79	8,94928E-06	0,000896167	0,28	1	5'UTR	Island
		HGDN	cg13546858	1,84	3,52397E-06	0,000503809	0,29	1	Body	Island
		HGDN	cg02663945	1,82	0,003639257	0,037475353	0,29	1	Body	Island
		eHCC	cg13904806	2,18	3,93301E-06	0,000403672	0,21	1	Body	N_Shore
		eHCC	cg03269716	1,46	4,67217E-06	0,00044236	0,23	1	Body	N_Shore
		eHCC	cg24362661	1,44	5,73347E-05	0,001692634	0,24	1	Body	Island
		eHCC	cg13856810	1,55	6,42576E-06	0,000525064	0,25	1	Body	S_Shore
		eHCC	cg13546858	1,69	3,91963E-05	0,001374563	0,27	1	Body	Island
		eHCC	cg02439789	1,74	6,81567E-06	0,000542672	0,27	1	Body	Island
		eHCC	cg06531475	1,64	5,03767E-05	0,001577203	0,27	1	Body	Island
		eHCC	cg02663945	1,89	0,003134839	0,017305399	0,29	1	Body	Island
		eHCC	cg05527507	1,80	1,91151E-05	0,000933015	0,29	1	5'UTR	Island
		eHCC	cg14324200	2,01	9,3645E-06	0,000645658	0,32	1	5'UTR	Island
		pHCC	cg13904806	2,11	2,86451E-05	0,003499123	0,20	1	Body	N_Shore
		pHCC	cg13856810	1,32	0,000165803	0,009278836	0,21	1	Body	S_Shore
		pHCC	cg24362661	1,33	0,000346405	0,013853106	0,22	1	Body	Island
		pHCC	cg02439789	1,51	0,000114182	0,007524875	0,24	1	Body	Island
		pHCC	cg13546858	1,84	6,48495E-05	0,005561143	0,29	1	Body	Island
		pHCC	cg02663945	2,00	0,002516631	0,038758082	0,31	1	Body	Island
1	SATB2	LGDN	cg20785796	1,34	0,000104345	0,010597593	0,21	2	TSS1500	Island
		HGDN	cg20785796	1,46	4,92183E-06	0,000616767	0,23	2	TSS1500	Island
		eHCC	cg23994043	1,39	0,002854286	0,016282203	0,22	2	TSS1500	Island
		eHCC	cg20731529	1,63	0,001843123	0,012310591	0,26	2	Body	

		eHCC	cg03163783	1,59	0,00113929	0,009128821	0,26	2	TSS1500	Island
		eHCC	cg20785796	1,87	8,08703E-07	0,00017989	0,28	2	TSS1500	Island
		pHCC	cg20785796	1,63	1,48928E-05	0,00242	0,24	2	TSS1500	Island
1	SPG11	LGDN	cg12228919	-1,79	5,09051E-05	0,007254209	-0,24	15	TSS200	Island
		HGDN	cg12228919	-1,81	1,07164E-05	0,000999696	-0,23	15	TSS200	Island
		eHCC	cg12228919	-1,71	3,83926E-05	0,001357519	-0,22	15	TSS200	Island
		pHCC	cg12228919	-1,66	0,0001184	0,007673647	-0,22	15	TSS200	Island
1	STX1A	LGDN	cg01804343	-1,33	3,4312E-05	0,005792713	-0,22	7	TSS200	Island
		HGDN	cg01804343	-1,50	1,34666E-06	0,000283078	-0,25	7	TSS200	Island
		eHCC	cg02610600	1,49	2,71268E-05	0,001127177	0,23	7	Body	Island
		eHCC	cg01804343	-1,73	5,91379E-07	0,000152033	-0,28	7	TSS200	Island
		eHCC	cg27469719	-1,49	1,42012E-06	0,000238837	-0,23	7	TSS200	Island
		pHCC	cg01804343	-1,33	3,64297E-05	0,00400669	-0,22	7	TSS200	Island
1	USP34	LGDN	cg01145124	2,01	3,97126E-08	0,00024712	0,21	2	Body	
		HGDN	cg01145124	1,98	1,18219E-09	9,40658E-06	0,21	2	Body	
		eHCC	cg01145124	2,38	8,16631E-10	1,20112E-05	0,23	2	Body	
		pHCC	cg01145124	2,45	4,09543E-09	9,46573E-05	0,24	2	Body	
1	WDR82	LGDN	cg24007312	-1,33	0,000865119	0,032915715	-0,22	3	TSS200	Island
		HGDN	cg24007312	-1,53	0,00010857	0,004333213	-0,25	3	TSS200	Island
		HGDN	cg11442381	-1,39	1,17748E-05	0,001059934	-0,21	3	TSS200	Island
		eHCC	cg24007312	-1,83	2,55538E-05	0,001092108	-0,29	3	TSS200	Island
		eHCC	cg12661343	-1,33	5,03101E-07	0,000140593	-0,22	3	TSS200	Island
		eHCC	cg11442381	-1,44	1,8899E-05	0,000926286	-0,21	3	TSS200	Island
		pHCC	cg24007312	-1,67	0,000115413	0,007576322	-0,27	3	TSS200	Island
1	ZIC5	LGDN	cg17930361	2,48	0,000702243	0,029581909	0,35	13	Body	Island
		HGDN	cg17930361	1,66	0,002020005	0,026379631	0,22	13	Body	Island

		eHCC	cg11077516	1,24	0,00177198	0,012012285	0,20	13	Body	Island
		eHCC	cg10679688	1,46	0,000569742	0,00603541	0,22	13	TSS1500	S_Shore
		eHCC	cg03313945	1,59	0,000309423	0,004238703	0,23	13	Body	Island
		eHCC	cg20985450	2,57	0,000392496	0,004856565	0,39	13	Body	Island
		eHCC	cg17930361	2,80	6,93423E-05	0,001868431	0,41	13	Body	Island
		pHCC	cg00529958	1,49	0,000767592	0,021041187	0,23	13	TSS200	Island
1	ZNF212	LGDN	cg05476998	1,38	3,19165E-06	0,001734751	0,21	7	Body	S_Shelf
		HGDN	cg05476998	1,45	5,2967E-08	4,92503E-05	0,22	7	Body	S_Shelf
		eHCC	cg05476998	1,81	2,11249E-08	3,42868E-05	0,26	7	Body	S_Shelf
		pHCC	cg05476998	1,82	1,26735E-07	0,000245382	0,26	7	Body	S_Shelf
1	LHX3	LGDN	cg08967938	1,87	0,000967927	0,034864845	0,27	9	Body	Island
		HGDN	cg13658899	1,24	0,000811133	0,015155141	0,21	9	Body	Island
		eHCC	cg14362758	1,55	0,002136962	0,01352007	0,23	9	Body	Island
		pHCC	cg14362758	1,75	0,002625355	0,03954662	0,26	9	Body	Island
		LGDN	cg00554413	1,41	0,000131729	0,011957301	0,22	1	1stExon	Island
1	TACSTD2	HGDN	cg24851854	1,31	1,26168E-05	0,001108588	0,22	1	1stExon	Island
		eHCC	cg24851854	1,53	1,09385E-05	0,00069877	0,26	1	1stExon	Island
		eHCC	cg00667789	1,77	1,97317E-05	0,000946646	0,29	1	1stExon	Island
		pHCC	cg16080552	1,33	0,003686894	0,046971267	0,22	1	TSS200	Island
1	MSX1	LGDN	cg04100843	1,31	0,000836267	0,032350259	0,20	4	TSS1500	N_Shore
		LGDN	cg21689228	1,44	0,001348574	0,041472402	0,22	4	TSS1500	N_Shore
		LGDN	cg03199651	1,70	0,001729766	0,047349555	0,26	4	Body	N_Shore
		HGDN	cg15092343	1,97	4,23898E-05	0,002384926	0,31	4	TSS1500	Island
		eHCC	cg03335246	1,23	0,010623138	0,039249466	0,20	4	TSS1500	N_Shore
		eHCC	cg10266211	1,30	0,000365637	0,004660462	0,22	4	TSS1500	Island
		eHCC	cg20588069	1,37	0,005798135	0,025953559	0,22	4	TSS1500	N_Shore
		eHCC	cg15092343	2,40	1,70495E-05	0,000879044	0,38	4	TSS1500	Island

		pHCC	cg15092343	1,98	0,000414525	0,015226151	0,32	4	TSS1500	Island
2	ATP11A	HGDN	cg08893811	-1,51	0,00232779	0,028763055	-0,23	13	Body	S_Shore
		eHCC	cg08893811	-1,60	0,00317993	0,017462558	-0,26	13	Body	S_Shore
		pHCC	cg25142327	1,93	0,000561165	0,017837661	0,21	13	Body	S_Shore
2	B3GNT9	HGDN	cg06279276	1,45	0,002218368	0,027905445	0,24	16	Body	Island
		eHCC	cg05333146	2,08	0,000156905	0,002904838	0,30	16	Body	Island
		eHCC	cg06279276	2,09	0,000386277	0,00480812	0,34	16	Body	Island
		pHCC	cg06279276	1,92	0,002558498	0,03906448	0,31	16	Body	Island
2	BLOC1S1	HGDN	cg12926596	-1,66	3,89484E-07	0,000136691	-0,24	12	Body	S_Shore
		eHCC	cg12926596	-1,66	1,34152E-06	0,000232516	-0,23	12	Body	S_Shore
		pHCC	cg12926596	-1,49	1,70273E-05	0,00260137	-0,21	12	Body	S_Shore
2	CCDC40	HGDN	cg08109808	1,27	1,15986E-06	0,00025931	0,21	17	Body	N_Shelf
		eHCC	cg08109808	1,59	2,20587E-07	9,31013E-05	0,26	17	Body	N_Shelf
		pHCC	cg08109808	1,55	1,05991E-06	0,000632485	0,25	17	Body	N_Shelf
2	CCR5	HGDN	cg22984586	-2,90	0,00422845	0,040971134	-0,34	3	TSS200	
		eHCC	cg22984586	-2,62	0,008684306	0,034110837	-0,32	3	TSS200	
		pHCC	cg00803692	-1,85	0,000388181	0,014733496	-0,23	3	Body	N_Shelf
2	DDX51	HGDN	cg22672078	-1,39	1,60451E-07	8,52062E-05	-0,23	12	Body	Island
		eHCC	cg22672078	-1,37	7,08938E-07	0,00016728	-0,23	12	Body	Island
		pHCC	cg22672078	-1,21	1,13823E-05	0,002084772	-0,20	12	Body	Island
2	DNAH17	HGDN	cg09687005	-1,40	6,14144E-05	0,003017083	-0,20	17	Body	
		eHCC	cg09687005	-1,82	1,29107E-05	0,000760409	-0,27	17	Body	
		eHCC	cg10217661	-1,93	0,000171966	0,003050257	-0,26	17	Body	Island
		eHCC	cg21103227	-1,96	9,22428E-05	0,002180164	-0,25	17	Body	

		eHCC	cg09705784	-1,71	0,000186965	0,003191535	-0,25	17	Body	
		eHCC	cg01341643	-2,05	7,67345E-05	0,001976056	-0,25	17	Body	Island
		eHCC	cg09577144	-1,65	6,6252E-05	0,001820993	-0,24	17	TSS200	
		eHCC	cg10332979	-1,74	6,82902E-05	0,00185215	-0,24	17	Body	
		eHCC	cg20690714	-2,16	0,000149222	0,002828448	-0,24	17	Body	Island
		eHCC	cg10375710	-1,48	0,000366226	0,004665259	-0,24	17	Body	S_Shelf
		eHCC	cg15618347	-1,81	0,000562421	0,005989096	-0,24	17	Body	
		eHCC	cg25691430	-1,56	0,0007636	0,007192077	-0,23	17	Body	Island
		eHCC	cg11803990	-1,91	0,000283434	0,004031729	-0,23	17	Body	
		eHCC	cg20723425	-1,83	0,001933141	0,01267745	-0,22	17	Body	
		eHCC	cg14927663	-1,98	9,06649E-05	0,002161082	-0,22	17	Body	
		eHCC	cg16678718	-1,94	0,00084096	0,007615679	-0,22	17	Body	N_Shore
		eHCC	cg00461299	-1,56	0,001989067	0,012908616	-0,22	17	Body	S_Shore
		eHCC	cg05361750	-1,67	0,000698823	0,006824991	-0,21	17	Body	
		eHCC	cg00235657	-1,81	0,000670599	0,006664404	-0,21	17	Body	
		eHCC	cg12071008	-1,20	1,01136E-05	0,000668985	-0,20	17	Body	S_Shelf
		eHCC	cg00249503	-1,39	0,000182287	0,003146066	-0,20	17	Body	
		eHCC	cg25399573	-1,91	0,000330609	0,004405026	-0,20	17	Body	S_Shelf
		eHCC	cg25730791	-1,82	0,000144292	0,002776676	-0,20	17	Body	S_Shore
		eHCC	cg17514088	-1,27	0,000189978	0,00321883	-0,20	17	Body	S_Shelf
		pHCC	cg09687005	-1,59	0,000185106	0,009873074	-0,23	17	Body	
2	HLA-DPB2	HGDN	cg11786476	-1,30	9,13045E-05	0,003882438	-0,21	6	Body	
		eHCC	cg11786476	-1,32	0,000129282	0,002616638	-0,21	6	Body	
		pHCC	cg15019001	-1,38	0,002070823	0,035173009	-0,21	6	Body	
		pHCC	cg11786476	-1,31	0,000260193	0,011893961	-0,21	6	Body	
2	KCNK7	HGDN	cg01178624	1,70	2,98276E-07	0,000118668	0,22	11	3'UTR	Island
		eHCC	cg13654525	1,66	7,55637E-06	0,000573029	0,23	11	3'UTR	
		eHCC	cg01178624	1,94	2,38825E-07	9,66868E-05	0,24	11	3'UTR	Island
		pHCC	cg13654525	1,59	5,27809E-05	0,004936093	0,23	11	3'UTR	

		pHCC	cg01178624	1,85	1,97909E-06	0,000851462	0,24	11	3'UTR	Island
2	LITAF	HGDN	cg08767044	-1,75	0,000122039	0,004655068	-0,27	16	5'UTR	N_Shore
		eHCC	cg08767044	-1,75	0,000324005	0,004352332	-0,27	16	5'UTR	N_Shore
		eHCC	cg07994696	-1,36	9,61918E-07	0,000195514	-0,21	16	5'UTR	Island
		pHCC	cg04359558	1,47	0,001119953	0,025569993	0,24	16		
		pHCC	cg08767044	-1,86	0,000525378	0,01721716	-0,29	16	5'UTR	N_Shore
2	NID1	HGDN	cg26837399	1,91	0,000169018	0,005720422	0,28	1	Body	
		eHCC	cg18765906	1,65	0,000435298	0,005152185	0,26	1	Body	
		eHCC	cg26837399	2,51	5,20315E-05	0,001606117	0,34	1	Body	
		pHCC	cg26837399	2,01	0,001536239	0,030121025	0,28	1	Body	
2	PDE4DIP	HGDN	cg19084726	1,56	1,23612E-05	0,001092258	0,23	1	Body	Island
		eHCC	cg15743907	1,28	0,000193912	0,0032557	0,20	1	Body	
		eHCC	cg19084726	2,14	9,39715E-07	0,000193256	0,33	1	Body	Island
		pHCC	cg19084726	1,53	0,000137731	0,008364683	0,22	1	Body	Island
2	PURA	HGDN	cg21778810	-1,39	2,46686E-05	0,001708783	-0,20	5	TSS200	Island
		eHCC	cg21778810	-1,56	1,56117E-05	0,000839329	-0,23	5	TSS200	Island
		pHCC	cg21778810	-1,33	0,000206234	0,0104749	-0,20	5	TSS200	Island
2	SCAMP1	HGDN	cg03056766	-1,40	2,67704E-05	0,001797422	-0,21	5	TSS200	Island
		eHCC	cg03056766	-1,76	4,96589E-06	0,000457026	-0,25	5	TSS200	Island
		pHCC	cg03056766	-1,60	3,66992E-05	0,004025442	-0,23	5	TSS200	Island
2	SMARCC1	HGDN	cg19134770	1,34	1,74423E-06	0,000332913	0,20	3	Body	
		eHCC	cg19134770	1,54	1,20461E-06	0,000220373	0,22	3	Body	
		pHCC	cg19134770	1,49	7,21492E-06	0,001641008	0,21	3	Body	
2	STRN4	HGDN	cg12254611	-1,36	1,84822E-06	0,000345427	-0,23	19	Body	Island

		eHCC	cg12254611	-1,51	1,06453E-06	0,000206487	-0,25	19	Body	Island
		pHCC	cg12254611	-1,45	4,50614E-06	0,001282109	-0,24	19	Body	Island
2	THSD7A	HGDN	cg24676244	1,32	0,000493582	0,011099535	0,21	7	TSS1500	
		eHCC	cg01797590	1,35	0,003258392	0,017732131	0,22	7	TSS200	
		eHCC	cg09557034	1,52	0,000351578	0,004559064	0,24	7	TSS1500	
		eHCC	cg15090509	1,62	0,002053542	0,01318321	0,25	7	TSS1500	
		eHCC	cg24676244	1,88	4,04352E-05	0,001395682	0,30	7	TSS1500	
		pHCC	cg09557034	1,42	0,001686719	0,03163698	0,22	7	TSS1500	
		pHCC	cg24676244	1,61	0,00051808	0,017128325	0,25	7	TSS1500	
		pHCC	cg01797590	1,57	0,002863191	0,04133531	0,25	7	TSS200	
2	UGT2B15	HGDN	cg09189601	1,49	0,003971787	0,03949362	0,22	4	Body	
		eHCC	cg04016992	1,64	2,44741E-07	9,7931E-05	0,25	4	TSS1500	
		eHCC	cg09189601	1,95	0,000765252	0,007201589	0,26	4	Body	
		pHCC	cg04016992	1,48	2,87402E-06	0,001018849	0,22	4	TSS1500	
		pHCC	cg22161115	1,48	0,000906906	0,022917989	0,22	4	1stExon	
		pHCC	cg09189601	1,79	0,002657726	0,039809857	0,26	4	Body	
2	ZNF540	HGDN	cg03975694	1,60	1,19877E-05	0,001072227	0,24	19	5'UTR	S_Shelf
		eHCC	cg03975694	1,95	7,45205E-06	0,00056898	0,30	19	5'UTR	S_Shelf
		pHCC	cg03975694	1,31	0,00146315	0,029389607	0,21	19	5'UTR	S_Shelf
2	ZNF876P	HGDN	cg11836119	1,97	9,34657E-07	0,00022915	0,29	4	Body	Island
		eHCC	cg20296343	1,25	0,002028226	0,01307653	0,21	4	Body	Island
		eHCC	cg12547166	1,40	0,000573854	0,006061744	0,23	4	Body	Island
		eHCC	cg18005867	1,58	0,00023191	0,003604759	0,25	4	TSS200	N_Shore
		eHCC	cg23063647	1,71	0,000115482	0,002463327	0,26	4	TSS200	N_Shore
		eHCC	cg11836119	2,48	3,41294E-07	0,00011561	0,38	4	Body	Island
		pHCC	cg11836119	1,71	0,000117359	0,00764703	0,25	4	Body	Island

2	TMC4	HGDN	cg19488620	1,56	0,000578165	0,012265588	0,25	19	Body	Island
		eHCC	cg19488620	1,85	0,00038676	0,004812381	0,30	19	Body	Island
		pHCC	cg22710065	1,61	0,004008046	0,049097195	0,25	19	Body	Island
2	LTBP2	HGDN	cg17335494	1,33	2,95183E-06	0,00045394	0,20	14	Body	Island
		eHCC	cg17335494	1,71	9,7008E-07	0,000195616	0,24	14	Body	Island
		pHCC	cg17335494	1,63	9,31953E-06	0,001869959	0,24	14	Body	Island
2	HSPE1	HGDN	cg04657470	-1,82	0,00398068	0,039544539	-0,24	2	1stExon	Island
		eHCC	cg04657470	-1,77	0,005671351	0,025560944	-0,22	2	1stExon	Island
		pHCC	cg04657470	-1,95	0,003694816	0,047015393	-0,24	2	1stExon	Island
2	DLG5	HGDN	cg05445097	1,67	6,67974E-09	1,7064E-05	0,21	10	Body	
		eHCC	cg05445097	2,05	2,55651E-09	1,56739E-05	0,24	10	Body	
		eHCC	cg23818888	1,33	0,000224927	0,003545275	0,22	10	Body	S_Shore
		eHCC	cg27586487	1,48	0,000466056	0,005359283	0,24	10	Body	S_Shore
		pHCC	cg05445097	2,12	1,03822E-08	0,000111982	0,24	10	Body	
3	ALDH4A1	eHCC	cg12461099	1,311	0,000199472	0,00330651	0,215	1	TSS1500	
		pHCC	cg22390041	1,252	0,002907524	0,041666014	0,209	1	Body	N_Shelf
		pHCC	cg12461099	1,277	0,00091188	0,022979088	0,214	1	TSS1500	
3	ATG4B	eHCC	cg08969328	1,512	2,43749E-07	9,77949E-05	0,221	2	Body	N_Shelf
		pHCC	cg08969328	1,362	3,86783E-06	0,001194488	0,203	2	Body	N_Shelf
3	ATP6V0A1	eHCC	cg07408552	-1,591	0,001281942	0,02742878	-0,212	17	5'UTR	S_Shore
		pHCC	cg07408552	-1,591	0,001281942	0,02742878	-0,212	17	5'UTR	S_Shore
3	ATP6V1C1	eHCC	cg03506193	-1,801	0,000104466	0,002333175	-0,230	8	5'UTR	S_Shore

		pHCC	cg03506193	-1,530	0,001025482	0,024401386	-0,203	8	5'UTR	S_Shore
3	C1QTNF4	eHCC	cg05537653	1,685	5,2212E-06	0,000470519	0,232	11	Body	Island
		eHCC	cg17282004	1,676	0,000105627	0,002347565	0,270	11	5'UTR	N_Shore
		pHCC	cg18356785	1,546	0,00039042	0,01477456	0,224	11	Body	Island
		pHCC	cg05537653	2,027	2,75848E-06	0,00099236	0,264	11	Body	Island
3	C9orf3	eHCC	cg13853813	1,452	0,000110657	0,002407528	0,203	9	Body	N_Shelf
		eHCC	cg14375632	1,579	0,00017766	0,003106196	0,221	9	Body	N_Shelf
		pHCC	cg13853813	1,358	0,000772395	0,021106496	0,203	9	Body	N_Shelf
		pHCC	cg14582550	1,313	0,001540008	0,030163251	0,216	9	Body	
		pHCC	cg14375632	1,557	0,000684581	0,019814655	0,218	9	Body	N_Shelf
3	CCDC57	eHCC	cg12879038	1,475	3,08029E-05	0,001212628	0,218	17	Body	S_Shelf
		eHCC	cg22142205	1,561	3,40991E-06	0,000375385	0,239	17	Body	
		pHCC	cg22142205	1,339	4,60311E-05	0,004580184	0,202	17	Body	
3	CCDC84	eHCC	cg27211899	1,460	7,64443E-07	0,000174033	0,239	11	Body	S_Shelf
		pHCC	cg27211899	1,484	2,90065E-06	0,001024245	0,243	11	Body	S_Shelf
3	CHERP	eHCC	cg07688052	1,963	0,000193902	0,00325567	0,235	19	Body	S_Shore
		pHCC	cg07688052	1,977	0,00052654	0,017226223	0,236	19	Body	S_Shore
3	CHST11	eHCC	cg11739675	1,630	0,008780547	0,034372311	0,214	12	Body	Island
		eHCC	cg22260952	1,778	0,002707995	0,015736824	0,244	12	Body	S_Shore
		eHCC	cg17844339	-2,548	0,000680007	0,006717223	-0,236	12	Body	Island
		eHCC	cg07696842	-1,292	0,000379752	0,004759914	-0,214	12	Body	
		eHCC	cg12529671	-	0,002815763	0,016142203	-0,209	12	Body	S_Shore

				1,669						
		pHCC	cg01964337	1,281	0,001199658	0,026492301	0,206	12	Body	
		pHCC	cg07696842	-1,403	0,000652404	0,019299821	-0,227	12	Body	
		pHCC	cg16861964	-1,356	0,000308331	0,013052779	-0,227	12	Body	
		pHCC	cg22827210	-1,310	0,000187928	0,009964452	-0,220	12	Body	
3	CLEC14A	eHCC	cg16404157	1,378	0,008322495	0,033120954	0,221	14	1stExon	Island
		eHCC	cg05057720	1,801	0,000327148	0,004377465	0,274	14	1stExon	Island
		pHCC	cg05057720	1,655	0,00163418	0,031111332	0,247	14	1stExon	Island
3	CMYA5	eHCC	cg10257870	1,312	0,000730051	0,00700232	0,212	5	TSS200	
		eHCC	cg09481121	1,384	0,000230309	0,003590465	0,222	5	TSS1500	
		eHCC	cg03546977	1,413	9,60515E-05	0,002228094	0,228	5	TSS200	
		pHCC	cg11438310	1,219	0,000875076	0,022496643	0,202	5	TSS1500	
		pHCC	cg09481121	1,407	0,000593429	0,018400473	0,229	5	TSS1500	
		pHCC	cg00611789	1,395	0,003783493	0,047602404	0,231	5	TSS1500	
		pHCC	cg10257870	1,414	0,000952755	0,023470697	0,235	5	TSS200	
3	CNKSR1	eHCC	cg17330765	1,378	0,001125212	0,025635624	0,214	1	TSS1500	
		pHCC	cg17330765	1,378	0,001125212	0,025635624	0,214	1	TSS1500	
3	CRHBP	eHCC	cg01071966	1,381	0,000729558	0,006998839	0,227	5	1stExon	N_Shore
		pHCC	cg05884167	2,191	0,000227405	0,011034262	0,209	5	Body	S_Shelf
3	CRYBB3	eHCC	cg19288514	1,609	1,67785E-08	3,16982E-05	0,266	22	TSS1500	
		pHCC	cg19288514	1,499	2,37709E-07	0,000328142	0,250	22	TSS1500	
3	CUL3	eHCC	cg01474011	1,560	0,001798314	0,012124535	0,215	2	Body	

		pHCC	cg25502818	1,260	0,00276786	0,040613403	0,212	2	Body	
3	DNASE1L2	eHCC	cg06235653	1,330	0,003322905	0,017966376	0,210	16	Body	Island
		eHCC	cg00249383	1,412	0,000227655	0,003568218	0,228	16	Body	Island
		pHCC	cg00249383	1,286	0,00173383	0,032096978	0,209	16	Body	Island
		pHCC	cg06235653	1,596	0,00210258	0,035423104	0,256	16	Body	Island
3	DNHD1	eHCC	cg10383568	1,500	2,51893E-05	0,001084113	0,249	11	Body	N_Shelf
		pHCC	cg10383568	1,297	0,000442103	0,015777083	0,218	11	Body	N_Shelf
3	EGFLAM	eHCC	cg25344265	-1,796	0,000173692	0,003066918	-0,269	5	Body	
		eHCC	cg18855621	-1,923	7,58721E-06	0,000574267	-0,241	5	Body	
		eHCC	cg08264481	-1,610	6,30507E-06	0,000520237	-0,232	5	Body	
		eHCC	cg11475323	-1,906	7,30788E-05	0,001921857	-0,205	5	Body	S_Shelf
		pHCC	cg25625968	1,250	0,001287529	0,027495665	0,201	5	3'UTR	
3	ELF1	eHCC	cg18456803	-1,514	0,000796076	0,007373515	-0,253	13	TSS200	
		pHCC	cg18456803	-1,559	0,00183015	0,033032031	-0,242	13	TSS200	
3	EXOC2	eHCC	cg04789318	1,427	5,31979E-08	4,95599E-05	0,216	6	5'UTR	N_Shelf
		pHCC	cg04789318	1,402	4,21574E-07	0,000397163	0,211	6	5'UTR	N_Shelf
3	FAM20B	eHCC	cg24997888	1,509	0,000260012	0,003847161	0,227	1	3'UTR	
		pHCC	cg24997888	1,610	0,000529955	0,017284303	0,243	1	3'UTR	

3	FGFR4	eHCC	cg12982374	1,408	0,00032384	0,004351191	0,211	5	Body	S_Shelf
		eHCC	cg17386911	1,617	0,000907565	0,007963218	0,223	5	Body	S_Shelf
		pHCC	cg12982374	1,289	0,002217601	0,036337706	0,201	5	Body	S_Shelf
3	FNDC1	eHCC	cg09107912	1,687	0,000231206	0,003597966	0,243	6	TSS1500	Island
		eHCC	cg00157796	1,567	0,000243301	0,003702274	0,257	6	TSS200	Island
		pHCC	cg07739841	- 1,542	0,001018914	0,02432998	-0,243	6	Body	S_Shelf
3	GAS7	eHCC	cg26999423	- 1,929	0,000418194	0,005037802	-0,298	17	Body	
		eHCC	cg12091339	- 1,922	0,000632805	0,006428996	-0,230	17	Body	
		eHCC	cg07049421	- 1,479	0,001028094	0,008569715	-0,226	17	Body	
		eHCC	cg02605292	- 1,670	0,000670632	0,006664488	-0,201	17	Body	
		pHCC	cg26999423	- 1,844	0,002011096	0,03465132	-0,288	17	Body	
		pHCC	cg06130714	- 1,369	0,000397627	0,01491624	-0,223	17	Body	
3	GLTSCR1	eHCC	cg22461472	1,621	8,72305E-05	0,002116745	0,215	19	Body	S_Shelf
		pHCC	cg22461472	1,529	0,000577861	0,018119573	0,211	19	Body	S_Shelf
3	HELZ	eHCC	cg18432877	1,498	0,000393989	0,004866668	0,215	17	Body	
		pHCC	cg18432877	1,791	0,000286473	0,012544729	0,239	17	Body	
3	HIPK1	eHCC	cg17588904	1,330	4,52477E-07	0,000133055	0,218	1	Body	
		pHCC	cg17588904	1,402	1,11097E-06	0,00064397	0,223	1	Body	
3	HIST1H3H	eHCC	cg01330280	-	0,001917359	0,012611879	-0,259	6	TSS1500	N_Shore

				1,640						
		pHCC	cg01330280	-1,959	0,001474903	0,029509664	-0,321	6	TSS1500	N_Shore
3	HK3	eHCC	cg19960778	-1,970	4,63067E-05	0,001504119	-0,253	5	Body	S_Shore
		eHCC	cg11093640	-1,740	0,00019669	0,003278867	-0,235	5	3'UTR	S_Shelf
		eHCC	cg06485139	-1,614	0,000826268	0,007540737	-0,232	5	Body	N_Shore
		eHCC	cg04875020	-1,426	0,000132017	0,002646224	-0,215	5	Body	N_Shore
		pHCC	cg04875020	-1,528	0,000241237	0,011384661	-0,231	5	Body	N_Shore
3	ITGA11	eHCC	cg25699851	-1,984	8,93371E-05	0,00214507	-0,272	15	Body	
		eHCC	cg26217827	2,600	0,014278532	0,048152421	0,308	15	3'UTR	
		pHCC	cg08872353	-1,552	0,000233928	0,011203012	-0,244	15	Body	N_Shelf
		pHCC	cg24213777	-1,231	0,000419666	0,015334923	-0,203	15	Body	
3	ITGAM	eHCC	cg02256631	1,316	0,006135867	0,026958651	0,205	16	Body	Island
		pHCC	cg22490695	-1,251	0,001683579	0,031611329	-0,207	16	TSS200	
3	ITGB3	eHCC	cg03460756	1,879	9,40275E-05	0,002201982	0,294	17	Body	
		pHCC	cg03460756	1,540	0,001842825	0,033141581	0,250	17	Body	
3	KAT2A	eHCC	cg16550651	1,846	5,26457E-05	0,001615827	0,249	17	Body	N_Shore
		pHCC	cg16550651	1,690	0,000494374	0,016744925	0,250	17	Body	N_Shore

3	KCNS2	eHCC	cg08706670	1,440	0,006555951	0,028188336	0,234	8	TSS200	Island
		eHCC	cg11964564	1,668	0,002889448	0,016412185	0,250	8	5'UTR	Island
		eHCC	cg14486338	2,317	1,65125E-06	0,000257477	0,363	8	Body	Island
		eHCC	cg14688104	3,040	0,001682393	0,011622988	0,371	8	1stExon	Island
		pHCC	cg14688104	3,469	0,001847787	0,033182257	0,415	8	1stExon	Island
3	KIAA1875	eHCC	cg01423393	1,582	1,71307E-05	0,000880983	0,214	8	Body	Island
		pHCC	cg01423393	1,452	7,2645E-05	0,005914085	0,201	8	Body	Island
3	KLHL5	eHCC	cg08217447	1,690	0,0006228	0,006366254	0,247	4	TSS1500	
		pHCC	cg08217447	1,699	0,001756363	0,032326742	0,254	4	TSS1500	
3	LPP	eHCC	cg04423294	1,339	3,16552E-05	0,001230227	0,209	3	Body	
		pHCC	cg24454374	1,213	0,003067089	0,042809967	0,200	3	5'UTR	
3	LZTS1	eHCC	cg05796178	-2,089	2,50859E-05	0,001081459	-0,268	8	Body	S_Shelf
		pHCC	cg13583414	-1,575	0,000643847	0,019167934	-0,228	8	Body	S_Shelf
			cg05796178	-1,815	0,000349394	0,013922142	-0,223	8	Body	S_Shelf
3	MAST2	eHCC	cg02835462	1,233	0,000386466	0,004810093	0,207	1	Body	
		pHCC	cg02835462	1,361	0,000575537	0,018077614	0,227	1	Body	
3	MATN2	eHCC	cg19987349	1,429	3,71774E-07	0,000120037	0,218	8	Body	
		pHCC	cg19987349	1,433	2,04715E-06	0,000862524	0,214	8	Body	
3	MCM6	eHCC	cg11446240	-1,595	3,51221E-05	0,001299532	-0,226	2	Body	N_Shore

		pHCC	cg11446240	- 1,579	0,000157345	0,00900492	-0,225	2	Body	N_Shore
3	MED14	eHCC	cg05812657	1,608	2,15541E-08	3,44223E-05	0,234	X	Body	N_Shelf
		pHCC	cg05812657	1,687	6,30141E-08	0,000183304	0,240	X	Body	N_Shelf
3	MEF2C	eHCC	cg24124703	- 1,438	0,005506455	0,02505221	-0,217	5	5'UTR	
		pHCC	cg24124703	- 1,861	0,002258426	0,036679761	-0,262	5	5'UTR	
3	MICA	eHCC	cg23826579	1,546	1,30982E-07	7,31567E-05	0,251	6	Body	S_Shelf
		pHCC	cg23826579	1,286	4,33075E-06	0,001248971	0,210	6	Body	S_Shelf
3	MYH14	eHCC	cg11992783	1,724	0,000115076	0,002459796	0,206	19	Body	Island
		pHCC	cg11992783	1,821	0,000215926	0,010733716	0,216	19	Body	Island
3	NAA30	eHCC	cg03318573	1,291	0,000488664	0,005512554	0,209	14	TSS1500	N_Shore
		pHCC	cg03318573	1,320	0,001343153	0,028088478	0,212	14	TSS1500	N_Shore
3	NRP2	eHCC	cg17455088	1,335	0,001262114	0,009722315	0,211	2	Body	S_Shore
		eHCC	cg05348875	1,283	3,85549E-05	0,001361272	0,214	2	Body	
		eHCC	cg10648139	1,809	0,000334581	0,004435961	0,271	2	Body	S_Shore
		eHCC	cg22367989	2,896	2,9864E-05	0,001191996	0,399	2	TSS1500	Island
		pHCC	cg17455088	1,325	0,003927781	0,048570256	0,213	2	Body	S_Shore
3	PPAP2B	eHCC	cg10500503	1,244	0,002311455	0,014220871	0,207	1	Body	
		pHCC	cg22396959	1,366	6,53391E-05	0,005585362	0,214	1	Body	
3	PALM3	eHCC	cg11437328	1,636	0,000103987	0,002327386	0,220	19	TSS1500	S_Shelf
		pHCC	cg11437328	1,471	0,001021355	0,024356157	0,215	19	TSS1500	S_Shelf

3	PAQR6	eHCC	cg24152297	1,526	4,7237E-08	4,66868E-05	0,231	1	TSS1500	S_Shelf
		pHCC	cg24152297	1,614	1,27344E-07	0,000245382	0,240	1	TSS1500	S_Shelf
3	PCNX	eHCC	cg14009504	1,541	9,29995E-07	0,000192231	0,213	14	Body	
		pHCC	cg14009504	1,906	3,95852E-07	0,000388937	0,249	14	Body	
3	PIK3CG	eHCC	cg13214190	-1,668	0,007053349	0,029621361	-0,271	7	TSS200	N_Shelf
		eHCC	cg00604356	-1,636	0,000843102	0,007627393	-0,263	7	5'UTR	N_Shore
		pHCC	cg00510718	-3,046	0,00093089	0,023196776	-0,444	7	5'UTR	N_Shelf
		pHCC	cg13214190	-2,422	0,001360526	0,028282152	-0,365	7	TSS200	N_Shelf
		pHCC	cg15881332	-2,204	0,000849698	0,022174918	-0,331	7	TSS200	N_Shelf
		pHCC	cg08779777	-2,115	0,000618061	0,018803362	-0,314	7	TSS200	N_Shelf
		pHCC	cg00661777	-2,053	0,000411995	0,01518033	-0,307	7	Body	S_Shelf
		pHCC	cg11982525	-1,933	0,000945677	0,023385181	-0,303	7	TSS1500	N_Shelf
		pHCC	cg00604356	-1,588	0,003205808	0,04376952	-0,261	7	5'UTR	N_Shore
3	PLEKHF2	eHCC	cg12930753	1,667	0,000191699	0,003233648	0,263	8	3'UTR	
		pHCC	cg12930753	1,394	0,00291337	0,041710621	0,233	8	3'UTR	
3	PPM1D	eHCC	cg04180177	1,252	2,06493E-06	0,000288918	0,205	17	Body	
		pHCC	cg04180177	1,270	7,28475E-06	0,001650356	0,209	17	Body	

3	PRMT7	eHCC	cg10061770	1,574	0,000132487	0,002651869	0,230	16	Body	
		pHCC	cg10061770	1,419	0,00119487	0,026433786	0,204	16	Body	
3	PRR5	eHCC	cg04607412	1,445	0,000633153	0,006430784	0,210	22	5'UTR	
		pHCC	cg04607412	1,764	0,000376057	0,014484217	0,262	22	5'UTR	
3	PTBP1	eHCC	cg19373090	-1,427	2,20224E-07	9,30733E-05	-0,206	19	TSS200	Island
		pHCC	cg19373090	-1,409	7,89021E-07	0,00054168	-0,204	19	TSS200	Island
3	PTPRJ	eHCC	cg04462547	1,365	4,60958E-07	0,000134134	0,208	11	Body	S_Shelf
		eHCC	cg06298729	1,712	0,000239524	0,003671033	0,211	11	Body	
		pHCC	cg04462547	1,358	1,99131E-06	0,000854347	0,206	11	Body	S_Shelf
3	REEP3	eHCC	cg10300729	1,531	1,98415E-07	8,90064E-05	0,237	10	Body	S_Shelf
		pHCC	cg10300729	1,475	1,27E-06	0,000690733	0,228	10	Body	S_Shelf
3	RGS10	eHCC	cg19653161	1,549	0,002574817	0,01522054	0,226	10	TSS1500	Island
		eHCC	cg17527393	2,662	1,27339E-05	0,000756324	0,260	10	TSS200	Island
		pHCC	cg10200202	-1,539	0,000977178	0,02378008	-0,255	10	Body	
3	RNF220	eHCC	cg24603481	-1,697	0,000641549	0,0064862	-0,214	1	Body	S_Shore
		eHCC	cg01422881	1,406	0,000312242	0,004260469	0,237	1	5'UTR	Island
		pHCC	cg09860921	1,317	0,001947444	0,034084364	0,215	1	Body	N_Shelf
3	SCAND3	eHCC	cg19747271	-1,425	3,52492E-05	0,001301472	-0,225	6	Body	N_Shore
		eHCC	cg22302929	1,695	1,91757E-05	0,000934563	0,258	6	1stExon	N_Shore

		pHCC	cg19747271	-1,494	8,53122E-05	0,006437328	-0,237	6	Body	N_Shore
		pHCC	cg22302929	1,305	0,00097212	0,023718003	0,208	6	1stExon	N_Shore
3	SKI	eHCC	cg12483545	1,672	0,002420813	0,014641106	0,230	1	Body	
		pHCC	cg12580943	1,254	0,002315354	0,037158143	0,210	1	Body	
3	SLC25A27	eHCC	cg19101566	1,936	0,00027258	0,003947906	0,288	6	Body	S_Shelf
		pHCC	cg19101566	2,029	0,000566117	0,017929972	0,291	6	Body	S_Shelf
3	SORBS1	eHCC	cg27111150	1,375	6,52052E-05	0,001804757	0,217	10	Body	
		pHCC	cg27111150	1,255	0,000608058	0,018646515	0,202	10	Body	
3	SPN	eHCC	cg02030929	-1,627	0,000442249	0,00519771	-0,228	16	Body	Island
		pHCC	cg09946623	-1,307	0,000805557	0,02156554	-0,205	16	TSS1500	N_Shore
3	SWAP70	eHCC	cg08213398	1,431	8,23954E-07	0,000181688	0,212	11	Body	
		pHCC	cg08213398	1,389	6,41799E-06	0,001555998	0,206	11	Body	
3	TAF7L	eHCC	cg19386336	1,513	0,000545741	0,005886281	0,210	X	Body	Island
		eHCC	cg01538344	1,525	0,000402553	0,004930357	0,244	X	1stExon	Island
		pHCC	cg01538344	1,332	0,003016047	0,042460241	0,213	X	1stExon	Island
3	TIGD1	eHCC	cg18346402	1,518	3,05397E-07	0,000108993	0,249	2	1stExon	N_Shelf
		pHCC	cg18346402	1,534	1,59034E-06	0,000776591	0,252	2	1stExon	N_Shelf
3	TNS1	eHCC	cg06320380	1,744	4,86194E-05	0,001545514	0,273	2	5'UTR	
		eHCC	cg09548780	2,360	1,21401E-05	0,000736362	0,334	2	5'UTR	
		pHCC	cg12681370	1,368	0,000356473	0,014046256	0,218	2	5'UTR	

3	TSC2	eHCC	cg07730183	1,854	0,0001864	0,003185782	0,212	16	Body	S_Shore
		eHCC	cg06094085	1,503	0,001664462	0,011549222	0,215	16	Body	S_Shore
		eHCC	cg02504384	1,452	0,000417413	0,005032416	0,220	16	Body	N_Shelf
		eHCC	cg02364279	1,929	0,000903404	0,007942003	0,232	16	Body	N_Shore
		eHCC	cg06330323	1,594	0,000949283	0,008178712	0,246	16	Body	N_Shore
		pHCC	cg07730183	1,714	0,001321892	0,027828249	0,208	16	Body	S_Shore
3	TXNDC5	eHCC	cg11401394	1,712	0,000266358	0,003897322	0,255	6	Body	
		pHCC	cg11401394	1,730	0,0008659	0,022381669	0,266	6	Body	
3	UCN	eHCC	cg05113927	1,744	9,60018E-07	0,000195291	0,223	2	TSS200	Island
		eHCC	cg01454215	1,562	0,002163461	0,013625739	0,229	2	3'UTR	Island
		pHCC	cg05113927	1,519	2,51897E-05	0,00325082	0,202	2	TSS200	Island
3	ZDHHC7	eHCC	cg03655147	1,327	6,80724E-06	0,000542317	0,220	16	5'UTR	N_Shelf
		eHCC	cg09735674	-1,240	1,76519E-05	0,00089602	-0,208	16	5'UTR	Island
		pHCC	cg03655147	1,331	1,914E-05	0,002779045	0,215	16	5'UTR	N_Shelf
3	ZMIZ2	eHCC	cg11425656	1,311	2,80346E-05	0,001149676	0,204	7	5'UTR	S_Shelf
		pHCC	cg11425656	1,361	8,29045E-05	0,006348372	0,215	7	5'UTR	S_Shelf
3	ZNF366	eHCC	cg11905892	-2,567	0,002712455	0,015752691	-0,291	5	Body	Island
		eHCC	cg22558265	-2,606	0,000212609	0,003430324	-0,246	5	Body	Island
		eHCC	cg00657287	-1,836	0,000982352	0,008344657	-0,218	5	Body	N_Shore
		pHCC	cg04454664	1,253	0,000262241	0,011943707	0,207	5	Body	

3	EBF3	eHCC	cg22952849	-2,310	3,09135E-05	0,001214837	-0,301	10	Body	N_Shore
		eHCC	cg07506153	-2,606	0,005569163	0,025243439	-0,288	10	Body	N_Shore
		eHCC	cg20800606	-1,834	0,00067282	0,006677521	-0,256	10	Body	Island
		eHCC	cg27454298	-2,091	0,000271399	0,003938483	-0,241	10	Body	Island
		eHCC	cg10598433	-2,212	0,000352589	0,004565217	-0,224	10	Body	S_Shore
		eHCC	cg02337436	-2,071	0,000458332	0,005307685	-0,213	10	Body	
		eHCC	cg03128163	-1,594	0,000112163	0,00242401	-0,209	10	Body	N_Shore
		eHCC	cg15991708	-1,586	6,8076E-05	0,0018482	-0,206	10	Body	
		eHCC	cg19582265	-1,455	6,81884E-05	0,001850109	-0,218	10	TSS1500	
		pHCC	cg04043455							
3	FAM189A1	eHCC	cg19263124	-2,078	5,56733E-05	0,00166393	-0,239	15	Body	
		eHCC	cg18512553	-2,042	0,000129207	0,002616178	-0,237	15	Body	
		eHCC	cg12711059	-1,486	9,64515E-05	0,002233551	-0,226	15	3'UTR	
		eHCC	cg08904058	-1,260	0,00010854	0,002382418	-0,212	15	Body	
		eHCC	cg21207450	-1,460	0,000366217	0,004665259	-0,200	15	Body	
		pHCC	cg12711059	-1,510	0,000319038	0,013289653	-0,227	15	3'UTR	

		pHCC	cg25941083	- 1,342	0,000934362	0,023252263	-0,227	15	Body	N_Shore
3	GPR158	eHCC	cg14506260	1,307	0,007127305	0,029833619	0,207	10	Body	
		eHCC	cg24361761	2,232	0,00041694	0,005029219	0,345	10	Body	
		pHCC	cg24361761					10	Body	
3	IRX3	eHCC	cg08279075	1,763	0,001841239	0,012304077	0,271	16	Body	Island
		pHCC	cg05001964	1,328	0,001413256	0,028877383	0,203	16	Body	Island
3	KRTCAP3	eHCC	cg17158414	1,739	0,001462983	0,010658876	0,216	2	1stExon	Island
		eHCC	cg11618577	1,529	0,004597055	0,022223653	0,220	2	Body	Island
		eHCC	cg24768116	1,661	0,001495017	0,010809349	0,236	2	TSS200	Island
		eHCC	cg04845466	1,869	0,000128646	0,002610293	0,246	2	TSS200	Island
		eHCC	cg02592271	1,881	0,004601606	0,022239456	0,247	2	Body	Island
		pHCC	cg17158414	1,773	0,003062764	0,042779192	0,219	2	1stExon	Island
		pHCC	cg04845466	1,893	0,000360845	0,014146618	0,234	2	TSS200	Island
3	TMEM18	eHCC	cg27237671	1,418	0,000653957	0,006562296	0,227	2	Body	N_Shore
		pHCC	cg27237671	1,327	0,003406437	0,045094384	0,213	2	Body	N_Shore
3	WNT7A	eHCC	cg23655615	- 1,817	0,00125122	0,009675403	-0,243	3	Body	Island
		eHCC	cg10460033	- 1,524	0,000259	0,003837685	-0,239	3	Body	N_Shore
		eHCC	cg21224025	- 1,467	0,001549776	0,011053473	-0,214	3	Body	S_Shelf
		pHCC	cg10460033	- 1,350	0,002408034	0,03792216	-0,214	3	Body	N_Shore
3	ZYG11A	eHCC	cg20287790	2,232	1,67669E-06	0,000259292	0,242	1	3'UTR	

		pHCC	cg20287790	1,688	0,000170838	0,009443107	0,217	1	3'UTR	
3	APC2	eHCC	cg19333963	1,974	0,003810961	0,01964788	0,314	19	Body	Island
		eHCC	cg03306486	2,479	0,002036304	0,013109485	0,378	19	Body	Island
		eHCC	cg10808783	2,602	0,000248662	0,003747633	0,385	19	Body	Island
		pHCC	cg10808783							
3	CBFA2T3	eHCC	cg27434245	1,444	0,000527098	0,005763933	0,203	16	1stExon	Island
		eHCC	cg04072301	-2,046	8,47368E-06	0,000612218	-0,306	16	5'UTR	Island
		eHCC	cg02633398	-1,408	0,000986554	0,008367024	-0,223	16	5'UTR	N_Shore
		eHCC	cg03021297	-1,821	0,001129361	0,009079844	-0,215	16	5'UTR	
		pHCC	cg00762678	-1,265	0,000881122	0,022581799	-0,208		5'UTR	N_Shelf
		pHCC	cg04699663	-1,242	0,000962478	0,023596265	-0,203		1stExon	
3	CNOT4	eHCC	cg25871890	1,457	0,000747295	0,007102994	0,234	7	5'UTR	
		pHCC	cg25871890	1,532	0,001348339	0,02815058	0,239	7	5'UTR	
3	COPA	eHCC	cg09866659	1,500	5,83221E-08	5,15072E-05	0,252	1	Body	
		pHCC	cg09866659	1,327	1,35954E-06	0,000711844	0,222	1	Body	
3	CR1L	eHCC	cg06917617	1,306	0,000190517	0,003222883	0,216	1	Body	Island
		pHCC	cg06917617	1,584	0,000117677	0,007654385	0,257	1	Body	Island
3	CREBBP	eHCC	cg03368634	2,023	3,19922E-05	0,001236525	0,296	16	Body	
		pHCC	cg03368634	1,434	0,00266988	0,039905231	0,233	16	Body	

3	CYP1A2	eHCC	cg04968473	1,677	0,0002478	0,00374116	0,250	15	TSS1500	
		pHCC	cg04968473	1,409	0,003353047	0,044745176	0,208	15	TSS1500	
3	HDAC9	eHCC	cg04892643	-1,653	3,80242E-06	0,000396133	-0,271	7	Body	
		eHCC	cg16925459	-1,402	9,45234E-05	0,002208479	-0,227	7	Body	
		eHCC	cg08285151	-1,358	0,001998609	0,012949354	-0,221	7	TSS1500	
		pHCC	cg08285151	-1,717	0,000921164	0,023074082	-0,285	7	TSS1500	
		pHCC	cg04892643	-1,379	0,000119477	0,007713594	-0,225	7	Body	
3	HSF5	eHCC	cg25793387	1,796	0,000116076	0,002470337	0,234	17	Body	
		pHCC	cg25793387	1,888	0,000267711	0,012083983	0,246	17	Body	
3	KREMEN2	eHCC	cg26494929	1,560	0,002733419	0,015836891	0,235	16	Body	Island
			cg05169951	2,182	1,22767E-05	0,000741323	0,279	16	Body	Island
		pHCC	cg05169951	1,621	0,00091564	0,023018862	0,219	16	Body	Island
3	S100A6	eHCC	cg01910639	-1,530	2,45761E-06	0,000316155	-0,255	1	Body	N_Shore
		pHCC	cg01910639	-1,265	9,15466E-05	0,006695385	-0,211	1	Body	N_Shore
3	SIPA1L1	eHCC	cg15378445	1,495	3,64569E-08	4,09609E-05	0,228	14	Body	
		eHCC	cg02058870	1,777	0,000376026	0,004733271	0,280	14	5'UTR	
		pHCC	cg15378445	1,570	1,13855E-07	0,000233189	0,234	14	Body	
3	SNORD6	eHCC	cg03531787	1,449	0,000368829	0,00468219	0,206	11	TSS200	
		eHCC	cg06955958	1,558	0,000306768	0,004216591	0,251	11	TSS200	

		pHCC	cg06955958	1,617	0,000778465	0,021187295	0,264	11	TSS200	
3	STX3	eHCC	cg14333691	1,463	1,93006E-08	3,35383E-05	0,224	11	3'UTR	
		pHCC	cg14333691	1,431	1,58326E-07	0,000267759	0,217	11	3'UTR	
3	THRSP	eHCC	cg03493668	1,398	0,000520835	0,005720482	0,229	11	3'UTR	
		pHCC	cg03493668	1,316	0,002383629	0,03772453	0,215	11	3'UTR	
3	TSPAN10	eHCC	cg18268547	1,471	0,000633127	0,006430646	0,236	17	3'UTR	Island
		pHCC	cg18268547	1,340	0,004026282	0,049224879	0,214	17	3'UTR	Island
3	USP42	eHCC	cg13977660	1,531	9,01962E-07	0,000189354	0,252	7	5'UTR	S_Shelf
		pHCC	cg13977660	1,340	0,004026282	0,049224879	0,214	7	5'UTR	S_Shelf
3	ACTA2	eHCC	cg03755566	1,682	8,13988E-06	0,000598617	0,271	10	Body	
		pHCC	cg03755566	1,474	0,000151188	0,008793536	0,235	10	Body	
3	ARHGAP10	eHCC	cg13248315	1,641	1,00649E-07	6,4364E-05	0,244	10	Body	N_Shelf
		pHCC	cg13248315					10	Body	N_Shelf
3	COL14A1	eHCC	cg26179069	-1,453	1,68032E-05	0,000873201	-0,233	8	Body	
		eHCC	cg05830842	-1,454	7,51908E-05	0,001952561	-0,228	8	Body	
		eHCC	cg10133738	-1,759	0,000216387	0,003464022	-0,207	8	3'UTR	
		pHCC	cg05830842	1,442	2,31963E-06	0,00091091	0,215	8	Body	
3	E4F1	eHCC	cg27038935	-1,773	1,68939E-09	1,32594E-05	-0,224	16	TSS200	Island
		pHCC	cg27038935	-	2,30428E-08	0,000133146	-0,215	16	TSS200	Island

				1,689						
3	FAM129A	eHCC	cg05655671	2,061	0,003705965	0,019287685	0,316	1	1stExon	Island
		pHCC	cg17464436	-1,786	4,424E-05	0,004475372	-0,242	1	Body	
3	HS3ST2	eHCC	cg04718102	-2,204	0,000771279	0,007233639	-0,312	16	Body	Island
		eHCC	cg01555981	-2,320	0,001228153	0,00956831	-0,269	16	Body	Island
		eHCC	cg05970721	-1,845	4,04465E-05	0,001395974	-0,236	16	Body	Island
		eHCC	cg10180297	-1,316	3,64699E-05	0,001322484	-0,220	16	Body	S_Shore
		pHCC	cg10180297	-1,351	0,00012067	0,007763727	-0,228	16	Body	S_Shore
3	KIF26A	eHCC	cg11792470	-2,565	0,000299114	0,004154802	-0,291	14	Body	Island
		eHCC	cg01549570	-1,946	5,88539E-05	0,001713924	-0,286	14	Body	Island
		eHCC	cg00742738	-2,041	0,001506599	0,010860826	-0,264	14	Body	N_Shore
		eHCC	cg19800856	-1,845	4,85261E-05	0,001543559	-0,249	14	Body	Island
		eHCC	cg13250679	-1,648	0,001865206	0,012402283	-0,248	14	3'UTR	S_Shore
		eHCC	cg01933329	-2,655	0,001158278	0,009218869	-0,246	14	Body	Island
		eHCC	cg12468119	-1,642	0,004577351	0,022163995	-0,235	14	Body	Island
		eHCC	cg24706981	-	0,001023731	0,008547955	-0,226	14	Body	N_Shore

				1,889						
		eHCC	cg06760280	-1,568	0,000215225	0,003452445	-0,225	14	Body	N_Shore
		eHCC	cg16520046	-1,715	0,000943398	0,008147366	-0,223	14	Body	S_Shore
		eHCC	cg19003626	-1,949	0,000453261	0,00527236	-0,221	14	Body	N_Shore
		eHCC	cg23892535	-1,565	0,001489474	0,010784306	-0,215	14	Body	Island
		eHCC	cg02922817	-1,643	0,000423466	0,005072836	-0,215	14	Body	N_Shore
		eHCC	cg25131771	-1,267	0,000332767	0,004422609	-0,207	14	Body	Island
		pHCC	cg01549570	-1,416	0,002910726	0,041688509	-0,201	14	Body	Island
3	MRVI1	eHCC	cg17299456	-1,554	0,000557138	0,005955705	-0,213	11	5'UTR	
		pHCC	cg17471425							
3	PLVAP	eHCC	cg15219347	-1,836	0,000311766	0,004256726	-0,287	19	TSS1500	S_Shore
		eHCC	cg01662455	-1,315	0,000413255	0,005005784	-0,221	19	1stExon	Island
		eHCC	cg18302786	-1,517	0,000963315	0,008251243	-0,208	19	TSS200	S_Shore
		eHCC	cg10709246	-1,275	0,001799566	0,012130169	-0,205	19	5'UTR	Island
		eHCC	cg23881613	-2,091	8,5276E-06	0,000614467	-0,200	19	3'UTR	Island
		pHCC	cg10178628	-1,401	0,001873357	0,033413199	-0,218	19	Body	

3	TFIP11	eHCC	cg03019505	1,408	2,63095E-07	0,000101671	0,236	22	Body	S_Shelf	
		pHCC	1,303376649	1,303	3,93172E-06	0,001199461	0,218	22	Body	S_Shelf	
3	THBS2	eHCC	cg02997295	-1,743	0,000910415	0,007979071	-0,275	6	Body	S_Shore	
		eHCC	cg04476508	-1,585	0,000897118	0,007909381	-0,251	6	Body	Island	
		eHCC	cg06119452	-1,560	0,000428559	0,005106589	-0,247	6	Body	N_Shore	
		eHCC	cg01320433	-1,502	0,001466524	0,010673409	-0,243	6	Body	N_Shore	
		eHCC	cg02785555	-1,718	0,000533908	0,005807525	-0,239	6	Body	Island	
		eHCC	cg15216858	-2,318	0,000100322	0,002282748	-0,229	6	Body	Island	
		eHCC	cg25631414	-2,019	0,000508566	0,005642518	-0,225	6	Body	Island	
		eHCC	cg00438284	-1,413	8,99616E-05	0,002153094	-0,207	6	Body	S_Shelf	
		eHCC	cg03091070	-1,388	0,003222734	0,017612259	-0,206	6	Body	N_Shore	
		pHCC	cg01320433	-1,695	0,001646196	0,031252485	-0,271	6	Body	N_Shore	
3	ZFP91	eHCC	cg12027899	1,543	1,19831E-05	0,000731878	0,237	11	3'UTR		
		pHCC	cg12027899	1,287	0,000333865	0,013610629	0,209	11	3'UTR		

Supplemental Table 6

Signature 1	Signature 2	Signature 3
DMGR	DMGR	DMGR
ADAMTS2	ABCG1	ABCA1
ADCK5	ACAN	ACACB
ANO10	ADAL	ACAD8
ARHGAP15	ADAM32	ACOT2
ATG10	APBA2	ACSF2
ATP8B3	ARMS2	ACSL5
BANP	ASTN2	ACSS2
BDNF	ATP11A	ACTA2
BMP8A	B3GNT9	ACTL6B
BMP8B	BCAR3	ADAM2
C16orf87	BLOC1S1	ADAMTS12
C18orf56	BNC2	ADAMTSL2
C2orf65	C10orf26	AFARP1
C2orf82	C10orf53	AKAP1
C8orf85	C1QL4	ALDH1A2
CAMTA1	C21orf57	ALDH4A1
CAPN13	C2orf84	ALPL
CD160	CALB1	AMZ1
CD164L2	CASR	ANK2
CHFR	CCDC40	ANKRD32
CLIP2	CCR5	ANKRD33
CNOT6	CHD5	ANKRD58
CRMP1	CHGA	ANKS1A
CTNND2	CINP	APBA1
DEFA3	CLIC3	APC2
DLEC1	CPLX2	ARHGAP19
DLX6AS	CPT1B	ARHGDIB
EHBP1L1	CTTNBP2	ARSJ
ESPN	CUGBP2	ASAP1
EXOC6	DDX51	ASTN1
FAM49B	DLG5	ATG4B
FBRSL1	DLK1	ATP6V0A1
FBXL7	DNAH10	ATP6V1C1
FOXD3	DNAH17	BDH2
FRAS1	DPY19L2P2	BLVRB
GABRB3	DRD2	BMF
GALK2	DRD5	BUB1B
GALNT9	ECH1	C12orf51
GATA4	EDNRB	C14orf115
GMDS	EFHD2	C14orf21
GPR123	EIF2C3	C15orf42
GRAMD1B	ENTPD8	C16orf70
HCN2	ERC2	C17orf46

HMBS	ETV5	C17orf73
HOXD8	FADS6	C1orf125
IGF1R	FAM90A14	C1orf14
IPO7	FGF19	C1orf150
KCNE1	FLJ26850	C1orf187
KIAA1026	FLJ32063	C1orf189
KIAA1841	FOXE1	C1QTNF4
LHX3	FOXE3	C22orf24
LOC100129354	GABRA4	C2CD3
LOC145783	GBX2	C3orf27
LOC285780	GFRA1	C5orf4
LOC90110	GGN	C6orf174
LRRC43	GJC2	C6orf48
MAP4	GLB1L2	C9orf3
MGAT5B	GPIHBP1	CACNA1C
MIR1910	GRHL2	CACNA1E
MIR496	HBQ1	CACNA2D2
MMP19	HIST1H2BH	CACNA2D3
MSX1	HIST3H2BB	CAMSAP1L1
NCRNA00171	HLA-DPB2	CAND2
NKX6-2	HLA-DQB1	CAPN5
NOTCH4	HNRNPAB	CAPSL
NSD1	HOXA2	CARD16
NTM	HOXA9	CARHSP1
PARD6A	HOXD9	CBFA2T3
PCDHGA4	HSPE1	CBR3
PDPK1	IFNGR1	CCDC109A
PHLDA3	IGSF21	CCDC48
PIAS1	IL17D	CCDC57
PITPNB	INSM2	CCDC84
PKDREJ	JAK3	CCK
PLOD3	JMJD5	CCNJL
PPP1R14D	KARS	CD53
PPP2R5C	KCNH3	CD6
PTPRC	KCNIP1	CDCP1
PTPRS	KCNJ2	CDH11
PUF60	KCNK7	CDK3
PUS7	KLRG2	CHERP
RASL11B	LAMC3	CHRNA3
RNF135	LAPTM4B	CHST11
RUNX3	LITAF	CLDN5
RWDD1	LOC339788	CLEC14A
SAMD11	LOC389033	CLEC4C
SATB2	LOC389458	CLSTN1
SDK1	LOC91316	CLU
SLC25A2	LTBP2	CMTM8
SLC25A41	MBP	CMYA5

SOHLH1	MICB	CNKSR1
SORCS2	MIR129-2	CNOT4
SPG11	MTMR7	CNR2
ST3GAL3	MYO10	COL14A1
ST8SIA3	NID1	COL2A1
STX1A	NPAS4	COPA
TACC2	NR2E1	CORO2B
TACSTD2	NRG3	CPA4
TBX15	NRXN2	CPA6
TLE4	NTF3	CPN1
TNFRSF9	NUBP2	CR1L
TOX2	OBSCN	CREBBP
TP73	OSR2	CRH
TRAPPC9	OTX1	CRHBP
TRIM26	PDE10A	CRISPLD2
TRIM58	PDE4DIP	CRYBB3
TTYH3	PHF21B	CSMD3
ULBP1	PITX2	CUL3
USP34	PLXNB2	CYP19A1
UTF1	POU4F1	CYP1A2
UTS2D	PPP1R14A	CYP2E1
VIPR2	PRDM2	CYTIP
WDR82	PRDM8	DCAF4L2
ZIC5	PURA	DENND1C
ZNF212	RANBP17	DFFB
ZNF41	RAP1GAP2	DGKD
ZSCAN18	REXO1L2P	DMPK
	RGS17	DMRTA2
	RIMS2	DNASE1L2
	SCAMP1	DNHD1
	SEMA6D	DOK2
	SLC2A14	DPP10
	SLC6A7	DPYSL5
	SLC8A1	DSC3
	SMARCC1	DSCAML1
	SNX26	DSTN
	SP110	DVL1
	STAG3	E4F1
	STRN4	EBF3
	SYCE1	EBPL
	SYT16	EDARADD
	TAS2R60	EFNA5
	TCTN3	EFS
	TEAD4	EGFLAM
	TEX101	ELF1
	TFCP2L1	EPHX4
	THSD7A	ERN2

	TMC4	ESRRG
	TPCN1	EXOC2
	TRIM15	FAHD2A
	TRMT12	FAM100A
	TWIST1	FAM113B
	UGT2B15	FAM129A
	VAX1	FAM184B
	WDR8	FAM189A1
	ZBTB10	FAM196B
	ZIC1	FAM19A2
	ZNF154	FAM19A4
	ZNF177	FAM20B
	ZNF331	FAM20C
	ZNF471	FAM83A
	ZNF540	FBP1
	ZNF560	FGF13
	ZNF562	FGF2
	ZNF667	FGFR4
	ZNF876P	FILIP1
		FLNB
		FNDC1
		FOXI1
		FRMD1
		GABRA3
		GABRB1
		GAGE2E
		GALR2
		GAS7
		GCNT3
		GEMIN6
		GFAP
		GFPT2
		GIMAP7
		GJA3
		GLB1L3
		GLI3
		GLRB
		GLTSCR1
		GLTSCR2
		GNAO1
		GNG4
		GNL1
		GPR158
		GPR20
		GPRIN3
		GRAMD3
		GRASP

		GRIN2A<br>GRM2<br>GULP1<br>GZMA<br>HDAC9<br>HECW1<br>HELZ<br>HERC5<br>HIF1AN<br>HIPK1<br>HIST1H3H<br>HK3<br>HMGA2<br>HNMT<br>HOXA4<br>HOXC4<br>HS3ST2<br>HSF5<br>HSPB6<br>IFFO1<br>IFI16<br>IFNG<br>IGLON5<br>IKZF1<br>IL12RB2<br>INPP5D<br>IRX3<br>ITGA11<br>ITGAE<br>ITGAM<br>ITGB3<br>ITPKB<br>ITPRIPL1<br>KAT2A<br>KBTBD11<br>KCNAB2<br>KCNG4<br>KCNJ16<br>KCNK16<br>KCNK9<br>KCNS2<br>KDM5C<br>KIAA0556<br>KIAA0664<br>KIAA0748<br>KIAA1751<br>KIAA1875	

		KIF15	
		KIF26A	
		KLF4	
		KLF5	
		KLHL5	
		KLRG1	
		KREMEN2	
		KRT32	
		KRT5	
		KRT6C	
		KRT71	
		KRTAP12-3	
		KRTAP2-4	
		KRTCAP3	
		KSR2	
		LAIR1	
		LFNG	
		LMBRD1	
		LMO2	
		LOC100133991	
		LOC146880	
		LOC257358	
		LOC285768	
		LOC339524	
		LOC90784	
		LPP	
		LRIG1	
		LRRC10B	
		LRRC36	
		LRRFIP2	
		LRWD1	
		LTA	
		LY75	
		LY9	
		LYNX1	
		LZTS1	
		MALL	
		MANEAL	
		MARCO	
		MASP1	
		MAST2	
		MATN2	
		MCM6	
		MED12L	
		MED14	
		MED24	
		MEF2C	

		MGAT5	
		MGST1	
		MICA	
		MIR485	
		MIR495	
		MKL1	
		MMEL1	
		MON2	
		MRVI1	
		MS4A7	
		MTMR3	
		MTOR	
		MUPCDH	
		MX2	
		MYH14	
		MYPOP	
		NAA30	
		NARG2	
		NFIA	
		NKAIN1	
		NKAPL	
		NPR3	
		NRD1	
		NRN1L	
		NRP2	
		NT5DC1	
		NUDT9	
		NUMBL	
		OLFM1	
		OPA3	
		OR13A1	
		OR4D6	
		OR6K2	
		OTUD7A	
		PABPC4	
		PALM3	
		PAPLN	
		PAQR6	
		PARK2	
		PARK7	
		PARP9	
		PASK	
		PCBP3	
		PCNX	
		PDE11A	
		PDE7B	
		PDLIM5	

		PDZRN4	
		PEG10	
		PEX14	
		PHF14	
		PIAS2	
		PIK3CD	
		PIK3CG	
		PIK3R6	
		PLA2G4F	
		PLAT	
		PLCE1	
		PLDN	
		PLEKHA9	
		PLEKHF2	
		PLEKHG5	
		PLVAP	
		PNPLA6	
		POLR1A	
		POR	
		PPAP2B	
		PPAPDC3	
		PPARGC1A	
		PPM1D	
		PPM1F	
		PRELP	
		PRIMA1	
		PRKCG	
		PRMT7	
		PRPSAP2	
		PRR5	
		PRSS8	
		psiTPTE22	
		PTBP1	
		PTP4A3	
		PTPRJ	
		PTPRK	
		PXDNL	
		QSOX1	
		RAB37	
		RABGAP1L	
		RAD51L1	
		RAET1G	
		RASGRP2	
		RASSF2	
		RBPJ	
		REEP3	
		RGS10	

		RHOC	
		RIMS1	
		RNF122	
		RNF220	
		RORA	
		RPSAP58	
		RTCD1	
		RTL1	
		S100A6	
		SAMD13	
		SCAND3	
		SCG5	
		SCN11A	
		SCN5A	
		SDC2	
		SEMA3C	
		SENP7	
		SFRP1	
		SFRP2	
		SFRS8	
		SFT2D2	
		SH3BGRL2	
		SH3RF3	
		SHANK2	
		SIPA1L1	
		SKI	
		SLA	
		SLC12A9	
		SLC16A1	
		SLC1A6	
		SLC25A27	
		SLC2A5	
		SLC41A1	
		SLC45A1	
		SLC6A19	
		SLC7A10	
		SLC9A3	
		SLCO3A1	
		SNORA2A	
		SNORA54	
		SNORD126	
		SNORD6	
		SORBS1	
		SORBS2	
		SPERT	
		SPN	
		SPNS3	

		SPSB4<br>SREBF1<br>SSR1<br>ST8SIA5<br>STAC2<br>STK10<br>STX3<br>SUSD1<br>SWAP70<br>TAF7L<br>TCL6<br>TESK2<br>TFAP2A<br>TFEC<br>TFIP11<br>THBS2<br>THRSP<br>TIGD1<br>TJP2<br>TMEM178<br>TMEM18<br>TMEM206<br>TNS1<br>TOMM20<br>TPST1<br>TRIM31<br>TRIM59<br>TRIM72<br>TRPC7<br>TRPV6<br>TSC2<br>TSNAX-DISC1<br>TSPAN10<br>TSPY4<br>TTC29<br>TTLL8<br>TXNDC5<br>UBE2E1<br>UCN<br>UGP2<br>UGT1A5<br>USP42<br>UTP11L<br>VARS<br>WDFY4<br>WDR1<br>WFDC1	

		WIPF1
		WNK2
		WNT7A
		WSCD1
		WSCD2
		XKR6
		XPOT
		ZBTB16
		ZBTB20
		ZBTB9
		ZC3H12D
		ZDHHC7
		ZFP91
		ZMAT4
		ZMIZ2
		ZNF148
		ZNF181
		ZNF204P
		ZNF229
		ZNF274
		ZNF366
		ZNF389
		ZNF461
		ZNF582
		ZNF711
		ZNF831
		ZNF853
		ZSWIM4
		ZYG11A

Supplemental Table 7

Panel	Gene_ Symbol	Lesion	Probe_ID	M-value	p-value	adj.p-value	beta-value difference to NL	Chromosome	UCSC_RefGene_ Group	Relation_to_UCSC_CpG_Island
1	ANO10	LGDN	cg11035303	2,25	0,001400969	0,042313245	0,21	3	Body	
		HGDN	cg11035303	2,20	0,001442059	0,021524631	0,21	3	Body	
		eHCC	cg11035303	2,13	0,001992212	0,012922805	0,20	3	Body	
		pHCC	cg11035303	2,30	0,001162462	0,026053992	0,20	3	Body	
1	BMP8A	LGDN	cg11763509	1,23	1,08373E-05	0,003147873	0,20	1	Body	Island
		HGDN	cg11763509	1,45	4,8908E-08	4,82654E-05	0,23	1	Body	Island
		HGDN	cg02575697	2,05	0,004561701	0,042855262	0,25	1	TSS200	Island
		eHCC	cg25139493	1,29	0,001648639	0,011481088	0,21	1	1stExon	Island
		eHCC	cg11763509	1,46	3,42655E-07	0,000115899	0,23	1	Body	Island
		eHCC	cg08748615	2,10	0,006911376	0,029213458	0,28	1	TSS200	Island
		eHCC	cg15947940	3,01	0,001619369	0,011353715	0,32	1	TSS200	Island
		eHCC	cg02575697	3,01	0,000638407	0,006465884	0,39	1	TSS200	Island
		pHCC	cg11763509	1,40	3,2305E-06	0,001076919	0,22	1	Body	Island
1	CAMTA1	LGDN	cg03571764	-1,64	0,001272387	0,040229801	-0,23	1	Body	Island
		HGDN	cg08640609	1,67	0,004537857	0,042711116	0,21	1	Body	Island
		eHCC	cg12138124	-2,46	3,29437E-05	0,001256476	-0,28	1	Body	
		eHCC	cg07008478	-1,63	0,000855058	0,007693041	-0,25	1	Body	S_Shore
		eHCC	cg21942218	-3,20	0,000286391	0,004055616	-0,24	1	Body	Island
		eHCC	cg03571764	-1,72	0,000278676	0,003994987	-0,24	1	Body	Island
		eHCC	cg23021268	-1,55	0,003666873	0,019151776	-0,23	1	Body	
		eHCC	cg17081408	-1,83	0,001496498	0,010815639	-0,23	1	Body	
		eHCC	cg00911446	-1,91	0,000344462	0,004509207	-0,22	1	Body	

		eHCC	cg25196088	-1,43	0,001309575	0,009952399	-0,22	1	Body	
		eHCC	cg06800235	-1,36	0,000279404	0,003999493	-0,22	1	Body	
		eHCC	cg06082897	-1,57	0,000963949	0,008253847	-0,21	1	Body	S_Shore
		eHCC	cg04210471	-1,59	0,004911013	0,023224209	-0,21	1	Body	Island
		eHCC	cg26161885	-1,66	0,000272859	0,003949708	-0,21	1	Body	
		eHCC	cg17143900	-1,34	0,005124651	0,023891622	-0,21	1	Body	
		eHCC	cg10536786	-1,25	0,001130559	0,009085098	-0,21	1	Body	
		eHCC	cg24579970	-1,21	0,00176546	0,011985081	-0,20	1	Body	S_Shore
		eHCC	cg00783553	1,37	0,005594416	0,025319897	0,22	1	Body	S_Shore
		eHCC	cg22488970	1,62	0,010419843	0,038724737	0,23	1	Body	N_Shore
		eHCC	cg21144493	1,76	1,01642E-05	0,000670116	0,29	1	Body	
		eHCC	cg08640609	2,62	0,000359256	0,004613904	0,36	1	Body	Island
		pHCC	cg12138124	-2,71	5,33231E-05	0,004966698	-0,32	1	Body	
		pHCC	cg00808305	-1,40	0,001385864	0,028577018	-0,23	1	Body	
		pHCC	cg06800235	-1,39	0,000808749	0,02161448	-0,22	1	Body	
		pHCC	cg25763306	-1,55	0,003301719	0,044418039	-0,21	1	Body	S_Shore
		pHCC	cg12661316	-1,43	0,000756895	0,020875328	-0,21	1	Body	
		pHCC	cg21583016	-1,25	0,003911524	0,048481596	-0,20	1	Body	
1	FBRSL1	LGDN	cg08818195	-1,38	2,03787E-05	0,004411797	-0,22	12	TSS1500	N_Shore
		HGDN	cg08818195	-1,32	2,0708E-06	0,000368036	-0,21	12	TSS1500	N_Shore
		eHCC	cg18450555	2,04	0,00229889	0,014172797	0,20	12	TSS1500	Island
		eHCC	cg16719582	1,85	6,89348E-05	0,001862659	0,27	12	Body	
		eHCC	cg00370303	2,28	0,002787085	0,016037777	0,30	12	TSS1500	Island
		eHCC	cg23890800	2,40	0,001807458	0,012162088	0,31	12	TSS1500	Island
		pHCC	cg08818195	-1,23	7,46139E-05	0,00598998	-0,20	12	TSS1500	N_Shore
1	GALK2	LGDN	cg00756450	1,35	1,7713E-08	0,000228386	0,20	15	Body	
		HGDN	cg00756450	1,35	4,36882E-10	6,30125E-06	0,20	15	Body	
		eHCC	cg00756450	1,77	7,13323E-11	8,65566E-06	0,25	15	Body	
		pHCC	cg00756450	1,67	1,06812E-09	8,64058E-05	0,24	15	Body	

1	HMBS	LGDN	cg20929545	1,33	3,56028E-06	0,001800117	0,21	11	TSS1500	S_Shelf	
		HGDN	cg20929545	1,31	5,8742E-07	0,000171134	0,21	11	TSS1500	S_Shelf	
		eHCC	cg20929545	1,64	9,55531E-08	0,000062667	0,25	11	TSS1500	S_Shelf	
		pHCC	cg20929545	1,44	1,54236E-06	0,000764673	0,22	11	TSS1500	S_Shelf	
1	MAP4	LGDN	cg16509829	-1,37	1,2804E-05	0,003423929	-0,21	3	5'UTR	Island	
		HGDN	cg16509829	-1,33	7,33205E-06	0,000785773	-0,20	3	5'UTR	Island	
		eHCC	cg16509829	-1,48	3,23268E-06	0,00036566	-0,22	3	5'UTR	Island	
		pHCC	cg16509829	-1,35	1,64411E-05	0,002547039	-0,20	3	5'UTR	Island	
1	NSD1	LGDN	cg18121224	1,53	2,1862E-05	0,004589217	0,20	5	TSS1500	Island	
		LGDN	cg19731612	1,73	1,9278E-05	0,004300077	0,22	5	TSS1500	Island	
		HGDN	cg18121224	1,54	9,38425E-07	0,000229232	0,20	5	TSS1500	Island	
		HGDN	cg19731612	1,69	1,62325E-06	0,000319755	0,22	5	TSS1500	Island	
		HGDN	cg08369368	1,67	0,000997114	0,017168919	0,23	5	TSS200	Island	
		eHCC	cg18016826	1,76	0,000408844	0,004974456	0,22	5	TSS200	Island	
		eHCC	cg18121224	2,00	2,02075E-07	8,98113E-05	0,24	5	TSS1500	Island	
		eHCC	cg19731612	2,18	3,10701E-07	0,000109997	0,27	5	TSS1500	Island	
		eHCC	cg08369368	2,59	7,19393E-05	0,001906875	0,38	5	TSS200	Island	
		pHCC	cg18121224	1,55	2,13084E-05	0,0029449	0,20	5	TSS1500	Island	
		pHCC	cg19731612	1,61	0,00004665	0,004619989	0,21	5	TSS1500	Island	
1	PIAS1	LGDN	cg05105016	1,23	1,15585E-07	0,000390356	0,21	15	Body		
		HGDN	cg05105016	1,22	3,95718E-09	1,41969E-05	0,21	15	Body		
		eHCC	cg05105016	1,57	7,80955E-10	1,18765E-05	0,26	15	Body		
		pHCC	cg05105016	1,66	2,43725E-09	9,35193E-05	0,27	15	Body		
1	PLOD3	LGDN	cg16390570	-1,55	4,20407E-06	0,00197153	-0,25	7	1stExon	Island	
		HGDN	cg16390570	-1,32	7,97387E-06	0,000827613	-0,22	7	1stExon	Island	
		eHCC	cg16390570	-1,34	1,13889E-05	0,000715301	-0,22	7	1stExon	Island	

		eHCC	cg18437077	-1,29	1,10252E-07	6,72273E-05	-0,20	7	1stExon	Island
		pHCC	cg16390570	-1,41	1,30962E-05	0,002250628	-0,23	7	1stExon	Island
1	PPP2R5C	LGDN	cg09990596	-1,28	4,45282E-05	0,006709743	-0,21	14	TSS200	Island
		LGDN	cg15321108	-1,22	1,23203E-05	0,003355745	-0,20	14	TSS200	Island
		HGDN	cg09990596	-1,28	7,15987E-06	0,000773813	-0,22	14	TSS200	Island
		HGDN	cg15321108	-1,28	1,00638E-06	0,000238393	-0,21	14	TSS200	Island
		eHCC	cg08163906	-1,51	5,62682E-06	0,00049052	-0,24	14	TSS1500	Island
		eHCC	cg15321108	-1,32	1,79373E-06	0,000269222	-0,22	14	TSS200	Island
		eHCC	cg09990596	-1,26	2,16154E-05	0,000995773	-0,21	14	TSS200	Island
		pHCC	cg27022326	-1,52	0,000244565	0,011486812	-0,25	14	3'UTR	N_Shore
1	PTPRC	LGDN	cg26399994	-1,82	8,92912E-05	0,009753206	-0,22	1	Body	
		HGDN	cg22073152	-1,30	0,002335102	0,028816727	-0,22	1	Body	
		HGDN	cg04214459	-1,25	0,000698215	0,013820458	-0,21	1	Body	
		eHCC	cg22073152	-2,08	0,000162627	0,002957778	-0,34	1	Body	
		eHCC	cg25132230	-1,63	0,001898857	0,012539625	-0,27	1	5'UTR	
		eHCC	cg26399994	-1,83	2,53383E-05	0,001087514	-0,22	1	Body	
		eHCC	cg04214459	-1,21	0,003153217	0,017366658	-0,20	1	Body	
		pHCC	cg25132230	-1,93	0,00158306	0,030591941	-0,32	1	5'UTR	
		pHCC	cg22073152	-1,84	0,001655583	0,031346453	-0,30	1	Body	
		pHCC	cg04214459	-1,42	0,002846064	0,0412013	-0,23	1	Body	
		pHCC	cg26399994	-1,80	0,000135529	0,008284838	-0,22	1	Body	
1	RWDD1	LGDN	cg23280258	1,40	1,24299E-05	0,003378011	0,21	6	Body	S_Shelf
		HGDN	cg23280258	1,41	5,78259E-07	0,000169794	0,21	6	Body	S_Shelf
		eHCC	cg23280258	1,84	1,0504E-07	6,53631E-05	0,26	6	Body	S_Shelf
		pHCC	cg23280258	1,77	8,8973E-07	0,000577338	0,25	6	Body	S_Shelf
1	SAMD11	LGDN	cg13856810	1,24	0,000236207	0,016280636	0,20	1	Body	S_Shore
		LGDN	cg05527507	1,67	0,000157173	0,013155911	0,27	1	5'UTR	Island

		LGDN	cg14324200	2,01	3,62211E-05	0,005983022	0,31	1	5'UTR	Island
		HGDN	cg13904806	2,08	1,06986E-06	0,000248078	0,20	1	Body	N_Shore
		HGDN	cg13856810	1,40	3,4156E-06	0,000494286	0,23	1	Body	S_Shore
		HGDN	cg02439789	1,56	6,78215E-06	0,000748576	0,25	1	Body	Island
		HGDN	cg24362661	1,52	1,15868E-05	0,001050211	0,25	1	Body	Island
		HGDN	cg06531475	1,61	1,42775E-05	0,001198738	0,26	1	Body	Island
		HGDN	cg05527507	1,67	1,05649E-05	0,000990265	0,27	1	5'UTR	Island
		HGDN	cg14324200	1,79	8,94928E-06	0,000896167	0,28	1	5'UTR	Island
		HGDN	cg13546858	1,84	3,52397E-06	0,000503809	0,29	1	Body	Island
		HGDN	cg02663945	1,82	0,003639257	0,037475353	0,29	1	Body	Island
		eHCC	cg13904806	2,18	3,93301E-06	0,000403672	0,21	1	Body	N_Shore
		eHCC	cg03269716	1,46	4,67217E-06	0,00044236	0,23	1	Body	N_Shore
		eHCC	cg24362661	1,44	5,73347E-05	0,001692634	0,24	1	Body	Island
		eHCC	cg13856810	1,55	6,42576E-06	0,000525064	0,25	1	Body	S_Shore
		eHCC	cg13546858	1,69	3,91963E-05	0,001374563	0,27	1	Body	Island
		eHCC	cg02439789	1,74	6,81567E-06	0,000542672	0,27	1	Body	Island
		eHCC	cg06531475	1,64	5,03767E-05	0,001577203	0,27	1	Body	Island
		eHCC	cg02663945	1,89	0,003134839	0,017305399	0,29	1	Body	Island
		eHCC	cg05527507	1,80	1,91151E-05	0,000933015	0,29	1	5'UTR	Island
		eHCC	cg14324200	2,01	9,3645E-06	0,000645658	0,32	1	5'UTR	Island
		pHCC	cg13904806	2,11	2,86451E-05	0,003499123	0,20	1	Body	N_Shore
		pHCC	cg13856810	1,32	0,000165803	0,009278836	0,21	1	Body	S_Shore
		pHCC	cg24362661	1,33	0,000346405	0,013853106	0,22	1	Body	Island
		pHCC	cg02439789	1,51	0,000114182	0,007524875	0,24	1	Body	Island
		pHCC	cg13546858	1,84	6,48495E-05	0,005561143	0,29	1	Body	Island
		pHCC	cg02663945	2,00	0,002516631	0,038758082	0,31	1	Body	Island
1	SATB2	LGDN	cg20785796	1,34	0,000104345	0,010597593	0,21	2	TSS1500	Island
		HGDN	cg20785796	1,46	4,92183E-06	0,000616767	0,23	2	TSS1500	Island
		eHCC	cg23994043	1,39	0,002854286	0,016282203	0,22	2	TSS1500	Island
		eHCC	cg20731529	1,63	0,001843123	0,012310591	0,26	2	Body	

		eHCC	cg03163783	1,59	0,00113929	0,009128821	0,26	2	TSS1500	Island
		eHCC	cg20785796	1,87	8,08703E-07	0,00017989	0,28	2	TSS1500	Island
		pHCC	cg20785796	1,63	1,48928E-05	0,00242	0,24	2	TSS1500	Island
1	SPG11	LGDN	cg12228919	-1,79	5,09051E-05	0,007254209	-0,24	15	TSS200	Island
		HGDN	cg12228919	-1,81	1,07164E-05	0,000999696	-0,23	15	TSS200	Island
		eHCC	cg12228919	-1,71	3,83926E-05	0,001357519	-0,22	15	TSS200	Island
		pHCC	cg12228919	-1,66	0,0001184	0,007673647	-0,22	15	TSS200	Island
1	STX1A	LGDN	cg01804343	-1,33	3,4312E-05	0,005792713	-0,22	7	TSS200	Island
		HGDN	cg01804343	-1,50	1,34666E-06	0,000283078	-0,25	7	TSS200	Island
		eHCC	cg02610600	1,49	2,71268E-05	0,001127177	0,23	7	Body	Island
		eHCC	cg01804343	-1,73	5,91379E-07	0,000152033	-0,28	7	TSS200	Island
		eHCC	cg27469719	-1,49	1,42012E-06	0,000238837	-0,23	7	TSS200	Island
		pHCC	cg01804343	-1,33	3,64297E-05	0,00400669	-0,22	7	TSS200	Island
1	USP34	LGDN	cg01145124	2,01	3,97126E-08	0,00024712	0,21	2	Body	
		HGDN	cg01145124	1,98	1,18219E-09	9,40658E-06	0,21	2	Body	
		eHCC	cg01145124	2,38	8,16631E-10	1,20112E-05	0,23	2	Body	
		pHCC	cg01145124	2,45	4,09543E-09	9,46573E-05	0,24	2	Body	
1	WDR82	LGDN	cg24007312	-1,33	0,000865119	0,032915715	-0,22	3	TSS200	Island
		HGDN	cg24007312	-1,53	0,00010857	0,004333213	-0,25	3	TSS200	Island
		HGDN	cg11442381	-1,39	1,17748E-05	0,001059934	-0,21	3	TSS200	Island
		eHCC	cg24007312	-1,83	2,55538E-05	0,001092108	-0,29	3	TSS200	Island
		eHCC	cg12661343	-1,33	5,03101E-07	0,000140593	-0,22	3	TSS200	Island
		eHCC	cg11442381	-1,44	1,8899E-05	0,000926286	-0,21	3	TSS200	Island
		pHCC	cg24007312	-1,67	0,000115413	0,007576322	-0,27	3	TSS200	Island
1	ZIC5	LGDN	cg17930361	2,48	0,000702243	0,029581909	0,35	13	Body	Island
		HGDN	cg17930361	1,66	0,002020005	0,026379631	0,22	13	Body	Island

		eHCC	cg11077516	1,24	0,00177198	0,012012285	0,20	13	Body	Island
		eHCC	cg10679688	1,46	0,000569742	0,00603541	0,22	13	TSS1500	S_Shore
		eHCC	cg03313945	1,59	0,000309423	0,004238703	0,23	13	Body	Island
		eHCC	cg20985450	2,57	0,000392496	0,004856565	0,39	13	Body	Island
		eHCC	cg17930361	2,80	6,93423E-05	0,001868431	0,41	13	Body	Island
		pHCC	cg00529958	1,49	0,000767592	0,021041187	0,23	13	TSS200	Island
1	ZNF212	LGDN	cg05476998	1,38	3,19165E-06	0,001734751	0,21	7	Body	S_Shelf
		HGDN	cg05476998	1,45	5,2967E-08	4,92503E-05	0,22	7	Body	S_Shelf
		eHCC	cg05476998	1,81	2,11249E-08	3,42868E-05	0,26	7	Body	S_Shelf
		pHCC	cg05476998	1,82	1,26735E-07	0,000245382	0,26	7	Body	S_Shelf
1	LHX3	LGDN	cg08967938	1,87	0,000967927	0,034864845	0,27	9	Body	Island
		HGDN	cg13658899	1,24	0,000811133	0,015155141	0,21	9	Body	Island
		eHCC	cg14362758	1,55	0,002136962	0,01352007	0,23	9	Body	Island
		pHCC	cg14362758	1,75	0,002625355	0,03954662	0,26	9	Body	Island
		LGDN	cg00554413	1,41	0,000131729	0,011957301	0,22	1	1stExon	Island
1	TACSTD2	HGDN	cg24851854	1,31	1,26168E-05	0,001108588	0,22	1	1stExon	Island
		eHCC	cg24851854	1,53	1,09385E-05	0,00069877	0,26	1	1stExon	Island
		eHCC	cg00667789	1,77	1,97317E-05	0,000946646	0,29	1	1stExon	Island
		pHCC	cg16080552	1,33	0,003686894	0,046971267	0,22	1	TSS200	Island
1	MSX1	LGDN	cg04100843	1,31	0,000836267	0,032350259	0,20	4	TSS1500	N_Shore
		LGDN	cg21689228	1,44	0,001348574	0,041472402	0,22	4	TSS1500	N_Shore
		LGDN	cg03199651	1,70	0,001729766	0,047349555	0,26	4	Body	N_Shore
		HGDN	cg15092343	1,97	4,23898E-05	0,002384926	0,31	4	TSS1500	Island
		eHCC	cg03335246	1,23	0,010623138	0,039249466	0,20	4	TSS1500	N_Shore
		eHCC	cg10266211	1,30	0,000365637	0,004660462	0,22	4	TSS1500	Island
		eHCC	cg20588069	1,37	0,005798135	0,025953559	0,22	4	TSS1500	N_Shore
		eHCC	cg15092343	2,40	1,70495E-05	0,000879044	0,38	4	TSS1500	Island

		pHCC	cg15092343	1,98	0,000414525	0,015226151	0,32	4	TSS1500	Island
2	ATP11A	HGDN	cg08893811	-1,51	0,00232779	0,028763055	-0,23	13	Body	S_Shore
		eHCC	cg08893811	-1,60	0,00317993	0,017462558	-0,26	13	Body	S_Shore
		pHCC	cg25142327	1,93	0,000561165	0,017837661	0,21	13	Body	S_Shore
2	B3GNT9	HGDN	cg06279276	1,45	0,002218368	0,027905445	0,24	16	Body	Island
		eHCC	cg05333146	2,08	0,000156905	0,002904838	0,30	16	Body	Island
		eHCC	cg06279276	2,09	0,000386277	0,00480812	0,34	16	Body	Island
		pHCC	cg06279276	1,92	0,002558498	0,03906448	0,31	16	Body	Island
2	BLOC1S1	HGDN	cg12926596	-1,66	3,89484E-07	0,000136691	-0,24	12	Body	S_Shore
		eHCC	cg12926596	-1,66	1,34152E-06	0,000232516	-0,23	12	Body	S_Shore
		pHCC	cg12926596	-1,49	1,70273E-05	0,00260137	-0,21	12	Body	S_Shore
2	CCDC40	HGDN	cg08109808	1,27	1,15986E-06	0,00025931	0,21	17	Body	N_Shelf
		eHCC	cg08109808	1,59	2,20587E-07	9,31013E-05	0,26	17	Body	N_Shelf
		pHCC	cg08109808	1,55	1,05991E-06	0,000632485	0,25	17	Body	N_Shelf
2	CCR5	HGDN	cg22984586	-2,90	0,00422845	0,040971134	-0,34	3	TSS200	
		eHCC	cg22984586	-2,62	0,008684306	0,034110837	-0,32	3	TSS200	
		pHCC	cg00803692	-1,85	0,000388181	0,014733496	-0,23	3	Body	N_Shelf
2	DDX51	HGDN	cg22672078	-1,39	1,60451E-07	8,52062E-05	-0,23	12	Body	Island
		eHCC	cg22672078	-1,37	7,08938E-07	0,00016728	-0,23	12	Body	Island
		pHCC	cg22672078	-1,21	1,13823E-05	0,002084772	-0,20	12	Body	Island
2	DNAH17	HGDN	cg09687005	-1,40	6,14144E-05	0,003017083	-0,20	17	Body	
		eHCC	cg09687005	-1,82	1,29107E-05	0,000760409	-0,27	17	Body	
		eHCC	cg10217661	-1,93	0,000171966	0,003050257	-0,26	17	Body	Island
		eHCC	cg21103227	-1,96	9,22428E-05	0,002180164	-0,25	17	Body	

		eHCC	cg09705784	-1,71	0,000186965	0,003191535	-0,25	17	Body	
		eHCC	cg01341643	-2,05	7,67345E-05	0,001976056	-0,25	17	Body	Island
		eHCC	cg09577144	-1,65	6,6252E-05	0,001820993	-0,24	17	TSS200	
		eHCC	cg10332979	-1,74	6,82902E-05	0,00185215	-0,24	17	Body	
		eHCC	cg20690714	-2,16	0,000149222	0,002828448	-0,24	17	Body	Island
		eHCC	cg10375710	-1,48	0,000366226	0,004665259	-0,24	17	Body	S_Shelf
		eHCC	cg15618347	-1,81	0,000562421	0,005989096	-0,24	17	Body	
		eHCC	cg25691430	-1,56	0,0007636	0,007192077	-0,23	17	Body	Island
		eHCC	cg11803990	-1,91	0,000283434	0,004031729	-0,23	17	Body	
		eHCC	cg20723425	-1,83	0,001933141	0,01267745	-0,22	17	Body	
		eHCC	cg14927663	-1,98	9,06649E-05	0,002161082	-0,22	17	Body	
		eHCC	cg16678718	-1,94	0,00084096	0,007615679	-0,22	17	Body	N_Shore
		eHCC	cg00461299	-1,56	0,001989067	0,012908616	-0,22	17	Body	S_Shore
		eHCC	cg05361750	-1,67	0,000698823	0,006824991	-0,21	17	Body	
		eHCC	cg00235657	-1,81	0,000670599	0,006664404	-0,21	17	Body	
		eHCC	cg12071008	-1,20	1,01136E-05	0,000668985	-0,20	17	Body	S_Shelf
		eHCC	cg00249503	-1,39	0,000182287	0,003146066	-0,20	17	Body	
		eHCC	cg25399573	-1,91	0,000330609	0,004405026	-0,20	17	Body	S_Shelf
		eHCC	cg25730791	-1,82	0,000144292	0,002776676	-0,20	17	Body	S_Shore
		eHCC	cg17514088	-1,27	0,000189978	0,00321883	-0,20	17	Body	S_Shelf
		pHCC	cg09687005	-1,59	0,000185106	0,009873074	-0,23	17	Body	
2	HLA-DPB2	HGDN	cg11786476	-1,30	9,13045E-05	0,003882438	-0,21	6	Body	
		eHCC	cg11786476	-1,32	0,000129282	0,002616638	-0,21	6	Body	
		pHCC	cg15019001	-1,38	0,002070823	0,035173009	-0,21	6	Body	
		pHCC	cg11786476	-1,31	0,000260193	0,011893961	-0,21	6	Body	
2	KCNK7	HGDN	cg01178624	1,70	2,98276E-07	0,000118668	0,22	11	3'UTR	Island
		eHCC	cg13654525	1,66	7,55637E-06	0,000573029	0,23	11	3'UTR	
		eHCC	cg01178624	1,94	2,38825E-07	9,66868E-05	0,24	11	3'UTR	Island
		pHCC	cg13654525	1,59	5,27809E-05	0,004936093	0,23	11	3'UTR	

		pHCC	cg01178624	1,85	1,97909E-06	0,000851462	0,24	11	3'UTR	Island
2	LITAF	HGDN	cg08767044	-1,75	0,000122039	0,004655068	-0,27	16	5'UTR	N_Shore
		eHCC	cg08767044	-1,75	0,000324005	0,004352332	-0,27	16	5'UTR	N_Shore
		eHCC	cg07994696	-1,36	9,61918E-07	0,000195514	-0,21	16	5'UTR	Island
		pHCC	cg04359558	1,47	0,001119953	0,025569993	0,24	16		
		pHCC	cg08767044	-1,86	0,000525378	0,01721716	-0,29	16	5'UTR	N_Shore
2	NID1	HGDN	cg26837399	1,91	0,000169018	0,005720422	0,28	1	Body	
		eHCC	cg18765906	1,65	0,000435298	0,005152185	0,26	1	Body	
		eHCC	cg26837399	2,51	5,20315E-05	0,001606117	0,34	1	Body	
		pHCC	cg26837399	2,01	0,001536239	0,030121025	0,28	1	Body	
2	PDE4DIP	HGDN	cg19084726	1,56	1,23612E-05	0,001092258	0,23	1	Body	Island
		eHCC	cg15743907	1,28	0,000193912	0,0032557	0,20	1	Body	
		eHCC	cg19084726	2,14	9,39715E-07	0,000193256	0,33	1	Body	Island
		pHCC	cg19084726	1,53	0,000137731	0,008364683	0,22	1	Body	Island
2	PURA	HGDN	cg21778810	-1,39	2,46686E-05	0,001708783	-0,20	5	TSS200	Island
		eHCC	cg21778810	-1,56	1,56117E-05	0,000839329	-0,23	5	TSS200	Island
		pHCC	cg21778810	-1,33	0,000206234	0,0104749	-0,20	5	TSS200	Island
2	SCAMP1	HGDN	cg03056766	-1,40	2,67704E-05	0,001797422	-0,21	5	TSS200	Island
		eHCC	cg03056766	-1,76	4,96589E-06	0,000457026	-0,25	5	TSS200	Island
		pHCC	cg03056766	-1,60	3,66992E-05	0,004025442	-0,23	5	TSS200	Island
2	SMARCC1	HGDN	cg19134770	1,34	1,74423E-06	0,000332913	0,20	3	Body	
		eHCC	cg19134770	1,54	1,20461E-06	0,000220373	0,22	3	Body	
		pHCC	cg19134770	1,49	7,21492E-06	0,001641008	0,21	3	Body	
2	STRN4	HGDN	cg12254611	-1,36	1,84822E-06	0,000345427	-0,23	19	Body	Island

		eHCC	cg12254611	-1,51	1,06453E-06	0,000206487	-0,25	19	Body	Island
		pHCC	cg12254611	-1,45	4,50614E-06	0,001282109	-0,24	19	Body	Island
2	THSD7A	HGDN	cg24676244	1,32	0,000493582	0,011099535	0,21	7	TSS1500	
		eHCC	cg01797590	1,35	0,003258392	0,017732131	0,22	7	TSS200	
		eHCC	cg09557034	1,52	0,000351578	0,004559064	0,24	7	TSS1500	
		eHCC	cg15090509	1,62	0,002053542	0,01318321	0,25	7	TSS1500	
		eHCC	cg24676244	1,88	4,04352E-05	0,001395682	0,30	7	TSS1500	
		pHCC	cg09557034	1,42	0,001686719	0,03163698	0,22	7	TSS1500	
		pHCC	cg24676244	1,61	0,00051808	0,017128325	0,25	7	TSS1500	
		pHCC	cg01797590	1,57	0,002863191	0,04133531	0,25	7	TSS200	
2	UGT2B15	HGDN	cg09189601	1,49	0,003971787	0,03949362	0,22	4	Body	
		eHCC	cg04016992	1,64	2,44741E-07	9,7931E-05	0,25	4	TSS1500	
		eHCC	cg09189601	1,95	0,000765252	0,007201589	0,26	4	Body	
		pHCC	cg04016992	1,48	2,87402E-06	0,001018849	0,22	4	TSS1500	
		pHCC	cg22161115	1,48	0,000906906	0,022917989	0,22	4	1stExon	
		pHCC	cg09189601	1,79	0,002657726	0,039809857	0,26	4	Body	
2	ZNF540	HGDN	cg03975694	1,60	1,19877E-05	0,001072227	0,24	19	5'UTR	S_Shelf
		eHCC	cg03975694	1,95	7,45205E-06	0,00056898	0,30	19	5'UTR	S_Shelf
		pHCC	cg03975694	1,31	0,00146315	0,029389607	0,21	19	5'UTR	S_Shelf
2	ZNF876P	HGDN	cg11836119	1,97	9,34657E-07	0,00022915	0,29	4	Body	Island
		eHCC	cg20296343	1,25	0,002028226	0,01307653	0,21	4	Body	Island
		eHCC	cg12547166	1,40	0,000573854	0,006061744	0,23	4	Body	Island
		eHCC	cg18005867	1,58	0,00023191	0,003604759	0,25	4	TSS200	N_Shore
		eHCC	cg23063647	1,71	0,000115482	0,002463327	0,26	4	TSS200	N_Shore
		eHCC	cg11836119	2,48	3,41294E-07	0,00011561	0,38	4	Body	Island
		pHCC	cg11836119	1,71	0,000117359	0,00764703	0,25	4	Body	Island

2	TMC4	HGDN	cg19488620	1,56	0,000578165	0,012265588	0,25	19	Body	Island
		eHCC	cg19488620	1,85	0,00038676	0,004812381	0,30	19	Body	Island
		pHCC	cg22710065	1,61	0,004008046	0,049097195	0,25	19	Body	Island
2	LTBP2	HGDN	cg17335494	1,33	2,95183E-06	0,00045394	0,20	14	Body	Island
		eHCC	cg17335494	1,71	9,7008E-07	0,000195616	0,24	14	Body	Island
		pHCC	cg17335494	1,63	9,31953E-06	0,001869959	0,24	14	Body	Island
2	HSPE1	HGDN	cg04657470	-1,82	0,00398068	0,039544539	-0,24	2	1stExon	Island
		eHCC	cg04657470	-1,77	0,005671351	0,025560944	-0,22	2	1stExon	Island
		pHCC	cg04657470	-1,95	0,003694816	0,047015393	-0,24	2	1stExon	Island
2	DLG5	HGDN	cg05445097	1,67	6,67974E-09	1,7064E-05	0,21	10	Body	
		eHCC	cg05445097	2,05	2,55651E-09	1,56739E-05	0,24	10	Body	
		eHCC	cg23818888	1,33	0,000224927	0,003545275	0,22	10	Body	S_Shore
		eHCC	cg27586487	1,48	0,000466056	0,005359283	0,24	10	Body	S_Shore
		pHCC	cg05445097	2,12	1,03822E-08	0,000111982	0,24	10	Body	
3	ALDH4A1	eHCC	cg12461099	1,311	0,000199472	0,00330651	0,215	1	TSS1500	
		pHCC	cg22390041	1,252	0,002907524	0,041666014	0,209	1	Body	N_Shelf
		pHCC	cg12461099	1,277	0,00091188	0,022979088	0,214	1	TSS1500	
3	ATG4B	eHCC	cg08969328	1,512	2,43749E-07	9,77949E-05	0,221	2	Body	N_Shelf
		pHCC	cg08969328	1,362	3,86783E-06	0,001194488	0,203	2	Body	N_Shelf
3	ATP6V0A1	eHCC	cg07408552	-1,591	0,001281942	0,02742878	-0,212	17	5'UTR	S_Shore
		pHCC	cg07408552	-1,591	0,001281942	0,02742878	-0,212	17	5'UTR	S_Shore
3	ATP6V1C1	eHCC	cg03506193	-1,801	0,000104466	0,002333175	-0,230	8	5'UTR	S_Shore

		pHCC	cg03506193	- 1,530	0,001025482	0,024401386	-0,203	8	5'UTR	S_Shore
3	C1QTNF4	eHCC	cg05537653	1,685	5,2212E-06	0,000470519	0,232	11	Body	Island
		eHCC	cg17282004	1,676	0,000105627	0,002347565	0,270	11	5'UTR	N_Shore
		pHCC	cg18356785	1,546	0,00039042	0,01477456	0,224	11	Body	Island
		pHCC	cg05537653	2,027	2,75848E-06	0,00099236	0,264	11	Body	Island
3	C9orf3	eHCC	cg13853813	1,452	0,000110657	0,002407528	0,203	9	Body	N_Shelf
		eHCC	cg14375632	1,579	0,00017766	0,003106196	0,221	9	Body	N_Shelf
		pHCC	cg13853813	1,358	0,000772395	0,021106496	0,203	9	Body	N_Shelf
		pHCC	cg14582550	1,313	0,001540008	0,030163251	0,216	9	Body	
		pHCC	cg14375632	1,557	0,000684581	0,019814655	0,218	9	Body	N_Shelf
3	CCDC57	eHCC	cg12879038	1,475	3,08029E-05	0,001212628	0,218	17	Body	S_Shelf
		eHCC	cg22142205	1,561	3,40991E-06	0,000375385	0,239	17	Body	
		pHCC	cg22142205	1,339	4,60311E-05	0,004580184	0,202	17	Body	
3	CCDC84	eHCC	cg27211899	1,460	7,64443E-07	0,000174033	0,239	11	Body	S_Shelf
		pHCC	cg27211899	1,484	2,90065E-06	0,001024245	0,243	11	Body	S_Shelf
3	CHERP	eHCC	cg07688052	1,963	0,000193902	0,00325567	0,235	19	Body	S_Shore
		pHCC	cg07688052	1,977	0,00052654	0,017226223	0,236	19	Body	S_Shore
3	CHST11	eHCC	cg11739675	1,630	0,008780547	0,034372311	0,214	12	Body	Island
		eHCC	cg22260952	1,778	0,002707995	0,015736824	0,244	12	Body	S_Shore
		eHCC	cg17844339	- 2,548	0,000680007	0,006717223	-0,236	12	Body	Island
		eHCC	cg07696842	- 1,292	0,000379752	0,004759914	-0,214	12	Body	
		eHCC	cg12529671	-	0,002815763	0,016142203	-0,209	12	Body	S_Shore

				1,669						
		pHCC	cg01964337	1,281	0,001199658	0,026492301	0,206	12	Body	
		pHCC	cg07696842	- 1,403	0,000652404	0,019299821	-0,227	12	Body	
		pHCC	cg16861964	- 1,356	0,000308331	0,013052779	-0,227	12	Body	
		pHCC	cg22827210	- 1,310	0,000187928	0,009964452	-0,220	12	Body	
3	CLEC14A	eHCC	cg16404157	1,378	0,008322495	0,033120954	0,221	14	1stExon	Island
		eHCC	cg05057720	1,801	0,000327148	0,004377465	0,274	14	1stExon	Island
		pHCC	cg05057720	1,655	0,00163418	0,031111332	0,247	14	1stExon	Island
3	CMYA5	eHCC	cg10257870	1,312	0,000730051	0,00700232	0,212	5	TSS200	
		eHCC	cg09481121	1,384	0,000230309	0,003590465	0,222	5	TSS1500	
		eHCC	cg03546977	1,413	9,60515E-05	0,002228094	0,228	5	TSS200	
		pHCC	cg11438310	1,219	0,000875076	0,022496643	0,202	5	TSS1500	
		pHCC	cg09481121	1,407	0,000593429	0,018400473	0,229	5	TSS1500	
		pHCC	cg00611789	1,395	0,003783493	0,047602404	0,231	5	TSS1500	
		pHCC	cg10257870	1,414	0,000952755	0,023470697	0,235	5	TSS200	
3	CNKSR1	eHCC	cg17330765	1,378	0,001125212	0,025635624	0,214	1	TSS1500	
		pHCC	cg17330765	1,378	0,001125212	0,025635624	0,214	1	TSS1500	
3	CRHBP	eHCC	cg01071966	1,381	0,000729558	0,006998839	0,227	5	1stExon	N_Shore
		pHCC	cg05884167	2,191	0,000227405	0,011034262	0,209	5	Body	S_Shelf
3	CRYBB3	eHCC	cg19288514	1,609	1,67785E-08	3,16982E-05	0,266	22	TSS1500	
		pHCC	cg19288514	1,499	2,37709E-07	0,000328142	0,250	22	TSS1500	
3	CUL3	eHCC	cg01474011	1,560	0,001798314	0,012124535	0,215	2	Body	

		pHCC	cg25502818	1,260	0,00276786	0,040613403	0,212	2	Body	
3	DNASE1L2	eHCC	cg06235653	1,330	0,003322905	0,017966376	0,210	16	Body	Island
		eHCC	cg00249383	1,412	0,000227655	0,003568218	0,228	16	Body	Island
		pHCC	cg00249383	1,286	0,00173383	0,032096978	0,209	16	Body	Island
		pHCC	cg06235653	1,596	0,00210258	0,035423104	0,256	16	Body	Island
3	DNHD1	eHCC	cg10383568	1,500	2,51893E-05	0,001084113	0,249	11	Body	N_Shelf
		pHCC	cg10383568	1,297	0,000442103	0,015777083	0,218	11	Body	N_Shelf
3	EGFLAM	eHCC	cg25344265	-1,796	0,000173692	0,003066918	-0,269	5	Body	
		eHCC	cg18855621	-1,923	7,58721E-06	0,000574267	-0,241	5	Body	
		eHCC	cg08264481	-1,610	6,30507E-06	0,000520237	-0,232	5	Body	
		eHCC	cg11475323	-1,906	7,30788E-05	0,001921857	-0,205	5	Body	S_Shelf
		pHCC	cg25625968	1,250	0,001287529	0,027495665	0,201	5	3'UTR	
3	ELF1	eHCC	cg18456803	-1,514	0,000796076	0,007373515	-0,253	13	TSS200	
		pHCC	cg18456803	-1,559	0,00183015	0,033032031	-0,242	13	TSS200	
3	EXOC2	eHCC	cg04789318	1,427	5,31979E-08	4,95599E-05	0,216	6	5'UTR	N_Shelf
		pHCC	cg04789318	1,402	4,21574E-07	0,000397163	0,211	6	5'UTR	N_Shelf
3	FAM20B	eHCC	cg24997888	1,509	0,000260012	0,003847161	0,227	1	3'UTR	
		pHCC	cg24997888	1,610	0,000529955	0,017284303	0,243	1	3'UTR	

3	FGFR4	eHCC	cg12982374	1,408	0,00032384	0,004351191	0,211	5	Body	S_Shelf
		eHCC	cg17386911	1,617	0,000907565	0,007963218	0,223	5	Body	S_Shelf
		pHCC	cg12982374	1,289	0,002217601	0,036337706	0,201	5	Body	S_Shelf
3	FNDC1	eHCC	cg09107912	1,687	0,000231206	0,003597966	0,243	6	TSS1500	Island
		eHCC	cg00157796	1,567	0,000243301	0,003702274	0,257	6	TSS200	Island
		pHCC	cg07739841	-1,542	0,001018914	0,02432998	-0,243	6	Body	S_Shelf
3	GAS7	eHCC	cg26999423	-1,929	0,000418194	0,005037802	-0,298	17	Body	
		eHCC	cg12091339	-1,922	0,000632805	0,006428996	-0,230	17	Body	
		eHCC	cg07049421	-1,479	0,001028094	0,008569715	-0,226	17	Body	
		eHCC	cg02605292	-1,670	0,000670632	0,006664488	-0,201	17	Body	
		pHCC	cg26999423	-1,844	0,002011096	0,03465132	-0,288	17	Body	
		pHCC	cg06130714	-1,369	0,000397627	0,01491624	-0,223	17	Body	
3	GLTSCR1	eHCC	cg22461472	1,621	8,72305E-05	0,002116745	0,215	19	Body	S_Shelf
		pHCC	cg22461472	1,529	0,000577861	0,018119573	0,211	19	Body	S_Shelf
3	HELZ	eHCC	cg18432877	1,498	0,000393989	0,004866668	0,215	17	Body	
		pHCC	cg18432877	1,791	0,000286473	0,012544729	0,239	17	Body	
3	HIPK1	eHCC	cg17588904	1,330	4,52477E-07	0,000133055	0,218	1	Body	
		pHCC	cg17588904	1,402	1,11097E-06	0,00064397	0,223	1	Body	
3	HIST1H3H	eHCC	cg01330280	-	0,001917359	0,012611879	-0,259	6	TSS1500	N_Shore

				1,640						
		pHCC	cg01330280	-1,959	0,001474903	0,029509664	-0,321	6	TSS1500	N_Shore
3	HK3	eHCC	cg19960778	-1,970	4,63067E-05	0,001504119	-0,253	5	Body	S_Shore
		eHCC	cg11093640	-1,740	0,00019669	0,003278867	-0,235	5	3'UTR	S_Shelf
		eHCC	cg06485139	-1,614	0,000826268	0,007540737	-0,232	5	Body	N_Shore
		eHCC	cg04875020	-1,426	0,000132017	0,002646224	-0,215	5	Body	N_Shore
		pHCC	cg04875020	-1,528	0,000241237	0,011384661	-0,231	5	Body	N_Shore
3	ITGA11	eHCC	cg25699851	-1,984	8,93371E-05	0,00214507	-0,272	15	Body	
		eHCC	cg26217827	2,600	0,014278532	0,048152421	0,308	15	3'UTR	
		pHCC	cg08872353	-1,552	0,000233928	0,011203012	-0,244	15	Body	N_Shelf
		pHCC	cg24213777	-1,231	0,000419666	0,015334923	-0,203	15	Body	
3	ITGAM	eHCC	cg02256631	1,316	0,006135867	0,026958651	0,205	16	Body	Island
		pHCC	cg22490695	-1,251	0,001683579	0,031611329	-0,207	16	TSS200	
3	ITGB3	eHCC	cg03460756	1,879	9,40275E-05	0,002201982	0,294	17	Body	
		pHCC	cg03460756	1,540	0,001842825	0,033141581	0,250	17	Body	
3	KAT2A	eHCC	cg16550651	1,846	5,26457E-05	0,001615827	0,249	17	Body	N_Shore
		pHCC	cg16550651	1,690	0,000494374	0,016744925	0,250	17	Body	N_Shore

3	KCNS2	eHCC	cg08706670	1,440	0,006555951	0,028188336	0,234	8	TSS200	Island
		eHCC	cg11964564	1,668	0,002889448	0,016412185	0,250	8	5'UTR	Island
		eHCC	cg14486338	2,317	1,65125E-06	0,000257477	0,363	8	Body	Island
		eHCC	cg14688104	3,040	0,001682393	0,011622988	0,371	8	1stExon	Island
		pHCC	cg14688104	3,469	0,001847787	0,033182257	0,415	8	1stExon	Island
3	KIAA1875	eHCC	cg01423393	1,582	1,71307E-05	0,000880983	0,214	8	Body	Island
		pHCC	cg01423393	1,452	7,2645E-05	0,005914085	0,201	8	Body	Island
3	KLHL5	eHCC	cg08217447	1,690	0,0006228	0,006366254	0,247	4	TSS1500	
		pHCC	cg08217447	1,699	0,001756363	0,032326742	0,254	4	TSS1500	
3	LPP	eHCC	cg04423294	1,339	3,16552E-05	0,001230227	0,209	3	Body	
		pHCC	cg24454374	1,213	0,003067089	0,042809967	0,200	3	5'UTR	
3	LZTS1	eHCC	cg05796178	-2,089	2,50859E-05	0,001081459	-0,268	8	Body	S_Shelf
		pHCC	cg13583414	-1,575	0,000643847	0,019167934	-0,228	8	Body	S_Shelf
			cg05796178	-1,815	0,000349394	0,013922142	-0,223	8	Body	S_Shelf
3	MAST2	eHCC	cg02835462	1,233	0,000386466	0,004810093	0,207	1	Body	
		pHCC	cg02835462	1,361	0,000575537	0,018077614	0,227	1	Body	
3	MATN2	eHCC	cg19987349	1,429	3,71774E-07	0,000120037	0,218	8	Body	
		pHCC	cg19987349	1,433	2,04715E-06	0,000862524	0,214	8	Body	
3	MCM6	eHCC	cg11446240	-1,595	3,51221E-05	0,001299532	-0,226	2	Body	N_Shore

		pHCC	cg11446240	-1,579	0,000157345	0,00900492	-0,225	2	Body	N_Shore
3	MED14	eHCC	cg05812657	1,608	2,15541E-08	3,44223E-05	0,234	X	Body	N_Shelf
		pHCC	cg05812657	1,687	6,30141E-08	0,000183304	0,240	X	Body	N_Shelf
3	MEF2C	eHCC	cg24124703	-1,438	0,005506455	0,02505221	-0,217	5	5'UTR	
		pHCC	cg24124703	-1,861	0,002258426	0,036679761	-0,262	5	5'UTR	
3	MICA	eHCC	cg23826579	1,546	1,30982E-07	7,31567E-05	0,251	6	Body	S_Shelf
		pHCC	cg23826579	1,286	4,33075E-06	0,001248971	0,210	6	Body	S_Shelf
3	MYH14	eHCC	cg11992783	1,724	0,000115076	0,002459796	0,206	19	Body	Island
		pHCC	cg11992783	1,821	0,000215926	0,010733716	0,216	19	Body	Island
3	NAA30	eHCC	cg03318573	1,291	0,000488664	0,005512554	0,209	14	TSS1500	N_Shore
		pHCC	cg03318573	1,320	0,001343153	0,028088478	0,212	14	TSS1500	N_Shore
3	NRP2	eHCC	cg17455088	1,335	0,001262114	0,009722315	0,211	2	Body	S_Shore
		eHCC	cg05348875	1,283	3,85549E-05	0,001361272	0,214	2	Body	
		eHCC	cg10648139	1,809	0,000334581	0,004435961	0,271	2	Body	S_Shore
		eHCC	cg22367989	2,896	2,9864E-05	0,001191996	0,399	2	TSS1500	Island
		pHCC	cg17455088	1,325	0,003927781	0,048570256	0,213	2	Body	S_Shore
3	PPAP2B	eHCC	cg10500503	1,244	0,002311455	0,014220871	0,207	1	Body	
		pHCC	cg22396959	1,366	6,53391E-05	0,005585362	0,214	1	Body	
3	PALM3	eHCC	cg11437328	1,636	0,000103987	0,002327386	0,220	19	TSS1500	S_Shelf
		pHCC	cg11437328	1,471	0,001021355	0,024356157	0,215	19	TSS1500	S_Shelf

3	PAQR6	eHCC	cg24152297	1,526	4,7237E-08	4,66868E-05	0,231	1	TSS1500	S_Shelf	
		pHCC	cg24152297	1,614	1,27344E-07	0,000245382	0,240	1	TSS1500	S_Shelf	
3	PCNX	eHCC	cg14009504	1,541	9,29995E-07	0,000192231	0,213	14	Body		
		pHCC	cg14009504	1,906	3,95852E-07	0,000388937	0,249	14	Body		
3	PIK3CG	eHCC	cg13214190	-1,668	0,007053349	0,029621361	-0,271	7	TSS200	N_Shelf	
		eHCC	cg00604356	-1,636	0,000843102	0,007627393	-0,263	7	5'UTR	N_Shore	
		pHCC	cg00510718	-3,046	0,00093089	0,023196776	-0,444	7	5'UTR	N_Shelf	
		pHCC	cg13214190	-2,422	0,001360526	0,028282152	-0,365	7	TSS200	N_Shelf	
		pHCC	cg15881332	-2,204	0,000849698	0,022174918	-0,331	7	TSS200	N_Shelf	
		pHCC	cg08779777	-2,115	0,000618061	0,018803362	-0,314	7	TSS200	N_Shelf	
		pHCC	cg00661777	-2,053	0,000411995	0,01518033	-0,307	7	Body	S_Shelf	
		pHCC	cg11982525	-1,933	0,000945677	0,023385181	-0,303	7	TSS1500	N_Shelf	
		pHCC	cg00604356	-1,588	0,003205808	0,04376952	-0,261	7	5'UTR	N_Shore	
3	PLEKHF2	eHCC	cg12930753	1,667	0,000191699	0,003233648	0,263	8	3'UTR		
		pHCC	cg12930753	1,394	0,00291337	0,041710621	0,233	8	3'UTR		
3	PPM1D	eHCC	cg04180177	1,252	2,06493E-06	0,000288918	0,205	17	Body		
		pHCC	cg04180177	1,270	7,28475E-06	0,001650356	0,209	17	Body		

3	PRMT7	eHCC	cg10061770	1,574	0,000132487	0,002651869	0,230	16	Body	
		pHCC	cg10061770	1,419	0,00119487	0,026433786	0,204	16	Body	
3	PRR5	eHCC	cg04607412	1,445	0,000633153	0,006430784	0,210	22	5'UTR	
		pHCC	cg04607412	1,764	0,000376057	0,014484217	0,262	22	5'UTR	
3	PTBP1	eHCC	cg19373090	-1,427	2,20224E-07	9,30733E-05	-0,206	19	TSS200	Island
		pHCC	cg19373090	-1,409	7,89021E-07	0,00054168	-0,204	19	TSS200	Island
3	PTPRJ	eHCC	cg04462547	1,365	4,60958E-07	0,000134134	0,208	11	Body	S_Shelf
		eHCC	cg06298729	1,712	0,000239524	0,003671033	0,211	11	Body	
		pHCC	cg04462547	1,358	1,99131E-06	0,000854347	0,206	11	Body	S_Shelf
3	REEP3	eHCC	cg10300729	1,531	1,98415E-07	8,90064E-05	0,237	10	Body	S_Shelf
		pHCC	cg10300729	1,475	1,27E-06	0,000690733	0,228	10	Body	S_Shelf
3	RGS10	eHCC	cg19653161	1,549	0,002574817	0,01522054	0,226	10	TSS1500	Island
		eHCC	cg17527393	2,662	1,27339E-05	0,000756324	0,260	10	TSS200	Island
		pHCC	cg10200202	-1,539	0,000977178	0,02378008	-0,255	10	Body	
3	RNF220	eHCC	cg24603481	-1,697	0,000641549	0,0064862	-0,214	1	Body	S_Shore
		eHCC	cg01422881	1,406	0,000312242	0,004260469	0,237	1	5'UTR	Island
		pHCC	cg09860921	1,317	0,001947444	0,034084364	0,215	1	Body	N_Shelf
3	SCAND3	eHCC	cg19747271	-1,425	3,52492E-05	0,001301472	-0,225	6	Body	N_Shore
		eHCC	cg22302929	1,695	1,91757E-05	0,000934563	0,258	6	1stExon	N_Shore

		pHCC	cg19747271	- 1,494	8,53122E-05	0,006437328	-0,237	6	Body	N_Shore
		pHCC	cg22302929	1,305	0,00097212	0,023718003	0,208	6	1stExon	N_Shore
3	SKI	eHCC	cg12483545	1,672	0,002420813	0,014641106	0,230	1	Body	
		pHCC	cg12580943	1,254	0,002315354	0,037158143	0,210	1	Body	
3	SLC25A27	eHCC	cg19101566	1,936	0,00027258	0,003947906	0,288	6	Body	S_Shelf
		pHCC	cg19101566	2,029	0,000566117	0,017929972	0,291	6	Body	S_Shelf
3	SORBS1	eHCC	cg27111150	1,375	6,52052E-05	0,001804757	0,217	10	Body	
		pHCC	cg27111150	1,255	0,000608058	0,018646515	0,202	10	Body	
3	SPN	eHCC	cg02030929	- 1,627	0,000442249	0,00519771	-0,228	16	Body	Island
		pHCC	cg09946623	- 1,307	0,000805557	0,02156554	-0,205	16	TSS1500	N_Shore
3	SWAP70	eHCC	cg08213398	1,431	8,23954E-07	0,000181688	0,212	11	Body	
		pHCC	cg08213398	1,389	6,41799E-06	0,001555998	0,206	11	Body	
3	TAF7L	eHCC	cg19386336	1,513	0,000545741	0,005886281	0,210	X	Body	Island
		eHCC	cg01538344	1,525	0,000402553	0,004930357	0,244	X	1stExon	Island
		pHCC	cg01538344	1,332	0,003016047	0,042460241	0,213	X	1stExon	Island
3	TIGD1	eHCC	cg18346402	1,518	3,05397E-07	0,000108993	0,249	2	1stExon	N_Shelf
		pHCC	cg18346402	1,534	1,59034E-06	0,000776591	0,252	2	1stExon	N_Shelf
3	TNS1	eHCC	cg06320380	1,744	4,86194E-05	0,001545514	0,273	2	5'UTR	
		eHCC	cg09548780	2,360	1,21401E-05	0,000736362	0,334	2	5'UTR	
		pHCC	cg12681370	1,368	0,000356473	0,014046256	0,218	2	5'UTR	

3	TSC2	eHCC	cg07730183	1,854	0,0001864	0,003185782	0,212	16	Body	S_Shore
		eHCC	cg06094085	1,503	0,001664462	0,011549222	0,215	16	Body	S_Shore
		eHCC	cg02504384	1,452	0,000417413	0,005032416	0,220	16	Body	N_Shelf
		eHCC	cg02364279	1,929	0,000903404	0,007942003	0,232	16	Body	N_Shore
		eHCC	cg06330323	1,594	0,000949283	0,008178712	0,246	16	Body	N_Shore
		pHCC	cg07730183	1,714	0,001321892	0,027828249	0,208	16	Body	S_Shore
3	TXNDC5	eHCC	cg11401394	1,712	0,000266358	0,003897322	0,255	6	Body	
		pHCC	cg11401394	1,730	0,0008659	0,022381669	0,266	6	Body	
3	UCN	eHCC	cg05113927	1,744	9,60018E-07	0,000195291	0,223	2	TSS200	Island
		eHCC	cg01454215	1,562	0,002163461	0,013625739	0,229	2	3'UTR	Island
		pHCC	cg05113927	1,519	2,51897E-05	0,00325082	0,202	2	TSS200	Island
3	ZDHHC7	eHCC	cg03655147	1,327	6,80724E-06	0,000542317	0,220	16	5'UTR	N_Shelf
		eHCC	cg09735674	-1,240	1,76519E-05	0,00089602	-0,208	16	5'UTR	Island
		pHCC	cg03655147	1,331	1,914E-05	0,002779045	0,215	16	5'UTR	N_Shelf
3	ZMIZ2	eHCC	cg11425656	1,311	2,80346E-05	0,001149676	0,204	7	5'UTR	S_Shelf
		pHCC	cg11425656	1,361	8,29045E-05	0,006348372	0,215	7	5'UTR	S_Shelf
3	ZNF366	eHCC	cg11905892	-2,567	0,002712455	0,015752691	-0,291	5	Body	Island
		eHCC	cg22558265	-2,606	0,000212609	0,003430324	-0,246	5	Body	Island
		eHCC	cg00657287	-1,836	0,000982352	0,008344657	-0,218	5	Body	N_Shore
		pHCC	cg04454664	1,253	0,000262241	0,011943707	0,207	5	Body	

3	EBF3	eHCC	cg22952849	-2,310	3,09135E-05	0,001214837	-0,301	10	Body	N_Shore	
		eHCC	cg07506153	-2,606	0,005569163	0,025243439	-0,288	10	Body	N_Shore	
		eHCC	cg20800606	-1,834	0,00067282	0,006677521	-0,256	10	Body	Island	
		eHCC	cg27454298	-2,091	0,000271399	0,003938483	-0,241	10	Body	Island	
		eHCC	cg10598433	-2,212	0,000352589	0,004565217	-0,224	10	Body	S_Shore	
		eHCC	cg02337436	-2,071	0,000458332	0,005307685	-0,213	10	Body		
		eHCC	cg03128163	-1,594	0,000112163	0,00242401	-0,209	10	Body	N_Shore	
		eHCC	cg15991708	-1,586	6,8076E-05	0,0018482	-0,206	10	Body		
		eHCC	cg19582265	-1,455	6,81884E-05	0,001850109	-0,218	10	TSS1500		
		pHCC	cg04043455								
3	FAM189A1	eHCC	cg19263124	-2,078	5,56733E-05	0,00166393	-0,239	15	Body		
		eHCC	cg18512553	-2,042	0,000129207	0,002616178	-0,237	15	Body		
		eHCC	cg12711059	-1,486	9,64515E-05	0,002233551	-0,226	15	3'UTR		
		eHCC	cg08904058	-1,260	0,00010854	0,002382418	-0,212	15	Body		
		eHCC	cg21207450	-1,460	0,000366217	0,004665259	-0,200	15	Body		
		pHCC	cg12711059	-1,510	0,000319038	0,013289653	-0,227	15	3'UTR		

		pHCC	cg25941083	- 1,342	0,000934362	0,023252263	-0,227	15	Body	N_Shore
3	GPR158	eHCC	cg14506260	1,307	0,007127305	0,029833619	0,207	10	Body	
		eHCC	cg24361761	2,232	0,00041694	0,005029219	0,345	10	Body	
		pHCC	cg24361761					10	Body	
3	IRX3	eHCC	cg08279075	1,763	0,001841239	0,012304077	0,271	16	Body	Island
		pHCC	cg05001964	1,328	0,001413256	0,028877383	0,203	16	Body	Island
3	KRTCAP3	eHCC	cg17158414	1,739	0,001462983	0,010658876	0,216	2	1stExon	Island
		eHCC	cg11618577	1,529	0,004597055	0,022223653	0,220	2	Body	Island
		eHCC	cg24768116	1,661	0,001495017	0,010809349	0,236	2	TSS200	Island
		eHCC	cg04845466	1,869	0,000128646	0,002610293	0,246	2	TSS200	Island
		eHCC	cg02592271	1,881	0,004601606	0,022239456	0,247	2	Body	Island
		pHCC	cg17158414	1,773	0,003062764	0,042779192	0,219	2	1stExon	Island
		pHCC	cg04845466	1,893	0,000360845	0,014146618	0,234	2	TSS200	Island
3	TMEM18	eHCC	cg27237671	1,418	0,000653957	0,006562296	0,227	2	Body	N_Shore
		pHCC	cg27237671	1,327	0,003406437	0,045094384	0,213	2	Body	N_Shore
3	WNT7A	eHCC	cg23655615	- 1,817	0,00125122	0,009675403	-0,243	3	Body	Island
		eHCC	cg10460033	- 1,524	0,000259	0,003837685	-0,239	3	Body	N_Shore
		eHCC	cg21224025	- 1,467	0,001549776	0,011053473	-0,214	3	Body	S_Shelf
		pHCC	cg10460033	- 1,350	0,002408034	0,03792216	-0,214	3	Body	N_Shore
3	ZYG11A	eHCC	cg20287790	2,232	1,67669E-06	0,000259292	0,242	1	3'UTR	

		pHCC	cg20287790	1,688	0,000170838	0,009443107	0,217	1	3'UTR	
3	APC2	eHCC	cg19333963	1,974	0,003810961	0,01964788	0,314	19	Body	Island
		eHCC	cg03306486	2,479	0,002036304	0,013109485	0,378	19	Body	Island
		eHCC	cg10808783	2,602	0,000248662	0,003747633	0,385	19	Body	Island
		pHCC	cg10808783							
3	CBFA2T3	eHCC	cg27434245	1,444	0,000527098	0,005763933	0,203	16	1stExon	Island
		eHCC	cg04072301	-2,046	8,47368E-06	0,000612218	-0,306	16	5'UTR	Island
		eHCC	cg02633398	-1,408	0,000986554	0,008367024	-0,223	16	5'UTR	N_Shore
		eHCC	cg03021297	-1,821	0,001129361	0,009079844	-0,215	16	5'UTR	
		pHCC	cg00762678	-1,265	0,000881122	0,022581799	-0,208		5'UTR	N_Shelf
		pHCC	cg04699663	-1,242	0,000962478	0,023596265	-0,203		1stExon	
3	CNOT4	eHCC	cg25871890	1,457	0,000747295	0,007102994	0,234	7	5'UTR	
		pHCC	cg25871890	1,532	0,001348339	0,02815058	0,239	7	5'UTR	
3	COPA	eHCC	cg09866659	1,500	5,83221E-08	5,15072E-05	0,252	1	Body	
		pHCC	cg09866659	1,327	1,35954E-06	0,000711844	0,222	1	Body	
3	CR1L	eHCC	cg06917617	1,306	0,000190517	0,003222883	0,216	1	Body	Island
		pHCC	cg06917617	1,584	0,000117677	0,007654385	0,257	1	Body	Island
3	CREBBP	eHCC	cg03368634	2,023	3,19922E-05	0,001236525	0,296	16	Body	
		pHCC	cg03368634	1,434	0,00266988	0,039905231	0,233	16	Body	

3	CYP1A2	eHCC	cg04968473	1,677	0,0002478	0,00374116	0,250	15	TSS1500	
		pHCC	cg04968473	1,409	0,003353047	0,044745176	0,208	15	TSS1500	
3	HDAC9	eHCC	cg04892643	-1,653	3,80242E-06	0,000396133	-0,271	7	Body	
		eHCC	cg16925459	-1,402	9,45234E-05	0,002208479	-0,227	7	Body	
		eHCC	cg08285151	-1,358	0,001998609	0,012949354	-0,221	7	TSS1500	
		pHCC	cg08285151	-1,717	0,000921164	0,023074082	-0,285	7	TSS1500	
		pHCC	cg04892643	-1,379	0,000119477	0,007713594	-0,225	7	Body	
3	HSF5	eHCC	cg25793387	1,796	0,000116076	0,002470337	0,234	17	Body	
		pHCC	cg25793387	1,888	0,000267711	0,012083983	0,246	17	Body	
3	KREMEN2	eHCC	cg26494929	1,560	0,002733419	0,015836891	0,235	16	Body	Island
			cg05169951	2,182	1,22767E-05	0,000741323	0,279	16	Body	Island
		pHCC	cg05169951	1,621	0,00091564	0,023018862	0,219	16	Body	Island
3	S100A6	eHCC	cg01910639	-1,530	2,45761E-06	0,000316155	-0,255	1	Body	N_Shore
		pHCC	cg01910639	-1,265	9,15466E-05	0,006695385	-0,211	1	Body	N_Shore
3	SIPA1L1	eHCC	cg15378445	1,495	3,64569E-08	4,09609E-05	0,228	14	Body	
		eHCC	cg02058870	1,777	0,000376026	0,004733271	0,280	14	5'UTR	
		pHCC	cg15378445	1,570	1,13855E-07	0,000233189	0,234	14	Body	
3	SNORD6	eHCC	cg03531787	1,449	0,000368829	0,00468219	0,206	11	TSS200	
		eHCC	cg06955958	1,558	0,000306768	0,004216591	0,251	11	TSS200	

		pHCC	cg06955958	1,617	0,000778465	0,021187295	0,264	11	TSS200	
3	STX3	eHCC	cg14333691	1,463	1,93006E-08	3,35383E-05	0,224	11	3'UTR	
		pHCC	cg14333691	1,431	1,58326E-07	0,000267759	0,217	11	3'UTR	
3	THRSP	eHCC	cg03493668	1,398	0,000520835	0,005720482	0,229	11	3'UTR	
		pHCC	cg03493668	1,316	0,002383629	0,03772453	0,215	11	3'UTR	
3	TSPAN10	eHCC	cg18268547	1,471	0,000633127	0,006430646	0,236	17	3'UTR	Island
		pHCC	cg18268547	1,340	0,004026282	0,049224879	0,214	17	3'UTR	Island
3	USP42	eHCC	cg13977660	1,531	9,01962E-07	0,000189354	0,252	7	5'UTR	S_Shelf
		pHCC	cg13977660	1,340	0,004026282	0,049224879	0,214	7	5'UTR	S_Shelf
3	ACTA2	eHCC	cg03755566	1,682	8,13988E-06	0,000598617	0,271	10	Body	
		pHCC	cg03755566	1,474	0,000151188	0,008793536	0,235	10	Body	
3	ARHGAP10	eHCC	cg13248315	1,641	1,00649E-07	6,4364E-05	0,244	10	Body	N_Shelf
		pHCC	cg13248315					10	Body	N_Shelf
3	COL14A1	eHCC	cg26179069	-1,453	1,68032E-05	0,000873201	-0,233	8	Body	
		eHCC	cg05830842	-1,454	7,51908E-05	0,001952561	-0,228	8	Body	
		eHCC	cg10133738	-1,759	0,000216387	0,003464022	-0,207	8	3'UTR	
		pHCC	cg05830842	1,442	2,31963E-06	0,00091091	0,215	8	Body	
3	E4F1	eHCC	cg27038935	-1,773	1,68939E-09	1,32594E-05	-0,224	16	TSS200	Island
		pHCC	cg27038935	-	2,30428E-08	0,000133146	-0,215	16	TSS200	Island

				1,689						
3	FAM129A	eHCC	cg05655671	2,061	0,003705965	0,019287685	0,316	1	1stExon	Island
		pHCC	cg17464436	-1,786	4,424E-05	0,004475372	-0,242	1	Body	
3	HS3ST2	eHCC	cg04718102	-2,204	0,000771279	0,007233639	-0,312	16	Body	Island
		eHCC	cg01555981	-2,320	0,001228153	0,00956831	-0,269	16	Body	Island
		eHCC	cg05970721	-1,845	4,04465E-05	0,001395974	-0,236	16	Body	Island
		eHCC	cg10180297	-1,316	3,64699E-05	0,001322484	-0,220	16	Body	S_Shore
		pHCC	cg10180297	-1,351	0,00012067	0,007763727	-0,228	16	Body	S_Shore
3	KIF26A	eHCC	cg11792470	-2,565	0,000299114	0,004154802	-0,291	14	Body	Island
		eHCC	cg01549570	-1,946	5,88539E-05	0,001713924	-0,286	14	Body	Island
		eHCC	cg00742738	-2,041	0,001506599	0,010860826	-0,264	14	Body	N_Shore
		eHCC	cg19800856	-1,845	4,85261E-05	0,001543559	-0,249	14	Body	Island
		eHCC	cg13250679	-1,648	0,001865206	0,012402283	-0,248	14	3'UTR	S_Shore
		eHCC	cg01933329	-2,655	0,001158278	0,009218869	-0,246	14	Body	Island
		eHCC	cg12468119	-1,642	0,004577351	0,022163995	-0,235	14	Body	Island
		eHCC	cg24706981	-	0,001023731	0,008547955	-0,226	14	Body	N_Shore

				1,889						
		eHCC	cg06760280	-1,568	0,000215225	0,003452445	-0,225	14	Body	N_Shore
		eHCC	cg16520046	-1,715	0,000943398	0,008147366	-0,223	14	Body	S_Shore
		eHCC	cg19003626	-1,949	0,000453261	0,00527236	-0,221	14	Body	N_Shore
		eHCC	cg23892535	-1,565	0,001489474	0,010784306	-0,215	14	Body	Island
		eHCC	cg02922817	-1,643	0,000423466	0,005072836	-0,215	14	Body	N_Shore
		eHCC	cg25131771	-1,267	0,000332767	0,004422609	-0,207	14	Body	Island
		pHCC	cg01549570	-1,416	0,002910726	0,041688509	-0,201	14	Body	Island
3	MRVI1	eHCC	cg17299456	-1,554	0,000557138	0,005955705	-0,213	11	5'UTR	
		pHCC	cg17471425							
3	PLVAP	eHCC	cg15219347	-1,836	0,000311766	0,004256726	-0,287	19	TSS1500	S_Shore
		eHCC	cg01662455	-1,315	0,000413255	0,005005784	-0,221	19	1stExon	Island
		eHCC	cg18302786	-1,517	0,000963315	0,008251243	-0,208	19	TSS200	S_Shore
		eHCC	cg10709246	-1,275	0,001799566	0,012130169	-0,205	19	5'UTR	Island
		eHCC	cg23881613	-2,091	8,5276E-06	0,000614467	-0,200	19	3'UTR	Island
		pHCC	cg10178628	-1,401	0,001873357	0,033413199	-0,218	19	Body	

3	TFIP11	eHCC	cg03019505	1,408	2,63095E-07	0,000101671	0,236	22	Body	S_Shelf	
		pHCC	1,303376649	1,303	3,93172E-06	0,001199461	0,218	22	Body	S_Shelf	
3	THBS2	eHCC	cg02997295	-1,743	0,000910415	0,007979071	-0,275	6	Body	S_Shore	
		eHCC	cg04476508	-1,585	0,000897118	0,007909381	-0,251	6	Body	Island	
		eHCC	cg06119452	-1,560	0,000428559	0,005106589	-0,247	6	Body	N_Shore	
		eHCC	cg01320433	-1,502	0,001466524	0,010673409	-0,243	6	Body	N_Shore	
		eHCC	cg02785555	-1,718	0,000533908	0,005807525	-0,239	6	Body	Island	
		eHCC	cg15216858	-2,318	0,000100322	0,002282748	-0,229	6	Body	Island	
		eHCC	cg25631414	-2,019	0,000508566	0,005642518	-0,225	6	Body	Island	
		eHCC	cg00438284	-1,413	8,99616E-05	0,002153094	-0,207	6	Body	S_Shelf	
		eHCC	cg03091070	-1,388	0,003222734	0,017612259	-0,206	6	Body	N_Shore	
		pHCC	cg01320433	-1,695	0,001646196	0,031252485	-0,271	6	Body	N_Shore	
3	ZFP91	eHCC	cg12027899	1,543	1,19831E-05	0,000731878	0,237	11	3'UTR		
		pHCC	cg12027899	1,287	0,000333865	0,013610629	0,209	11	3'UTR		

Supplemental Table 8

Top Networks		
ID	Associated Network Functions	Score
1	Connective Tissue Development and Function, Connective Tissue Disorders, Organ Morphology	43
2	Cellular Development, Cellular Growth and Proliferaton, Embryonic Development	35
3	Cell-To-Cell Signaling and Interaction, Tissue Development, Connective Tissue Development and Function	30
4	Cancer, Organismal Injury and Abnormalities, Cellular Assembly and Organization	30
5	Behavior, Inflammatory Disease	30

Molecular and Cellular Functions		
Name	p-value range	# molecules
Cellular Movement	6.97E-03 – 3.29E-06	46
Cell Morphology	6.97E-03 – 1.52E-05	37
Cell Signaling	5.59E-03 – 2.66E-05	23
Cell-to-Cell Signaling and Interaction	6.97E-03 – 5.70E-05	24
Cellular Development	6.97E-03 – 1.04E-04	55

Supplemental Table 9

Gene_ Symbol	Panel	Probe_ID	HCC_Mean_ Beta	NL_Mean_B eta	Mean_ Beta_ Diff.	Mean_Log2 _Quotient	P-value	FDR P-value	min. 0.2 delta beta	FDR P<0.05	Status in HCC
EGFLAM	List_3	cg25344265	0,409967781	0,892152377	-0,48	-1,103092478	1,28E-62	6,46E-60	Yes	Yes	Hypomethylated in HCC
LZTS1	List_3	cg13583414	0,423408301	0,846738917	-0,42	-0,983128839	7,40E-35	2,54E-33	Yes	Yes	Hypomethylated in HCC
ATP6V0A1	List_3	cg07408552	0,117444216	0,323434138	-0,21	-1,38753592	3,67E-34	1,18E-32	Yes	Yes	Hypomethylated in HCC
LZTS1	List_3	cg05796178	0,47957211	0,887568016	-0,41	-0,874499893	2,52E-33	7,50E-32	Yes	Yes	Hypomethylated in HCC
DNAH17	List_2	cg12071008	0,324331228	0,616963242	-0,29	-0,907102747	3,76E-31	9,04E-30	Yes	Yes	Hypomethylated in HCC
FAM189A1	List_3	cg12711059	0,420513191	0,827788456	-0,41	-0,960528563	1,20E-30	2,74E-29	Yes	Yes	Hypomethylated in HCC
HK3	List_3	cg19960778	0,585752362	0,909082329	-0,32	-0,625481333	5,32E-29	1,02E-27	Yes	Yes	Hypomethylated in HCC
DNAH17	List_2	cg25691430	0,568599408	0,914092755	-0,35	-0,67547282	2,41E-28	4,33E-27	Yes	Yes	Hypomethylated in HCC
HK3	List_3	cg06485139	0,557341962	0,889660212	-0,33	-0,665161647	3,60E-28	6,33E-27	Yes	Yes	Hypomethylated in HCC
GAS7	List_3	cg12091339	0,578969104	0,927306719	-0,35	-0,670329268	4,43E-28	7,73E-27	Yes	Yes	Hypomethylated in HCC
GAS7	List_3	cg07049421	0,472863733	0,849913159	-0,38	-0,832574861	3,16E-27	5,04E-26	Yes	Yes	Hypomethylated in HCC
DNAH17	List_2	cg09705784	0,422492216	0,864495172	-0,44	-1,015776253	1,49E-26	2,22E-25	Yes	Yes	Hypomethylated in HCC
DNAH17	List_	cg11803990	0,539566666	0,884823881	-0,35	-	2,30E-26	3,36E-25	Yes	Yes	Hypomethylated

	2					0,703309258					in HCC
DNAH17	List_2	cg09687005	0,43302694	0,795687382	-0,36	-0,862825729	3,70E-26	5,31E-25	Yes	Yes	Hypomethylated in HCC
ITGA11	List_3	cg25699851	0,531798061	0,913411396	-0,38	-0,769218307	4,65E-26	6,60E-25	Yes	Yes	Hypomethylated in HCC
DNAH17	List_2	cg10375710	0,405588052	0,789004985	-0,38	-0,943050326	8,49E-26	1,17E-24	Yes	Yes	Hypomethylated in HCC
CAMTA1	List_1	cg17143900	0,527163973	0,869862178	-0,34	-0,71191501	1,78E-25	2,37E-24	Yes	Yes	Hypomethylated in HCC
CAMTA1	List_1	cg23021268	0,546600152	0,855672153	-0,31	-0,637179449	2,57E-25	3,37E-24	Yes	Yes	Hypomethylated in HCC
HK3	List_3	cg11093640	0,555667054	0,872346157	-0,32	-0,641391609	3,34E-25	4,33E-24	Yes	Yes	Hypomethylated in HCC
DNAH17	List_2	cg15618347	0,528183589	0,921089717	-0,39	-0,79082179	6,27E-25	7,89E-24	Yes	Yes	Hypomethylated in HCC
CAMTA1	List_1	cg24579970	0,399969814	0,761984604	-0,36	-0,913054388	1,02E-24	1,26E-23	Yes	Yes	Hypomethylated in HCC
CAMTA1	List_1	cg26161885	0,63110525	0,932053367	-0,30	-0,555247566	1,11E-24	1,36E-23	Yes	Yes	Hypomethylated in HCC
DNAH17	List_2	cg25399573	0,588370774	0,949534441	-0,36	-0,681294879	1,21E-24	1,48E-23	Yes	Yes	Hypomethylated in HCC
DNAH17	List_2	cg01341643	0,627660003	0,934551055	-0,31	-0,566841388	1,72E-24	2,07E-23	Yes	Yes	Hypomethylated in HCC
EGFLAM	List_3	cg11475323	0,615334267	0,945301741	-0,33	-0,611328918	2,06E-24	2,46E-23	Yes	Yes	Hypomethylated in HCC
DNAH17	List_2	cg21103227	0,550404361	0,915761217	-0,37	-0,724171942	7,43E-24	8,35E-23	Yes	Yes	Hypomethylated in HCC
WNT7A	List_3	cg23655615	0,53014866	0,864194864	-0,33	-0,694598381	8,64E-24	9,64E-23	Yes	Yes	Hypomethylated in HCC
LITAF	List_	cg08767044	0,37741795	0,72630788	-0,35	-	1,53E-23	1,67E-22	Yes	Yes	Hypomethylated

	2					0,926418341					in HCC
HK3	List_ 3	cg04875020	0,535950744	0,806599021	-0,27	-0,58085704	1,62E-23	1,76E-22	Yes	Yes	Hypomethylated in HCC
CAMTA1	List_ 1	cg21583016	0,421635047	0,773786036	-0,35	-0,86064786	2,50E-23	2,66E-22	Yes	Yes	Hypomethylated in HCC
DNAH17	List_ 2	cg25730791	0,52515205	0,880586623	-0,36	- 0,734807087	3,21E-23	3,38E-22	Yes	Yes	Hypomethylated in HCC
DNAH17	List_ 2	cg00461299	0,508637152	0,837538481	-0,33	- 0,708553317	1,40E-22	1,37E-21	Yes	Yes	Hypomethylated in HCC
WNT7A	List_ 3	cg21224025	0,539480607	0,8661206	-0,33	- 0,673060911	1,49E-22	1,47E-21	Yes	Yes	Hypomethylated in HCC
WNT7A	List_ 3	cg10460033	0,221803999	0,477416134	-0,26	- 1,072248549	1,97E-22	1,91E-21	Yes	Yes	Hypomethylated in HCC
DNAH17	List_ 2	cg10332979	0,474277816	0,870048587	-0,40	- 0,861748258	2,49E-22	2,39E-21	Yes	Yes	Hypomethylated in HCC
EGFLAM	List_ 3	cg08264481	0,46570847	0,819064781	-0,35	- 0,801407121	3,20E-21	2,73E-20	Yes	Yes	Hypomethylated in HCC
DNAH17	List_ 2	cg10217661	0,566533023	0,907440883	-0,34	- 0,670211952	5,42E-21	4,51E-20	Yes	Yes	Hypomethylated in HCC
CAMTA1	List_ 1	cg12138124	0,539106196	0,919118307	-0,38	- 0,758777119	3,31E-20	2,54E-19	Yes	Yes	Hypomethylated in HCC
DNAH17	List_ 2	cg05361750	0,572059338	0,91657028	-0,34	- 0,670734173	4,18E-20	3,17E-19	Yes	Yes	Hypomethylated in HCC
HLA-DPB2	List_ 2	cg15019001	0,487527695	0,81390218	-0,33	- 0,727696225	4,66E-20	3,51E-19	Yes	Yes	Hypomethylated in HCC
CAMTA1	List_ 1	cg06082897	0,572077438	0,901284218	-0,33	- 0,646689984	5,09E-20	3,82E-19	Yes	Yes	Hypomethylated in HCC
EGFLAM	List_ 3	cg18855621	0,524367615	0,816333923	-0,29	- 0,628892318	5,89E-20	4,39E-19	Yes	Yes	Hypomethylated in HCC
DNAH17	List_	cg16678718	0,655016462	0,934843996	-0,28	-	6,88E-20	5,10E-19	Yes	Yes	Hypomethylated

	2					0,506686091					in HCC
HLA-DPB2	List_ 2	cg11786476	0,412696491	0,733545236	-0,32	- 0,814798381	2,12E-19	1,49E-18	Yes	Yes	Hypomethylated in HCC
CAMTA1	List_ 1	cg07008478	0,548488103	0,881201873	-0,33	- 0,674225716	2,15E-19	1,51E-18	Yes	Yes	Hypomethylated in HCC
CAMTA1	List_ 1	cg00911446	0,64609296	0,950942104	-0,30	- 0,550549271	4,53E-19	3,08E-18	Yes	Yes	Hypomethylated in HCC
ZNF366	List_ 3	cg00657287	0,662304357	0,940257717	-0,28	- 0,499204337	6,22E-19	4,18E-18	Yes	Yes	Hypomethylated in HCC
CAMTA1	List_ 1	cg03571764	0,58087236	0,880783922	-0,30	- 0,592229007	3,09E-18	1,93E-17	Yes	Yes	Hypomethylated in HCC
CAMTA1	List_ 1	cg21942218	0,709992882	0,967412506	-0,26	-0,44098492	3,59E-18	2,23E-17	Yes	Yes	Hypomethylated in HCC
DNAH17	List_ 2	cg20723425	0,600003866	0,908066883	-0,31	- 0,589780876	4,14E-18	2,55E-17	Yes	Yes	Hypomethylated in HCC
CAMTA1	List_ 1	cg25196088	0,567670094	0,868398765	-0,30	- 0,604630219	2,38E-17	1,36E-16	Yes	Yes	Hypomethylated in HCC
DNAH17	List_ 2	cg09577144	0,508213014	0,745553346	-0,24	- 0,543988375	6,68E-17	3,63E-16	Yes	Yes	Hypomethylated in HCC
DNAH17	List_ 2	cg00235657	0,535186818	0,905927299	-0,37	- 0,748482412	7,42E-17	4,02E-16	Yes	Yes	Hypomethylated in HCC
DNAH17	List_ 2	cg20690714	0,597755487	0,922732124	-0,32	- 0,617971792	7,89E-17	4,26E-16	Yes	Yes	Hypomethylated in HCC
DNAH17	List_ 2	cg00249503	0,569669839	0,87864422	-0,31	-0,61637451	1,28E-16	6,79E-16	Yes	Yes	Hypomethylated in HCC
CAMTA1	List_ 1	cg06800235	0,343503347	0,640900093	-0,30	- 0,880712244	1,87E-16	9,72E-16	Yes	Yes	Hypomethylated in HCC
CAMTA1	List_ 1	cg00808305	0,280769908	0,563846296	-0,28	- 0,980786394	2,14E-16	1,10E-15	Yes	Yes	Hypomethylated in HCC
PTPRC	List_	cg26399994	0,603438957	0,900290235	-0,30	-	6,83E-16	3,36E-15	Yes	Yes	Hypomethylated

	1					0,569406814					in HCC
DNAH17	List_ 2	cg17514088	0,466747917	0,778802439	-0,31	- 0,726437377	7,19E-16	3,52E-15	Yes	Yes	Hypomethylated in HCC
DNAH17	List_ 2	cg14927663	0,59178536	0,932090945	-0,34	- 0,646617328	1,67E-15	7,90E-15	Yes	Yes	Hypomethylated in HCC
ZNF366	List_ 3	cg11905892	0,638268465	0,93561169	-0,30	- 0,544656476	1,43E-14	6,14E-14	Yes	Yes	Hypomethylated in HCC
FAM189A1	List_ 3	cg25941083	0,382626383	0,602034132	-0,22	- 0,640454994	2,58E-14	1,08E-13	Yes	Yes	Hypomethylated in HCC
CAMTA1	List_ 1	cg04210471	0,635847272	0,90986939	-0,27	-0,51023599	5,06E-14	2,06E-13	Yes	Yes	Hypomethylated in HCC
CAMTA1	List_ 1	cg10536786	0,437267224	0,695212755	-0,26	- 0,656921526	2,16E-13	8,27E-13	Yes	Yes	Hypomethylated in HCC
GAS7	List_ 3	cg02605292	0,58206587	0,876365564	-0,29	- 0,582144143	5,06E-13	1,87E-12	Yes	Yes	Hypomethylated in HCC
CCR5	List_ 2	cg22984586	0,192909335	0,38874862	-0,20	- 0,974644285	9,10E-13	3,28E-12	Yes	Yes	Hypomethylated in HCC
PIK3CG	List_ 3	cg00604356	0,321812424	0,562223201	-0,24	- 0,786210087	7,69E-12	2,54E-11	Yes	Yes	Hypomethylated in HCC
ITGA11	List_ 3	cg08872353	0,530051459	0,73186379	-0,20	- 0,458057445	6,54E-09	1,65E-08	Yes	Yes	Hypomethylated in HCC
GAS7	List_ 3	cg06130714	0,480093765	0,685119818	-0,21	- 0,504203884	3,02E-08	7,16E-08	Yes	Yes	Hypomethylated in HCC
CAMTA1	List_ 1	cg12661316	0,558753034	0,770590196	-0,21	- 0,456763007	8,47E-08	1,93E-07	Yes	Yes	Hypomethylated in HCC
CAMTA1	List_ 1	cg17081408	0,521348459	0,78588637	-0,26	- 0,582904176	3,22E-07	6,94E-07	Yes	Yes	Hypomethylated in HCC
ZNF366	List_ 3	cg22558265	0,695090957	0,907243944	-0,21	- 0,379496095	5,62E-07	1,19E-06	Yes	Yes	Hypomethylated in HCC
SORBS1	List_	cg27111150	0,710314508	0,354447604	0,36	0,982915545	2,47E-89	4,21E-85	Yes	Yes	Hypermethylated

	3										in HCC
EXOC2	List_ 3	cg04789318	0,931068733	0,735436413	0,20	0,336214803	8,55E-77	2,10E-73	Yes	Yes	Hypermethylated in HCC
CHERP	List_ 3	cg07688052	0,938035206	0,710800503	0,23	0,395340619	1,63E-69	1,71E-66	Yes	Yes	Hypermethylated in HCC
CCDC40	List_ 2	cg08109808	0,814395144	0,417772738	0,40	0,946491464	1,45E-67	1,20E-64	Yes	Yes	Hypermethylated in HCC
PCNX	List_ 3	cg14009504	0,854931506	0,517316203	0,34	0,713917561	1,26E-63	7,08E-61	Yes	Yes	Hypermethylated in HCC
TNS1	List_ 3	cg09548780	0,601948096	0,135358361	0,47	2,073795237	6,94E-63	3,60E-60	Yes	Yes	Hypermethylated in HCC
SATB2	List_ 1	cg20785796	0,717585674	0,30442733	0,41	1,210390516	4,33E-55	9,91E-53	Yes	Yes	Hypermethylated in HCC
ZNF212	List_ 1	cg05476998	0,888560757	0,646782788	0,24	0,452199737	3,79E-54	7,97E-52	Yes	Yes	Hypermethylated in HCC
SATB2	List_ 1	cg23994043	0,618555001	0,235704173	0,38	1,35511663	1,95E-53	3,84E-51	Yes	Yes	Hypermethylated in HCC
TSC2	List_ 3	cg02364279	0,822441921	0,55074774	0,27	0,569997719	2,03E-53	4,00E-51	Yes	Yes	Hypermethylated in HCC
TNS1	List_ 3	cg06320380	0,611725925	0,242981103	0,37	1,297249116	2,76E-53	5,34E-51	Yes	Yes	Hypermethylated in HCC
FBRSL1	List_ 1	cg16719582	0,823837672	0,519785365	0,30	0,654358562	2,79E-53	5,39E-51	Yes	Yes	Hypermethylated in HCC
CCDC84	List_ 3	cg27211899	0,81860105	0,479364003	0,34	0,759769665	2,04E-52	3,62E-50	Yes	Yes	Hypermethylated in HCC
TSC2	List_ 3	cg06330323	0,690211769	0,266269106	0,42	1,341717067	2,78E-51	4,42E-49	Yes	Yes	Hypermethylated in HCC
PDE4DIP	List_ 2	cg19084726	0,457368622	0,106526394	0,35	2,003904109	9,04E-51	1,38E-48	Yes	Yes	Hypermethylated in HCC
C1QTNF4	List_	cg05537653	0,794568195	0,515360672	0,28	0,614906504	5,18E-50	7,36E-48	Yes	Yes	Hypermethylated

	3										in HCC
KCNS2	List_ 3	cg14486338	0,665524873	0,218343544	0,45	1,564802889	1,09E-49	1,50E-47	Yes	Yes	Hypermethylated in HCC
ZNF876P	List_ 2	cg20296343	0,644563286	0,234121024	0,41	1,42293614	3,67E-46	3,65E-44	Yes	Yes	Hypermethylated in HCC
KAT2A	List_ 3	cg16550651	0,849765267	0,520275486	0,33	0,697200774	8,13E-45	7,14E-43	Yes	Yes	Hypermethylated in HCC
SWAP70	List_ 3	cg08213398	0,809485469	0,570267712	0,24	0,497999713	1,55E-44	1,33E-42	Yes	Yes	Hypermethylated in HCC
C1QTNF4	List_ 3	cg18356785	0,689352312	0,36150899	0,33	0,912622299	1,21E-43	9,53E-42	Yes	Yes	Hypermethylated in HCC
ZNF540	List_ 2	cg03975694	0,55674679	0,177508539	0,38	1,595748017	1,70E-40	1,00E-38	Yes	Yes	Hypermethylated in HCC
MICA	List_ 3	cg23826579	0,826849097	0,599514324	0,23	0,45730737	5,89E-40	3,29E-38	Yes	Yes	Hypermethylated in HCC
ZNF876P	List_ 2	cg12547166	0,620881746	0,271624512	0,35	1,163596702	8,58E-40	4,72E-38	Yes	Yes	Hypermethylated in HCC
STX1A	List_ 1	cg02610600	0,752121494	0,480087484	0,27	0,6369817	5,76E-39	2,93E-37	Yes	Yes	Hypermethylated in HCC
CRYBB3	List_ 3	cg19288514	0,782474951	0,543581519	0,24	0,517569554	8,53E-39	4,28E-37	Yes	Yes	Hypermethylated in HCC
SKI	List_ 3	cg12483545	0,774560615	0,430818806	0,34	0,831699141	1,54E-38	7,55E-37	Yes	Yes	Hypermethylated in HCC
MYH14	List_ 3	cg11992783	0,839163187	0,633821173	0,21	0,399381805	2,41E-38	1,16E-36	Yes	Yes	Hypermethylated in HCC
NSD1	List_ 1	cg19731612	0,820886314	0,582441136	0,24	0,487979277	7,77E-38	3,56E-36	Yes	Yes	Hypermethylated in HCC
NSD1	List_ 1	cg18121224	0,798646384	0,517649148	0,28	0,61593001	1,20E-37	5,41E-36	Yes	Yes	Hypermethylated in HCC
PIAS1	List_	cg05105016	0,810493124	0,600084759	0,21	0,427481553	1,11E-35	4,12E-34	Yes	Yes	Hypermethylated

	1										in HCC
TSC2	List_ 3	cg02504384	0,713408243	0,478814879	0,23	0,565521837	1,14E-34	3,86E-33	Yes	Yes	Hypermethylated in HCC
SATB2	List_ 1	cg03163783	0,705856705	0,350653797	0,36	0,989056217	1,84E-33	5,55E-32	Yes	Yes	Hypermethylated in HCC
CREBBP	List_ 3	cg03368634	0,806100634	0,547139182	0,26	0,550709285	1,19E-29	2,44E-28	Yes	Yes	Hypermethylated in HCC
BMP8A	List_ 1	cg11763509	0,610124936	0,356974472	0,25	0,756879194	2,39E-29	4,75E-28	Yes	Yes	Hypermethylated in HCC
ZIC5	List_ 1	cg00529958	0,418183969	0,130392192	0,29	1,608768079	1,29E-28	2,37E-27	Yes	Yes	Hypermethylated in HCC
TSC2	List_ 3	cg06094085	0,798182307	0,584677802	0,21	0,442572545	1,34E-28	2,46E-27	Yes	Yes	Hypermethylated in HCC
KCNK7	List_ 2	cg01178624	0,760506407	0,475527623	0,28	0,666253578	1,18E-26	1,78E-25	Yes	Yes	Hypermethylated in HCC
NSD1	List_ 1	cg08369368	0,389943673	0,0667812	0,32	2,380971817	1,53E-26	2,28E-25	Yes	Yes	Hypermethylated in HCC
KIAA1875	List_ 3	cg01423393	0,704929759	0,50345437	0,20	0,477565433	3,61E-25	4,65E-24	Yes	Yes	Hypermethylated in HCC
NID1	List_ 2	cg18765906	0,655432968	0,311083841	0,34	1,051343284	1,07E-24	1,31E-23	Yes	Yes	Hypermethylated in HCC
B3GNT9	List_ 2	cg05333146	0,775671284	0,519994988	0,26	0,567947115	1,63E-24	1,97E-23	Yes	Yes	Hypermethylated in HCC
NRP2	List_ 3	cg17455088	0,605659829	0,284969232	0,32	1,061568961	1,96E-24	2,34E-23	Yes	Yes	Hypermethylated in HCC
RNF220	List_ 3	cg09860921	0,734378346	0,519913671	0,21	0,49027874	6,64E-24	7,50E-23	Yes	Yes	Hypermethylated in HCC
CNKSR1	List_ 3	cg17330765	0,77744123	0,557484073	0,22	0,47259235	1,51E-22	1,48E-21	Yes	Yes	Hypermethylated in HCC
NSD1	List_	cg18016826	0,397282969	0,076097884	0,32	2,2419818	4,46E-22	4,17E-21	Yes	Yes	Hypermethylated

	1										in HCC
ZNF876P	List_2	cg23063647	0,673270819	0,389906354	0,28	0,772795312	1,00E-21	9,04E-21	Yes	Yes	Hypermethylated in HCC
RNF220	List_3	cg01422881	0,596173945	0,344472108	0,25	0,774059731	3,06E-21	2,61E-20	Yes	Yes	Hypermethylated in HCC
KCNS2	List_3	cg11964564	0,346491116	0,068382504	0,28	2,185262564	5,28E-19	3,57E-18	Yes	Yes	Hypermethylated in HCC
ZIC5	List_1	cg20985450	0,488580889	0,129077099	0,36	1,841942709	6,27E-19	4,21E-18	Yes	Yes	Hypermethylated in HCC
KRTCAP3	List_3	cg17158414	0,740277812	0,511463141	0,23	0,524859614	3,06E-18	1,91E-17	Yes	Yes	Hypermethylated in HCC
CUL3	List_3	cg01474011	0,764520444	0,556137474	0,21	0,452150898	5,94E-18	3,60E-17	Yes	Yes	Hypermethylated in HCC
ZIC5	List_1	cg11077516	0,599715291	0,320782721	0,28	0,882251853	1,29E-17	7,57E-17	Yes	Yes	Hypermethylated in HCC
ZIC5	List_1	cg17930361	0,345200547	0,087500019	0,26	1,865159401	7,85E-17	4,24E-16	Yes	Yes	Hypermethylated in HCC
THRSP	List_3	cg03493668	0,736262396	0,488954783	0,25	0,580773911	9,86E-17	5,27E-16	Yes	Yes	Hypermethylated in HCC
KRTCAP3	List_3	cg02592271	0,721665637	0,480438096	0,24	0,577113456	2,38E-16	1,22E-15	Yes	Yes	Hypermethylated in HCC
KRTCAP3	List_3	cg24768116	0,690346477	0,425956627	0,26	0,683884223	6,24E-16	3,08E-15	Yes	Yes	Hypermethylated in HCC
CLEC14A	List_3	cg05057720	0,487230336	0,161023624	0,33	1,539718694	1,48E-15	7,05E-15	Yes	Yes	Hypermethylated in HCC
KRTCAP3	List_3	cg11618577	0,668759807	0,444573698	0,22	0,578386928	1,09E-14	4,74E-14	Yes	Yes	Hypermethylated in HCC
TMEM18	List_3	cg27237671	0,678177853	0,481159473	0,20	0,486589938	1,11E-14	4,81E-14	Yes	Yes	Hypermethylated in HCC
NRP2	List_	cg10648139	0,453029416	0,139796015	0,31	1,628104602	4,18E-14	1,72E-13	Yes	Yes	Hypermethylated

	3										in HCC
KCNS2	List_ 3	cg14688104	0,340469027	0,051581203	0,29	2,508724992	1,15E-13	4,53E-13	Yes	Yes	Hypermethylated in HCC
ZIC5	List_ 1	cg03313945	0,408715413	0,126902156	0,28	1,612824854	3,24E-13	1,22E-12	Yes	Yes	Hypermethylated in HCC
C1QTNF4	List_ 3	cg17282004	0,498449663	0,293001854	0,21	0,746778334	7,82E-13	2,84E-12	Yes	Yes	Hypermethylated in HCC
CAMTA1	List_ 1	cg00783553	0,338542896	0,11602324	0,22	1,467646413	5,87E-12	1,96E-11	Yes	Yes	Hypermethylated in HCC
ITGB3	List_ 3	cg03460756	0,548670946	0,315251923	0,23	0,780441206	1,14E-11	3,72E-11	Yes	Yes	Hypermethylated in HCC
CLEC14A	List_ 3	cg16404157	0,520833052	0,236168222	0,28	1,108613667	2,07E-11	6,55E-11	Yes	Yes	Hypermethylated in HCC
NRP2	List_ 3	cg22367989	0,264060408	0,067335986	0,20	1,825282126	4,24E-10	1,19E-09	Yes	Yes	Hypermethylated in HCC
CRHBP	List_ 3	cg01071966	0,504392151	0,290180216	0,21	0,777039731	1,02E-09	2,76E-09	Yes	Yes	Hypermethylated in HCC
SIPA1L1	List_ 3	cg02058870	0,615702868	0,380122038	0,24	0,681552218	2,73E-09	7,11E-09	Yes	Yes	Hypermethylated in HCC
B3GNT9	List_ 2	cg06279276	0,378888095	0,162566599	0,22	1,172201818	3,28E-09	8,49E-09	Yes	Yes	Hypermethylated in HCC
ZIC5	List_ 1	cg10679688	0,440296176	0,215658561	0,22	0,996732713	7,71E-09	1,93E-08	Yes	Yes	Hypermethylated in HCC
ITGAM	List_ 3	cg02256631	0,373541446	0,156235655	0,22	1,206152639	4,50E-08	1,05E-07	Yes	Yes	Hypermethylated in HCC
KCNS2	List_ 3	cg08706670	0,298003746	0,092566222	0,21	1,586392218	9,48E-08	2,15E-07	Yes	Yes	Hypermethylated in HCC
CAMTA1	List_ 1	cg08640609	0,260589976	0,052173929	0,21	2,121726745	1,01E-07	2,28E-07	Yes	Yes	Hypermethylated in HCC
FAM189A1	List_	cg04283162	0,338508981	0,110882162	0,23	1,52759447	4,99E-07	1,06E-06	Yes	Yes	Hypermethylated

	3										in HCC
FNDC1	List_ 3	cg00157796	0,370065262	0,158200882	0,21	1,176061894	4,35E-05	7,70E-05	Yes	Yes	Hypermethylated in HCC
CHST11	List_ 3	cg22260952	0,298001159	0,099722053	0,20	1,48908226	0,000180 824	0,000301 492	Yes	Yes	Hypermethylated in HCC
ZYG11A	List_ 3	cg20287790	0,934964064	0,755239011	0,18	0,304349044	3,00E-60	1,18E-57	No	Yes	-
STX3	List_ 3	cg14333691	0,84084134	0,658948574	0,18	0,346994823	2,86E-47	3,16E-45	No	Yes	-
TSC2	List_ 3	cg07730183	0,906570667	0,736849713	0,17	0,295428154	1,04E-44	9,08E-43	No	Yes	-
USP34	List_ 1	cg01145124	0,945206472	0,804028251	0,14	0,230733748	2,08E-43	1,61E-41	No	Yes	-
PURA	List_ 2	cg21778810	0,051399191	0,110706167	-0,06	- 0,975207847	2,75E-43	2,10E-41	No	Yes	-
TIGD1	List_ 3	cg18346402	0,884184946	0,701563923	0,18	0,329579892	3,02E-37	1,31E-35	No	Yes	-
MCM6	List_ 3	cg11446240	0,100837107	0,227701444	-0,13	- 1,100709706	3,20E-34	1,03E-32	No	Yes	-
ATG4B	List_ 3	cg08969328	0,834563402	0,691109919	0,14	0,268565086	1,28E-32	3,56E-31	No	Yes	-
HIPK1	List_ 3	cg17588904	0,738368521	0,574953476	0,16	0,355426991	1,25E-27	2,08E-26	No	Yes	-
COPA	List_ 3	cg09866659	0,731941546	0,558522958	0,17	0,38408692	3,41E-27	5,43E-26	No	Yes	-
SMARCC1	List_ 2	cg19134770	0,871949202	0,751782304	0,12	0,211316787	3,43E-23	3,60E-22	No	Yes	-
ATP6V1C1	List_ 3	cg03506193	0,117463086	0,232190326	-0,11	- 0,926061747	4,02E-23	4,19E-22	No	Yes	-
SCAMP1	List_	cg03056766	0,047314261	0,088288621	-0,04	-	4,75E-21	3,98E-20	No	Yes	-

	2					0,778130248					
KRTCAP3	List_ 3	cg04845466	0,809672269	0,626041083	0,18	0,365927234	2,02E-19	1,43E-18	No	Yes	-
RWDD1	List_ 1	cg23280258	0,798702495	0,65202233	0,15	0,288729182	2,55E-19	1,78E-18	No	Yes	-
SIPA1L1	List_ 3	cg15378445	0,849480726	0,74420309	0,11	0,188512227	3,59E-18	2,23E-17	No	Yes	-
SPG11	List_ 1	cg12228919	0,055413	0,088353072	-0,03	-0,588392733	2,83E-17	1,60E-16	No	Yes	-
WDR82	List_ 1	cg24007312	0,085243635	0,13562583	-0,05	-0,612571681	9,78E-17	5,23E-16	No	Yes	-
MATN2	List_ 3	cg19987349	0,825631473	0,746836379	0,08	0,142885394	2,47E-16	1,27E-15	No	Yes	-
MAST2	List_ 3	cg02835462	0,75448421	0,565267608	0,19	0,410253465	5,16E-16	2,56E-15	No	Yes	-
ATP11A	List_ 2	cg25142327	0,90869295	0,836702534	0,07	0,117727552	6,18E-16	3,05E-15	No	Yes	-
C9orf3	List_ 3	cg14375632	0,780249875	0,606065739	0,17	0,359224596	1,94E-14	8,22E-14	No	Yes	-
LITAF	List_ 2	cg07994696	0,245123056	0,367814378	-0,12	-0,56648432	2,34E-13	8,92E-13	No	Yes	-
EGFLAM	List_ 3	cg25625968	0,227390933	0,372038342	-0,14	-0,686452601	3,27E-13	1,23E-12	No	Yes	-
REEP3	List_ 3	cg10300729	0,846646079	0,737493761	0,11	0,196637744	3,76E-13	1,41E-12	No	Yes	-
CAMTA1	List_ 1	cg25763306	0,224250829	0,377114345	-0,15	-0,724705617	4,47E-13	1,66E-12	No	Yes	-
DNASE1L2	List_ 3	cg06235653	0,267645994	0,085694524	0,18	1,536738307	5,57E-13	2,05E-12	No	Yes	-
BLOC1S1	List_	cg12926596	0,622507743	0,80660363	-0,18	-0,36855284	7,61E-13	2,76E-12	No	Yes	-

	2										
MAP4	List_ 1	cg16509829	0,046585042	0,065199503	-0,02	- 0,410302385	9,24E-13	3,33E-12	No	Yes	-
KREMEN2	List_ 3	cg05169951	0,718138921	0,555005779	0,16	0,365948105	2,98E-12	1,02E-11	No	Yes	-
PPM1D	List_ 3	cg04180177	0,680597373	0,575556353	0,11	0,238036825	3,41E-12	1,16E-11	No	Yes	-
SLC25A27	List_ 3	cg19101566	0,745685061	0,584730673	0,16	0,345548618	8,91E-12	2,93E-11	No	Yes	-
UCN	List_ 3	cg01454215	0,238859715	0,078353474	0,16	1,493973936	1,22E-11	3,96E-11	No	Yes	-
PTPRJ	List_ 3	cg04462547	0,842334313	0,764846197	0,08	0,137509441	1,60E-11	5,12E-11	No	Yes	-
LHX3	List_ 4	cg14362758	0,236705782	0,09081312	0,15	1,291108125	7,90E-11	2,37E-10	No	Yes	-
KCNK7	List_ 2	cg13654525	0,74123877	0,618940086	0,12	0,25634893	1,68E-10	4,88E-10	No	Yes	-
ITGA11	List_ 3	cg24213777	0,546432215	0,730242923	-0,18	- 0,411792844	4,69E-10	1,31E-09	No	Yes	-
UCN	List_ 3	cg05113927	0,684324195	0,543460273	0,14	0,327129681	5,28E-10	1,47E-09	No	Yes	-
DNASE1L2	List_ 3	cg00249383	0,565807123	0,382333069	0,18	0,553506693	7,34E-10	2,02E-09	No	Yes	-
SPN	List_ 3	cg02030929	0,40367796	0,566807672	-0,16	- 0,479582257	2,86E-09	7,44E-09	No	Yes	-
PPAP2B	List_ 3	cg22396959	0,234977672	0,330506079	-0,10	- 0,475030292	4,26E-09	1,09E-08	No	Yes	-
ALDH4A1	List_ 3	cg12461099	0,696785944	0,565615784	0,13	0,296167196	7,29E-09	1,83E-08	No	Yes	-
GALK2	List_	cg00756450	0,925907947	0,89955543	0,03	0,041205076	1,61E-08	3,92E-08	No	Yes	-

	1										
STRN4	List_ 2	cg12254611	0,075840667	0,097755043	-0,02	- 0,328022205	2,44E-08	5,83E-08	No	Yes	-
FGFR4	List_ 3	cg12982374	0,677065318	0,541387018	0,14	0,317381959	2,54E-08	6,05E-08	No	Yes	-
CBFA2T3	List_ 3	cg27434245	0,284072086	0,101544065	0,18	1,398556099	4,14E-08	9,69E-08	No	Yes	-
FBRSL1	List_ 1	cg08818195	0,077274438	0,099616982	-0,02	- 0,328840242	6,27E-08	1,44E-07	No	Yes	-
CAMTA1	List_ 1	cg21144493	0,537033269	0,382300133	0,15	0,479670751	7,70E-08	1,76E-07	No	Yes	-
KLHL5	List_ 3	cg08217447	0,684486869	0,558404238	0,13	0,289030105	1,52E-07	3,37E-07	No	Yes	-
ZNF876P	List_ 2	cg18005867	0,431292247	0,246811795	0,18	0,781022935	1,79E-07	3,95E-07	No	Yes	-
PAQR6	List_ 3	cg24152297	0,847531709	0,810614354	0,04	0,06348563	2,24E-07	4,90E-07	No	Yes	-
STX1A	List_ 1	cg01804343	0,061146318	0,076623107	-0,02	- 0,283962829	2,66E-07	5,78E-07	No	Yes	-
UGT2B15	List_ 2	cg09189601	0,662704734	0,544713026	0,12	0,278231806	2,84E-07	6,17E-07	No	Yes	-
CCR5	List_ 2	cg00803692	0,637517069	0,811068312	-0,17	- 0,342584035	4,97E-07	1,05E-06	No	Yes	-
ANO10	List_ 1	cg11035303	0,062528227	0,115899744	-0,05	- 0,795660861	6,00E-07	1,26E-06	No	Yes	-
LPP	List_ 3	cg04423294	0,66720779	0,562807848	0,10	0,24154731	7,07E-07	1,48E-06	No	Yes	-
LPP	List_ 3	cg24454374	0,460828831	0,59085185	-0,13	- 0,351806648	1,35E-06	2,75E-06	No	Yes	-
ZDHHC7	List_	cg03655147	0,812896754	0,757532365	0,06	0,100483846	1,46E-06	2,97E-06	No	Yes	-

	3										
WDR82	List_ 1	cg11442381	0,08479497	0,069998077	0,01	0,244845187	1,59E-06	3,23E-06	No	Yes	-
ZMIZ2	List_ 3	cg11425656	0,702789203	0,607282664	0,10	0,207544209	2,22E-06	4,44E-06	No	Yes	-
PTPRJ	List_ 3	cg06298729	0,79510556	0,732521102	0,06	0,116745925	3,14E-06	6,18E-06	No	Yes	-
DNHD1	List_ 3	cg10383568	0,579499761	0,420951412	0,16	0,451966007	4,27E-06	8,30E-06	No	Yes	-
ALDH4A1	List_ 3	cg22390041	0,430733428	0,55383528	-0,12	- 0,355367429	4,81E-06	9,32E-06	No	Yes	-
CCDC57	List_ 3	cg22142205	0,817943387	0,77065436	0,05	0,084848195	5,53E-06	1,07E-05	No	Yes	-
NAA30	List_ 3	cg03318573	0,619499362	0,502407094	0,12	0,296914465	5,94E-06	1,14E-05	No	Yes	-
CHST11	List_ 3	cg16861964	0,491036366	0,624053302	-0,13	- 0,339688805	6,58E-06	1,26E-05	No	Yes	-
CCDC57	List_ 3	cg12879038	0,71298492	0,625580394	0,09	0,185890933	7,38E-06	1,41E-05	No	Yes	-
FAM189A1	List_ 3	cg13942157	0,249280407	0,084682412	0,16	1,453344822	7,62E-06	1,45E-05	No	Yes	-
PDE4DIP	List_ 2	cg15743907	0,58259746	0,501865782	0,08	0,211286878	8,30E-06	1,57E-05	No	Yes	-
IRX3	List_ 3	cg05001964	0,238524117	0,091124563	0,15	1,297252384	1,10E-05	2,05E-05	No	Yes	-
CYP1A2	List_ 3	cg04968473	0,775359789	0,698272751	0,08	0,149048693	1,38E-05	2,57E-05	No	Yes	-
CAMTA1	List_ 1	cg22488970	0,271708519	0,08786264	0,18	1,525373086	1,70E-05	3,13E-05	No	Yes	-
CHST11	List_	cg22827210	0,501172514	0,634660128	-0,13	-	1,73E-05	3,18E-05	No	Yes	-

	3					0,334728492					
CBFA2T3	List_3	cg00762678	0,396770224	0,518248868	-0,12	-0,377003695	2,12E-05	3,87E-05	No	Yes	-
GLTSCR1	List_3	cg22461472	0,789181993	0,728576133	0,06	0,113777433	2,28E-05	4,15E-05	No	Yes	-
FNDC1	List_3	cg09107912	0,231033585	0,090726663	0,14	1,258788554	3,11E-05	5,57E-05	No	Yes	-
FBRSL1	List_1	cg00370303	0,175111528	0,046924432	0,13	1,701274843	4,12E-05	7,31E-05	No	Yes	-
CHST11	List_3	cg07696842	0,411900814	0,539050524	-0,13	-0,380035041	4,46E-05	7,89E-05	No	Yes	-
SAMD11	List_1	cg13904806	0,806970355	0,753449271	0,05	0,097751432	5,33E-05	9,35E-05	No	Yes	-
PALM3	List_3	cg11437328	0,704570148	0,629656393	0,07	0,15977851	5,62E-05	9,84E-05	No	Yes	-
RGS10	List_3	cg10200202	0,484133499	0,61126636	-0,13	-0,330311074	7,13E-05	0,000123564	No	Yes	-
IRX3	List_3	cg08279075	0,241664996	0,09718506	0,14	1,231400744	8,98E-05	0,000154172	No	Yes	-
FAM20B	List_3	cg24997888	0,734488844	0,667123361	0,07	0,136831537	0,000139666	0,000235432	No	Yes	-
SPN	List_3	cg09946623	0,603132479	0,701337782	-0,10	-0,214335962	0,000170225	0,000284571	No	Yes	-
FGFR4	List_3	cg17386911	0,738271303	0,675536848	0,06	0,126327233	0,000402984	0,000648709	No	Yes	-
STX1A	List_1	cg27469719	0,080714714	0,090007628	-0,01	-0,140701564	0,0007374	0,001156	No	Yes	-
C9orf3	List_3	cg13853813	0,765679127	0,724360152	0,04	0,078972204	0,000829148	0,001292617	No	Yes	-
CMYA5	List_	cg11438310	0,400024305	0,287666654	0,11	0,46201182	0,000964	0,001493	No	Yes	-

	3						87	756			
CMYA5	List_3	cg00611789	0,381601211	0,255300263	0,13	0,561759122	0,001000507	0,001546422	No	Yes	-
LHX3	List_4	cg13658899	0,294051805	0,18921953	0,10	0,609958069	0,00101839	0,001572882	No	Yes	-
PRMT7	List_3	cg10061770	0,379548112	0,271422857	0,11	0,469062012	0,001189039	0,001823627	No	Yes	-
THSD7A	List_2	cg24676244	0,444887208	0,313012576	0,13	0,493918528	0,001222784	0,001872902	No	Yes	-
THSD7A	List_2	cg01797590	0,435090155	0,358769562	0,08	0,271378008	0,001384416	0,002108615	No	Yes	-
PIK3CG	List_3	cg08779777	0,482179727	0,608867896	-0,13	-0,330446249	0,001956797	0,002933013	No	Yes	-
PRR5	List_3	cg04607412	0,333140508	0,245522648	0,09	0,425348275	0,002986073	0,0043855	No	Yes	-
SAMD11	List_1	cg24362661	0,384893124	0,445312037	-0,06	-0,205393354	0,003121916	0,004575409	No	Yes	-
CMYA5	List_3	cg09481121	0,517968506	0,430976715	0,09	0,259749395	0,003350236	0,0048941	No	Yes	-
RGS10	List_3	cg17527393	0,098841093	0,03866792	0,06	1,161180332	0,004141802	0,005988643	No	Yes	-
FBRSL1	List_1	cg23890800	0,156300781	0,053381762	0,10	1,391655274	0,004435664	0,006392837	No	Yes	-
KREMEN2	List_3	cg26494929	0,285783001	0,169539942	0,12	0,720234309	0,006084779	0,008632012	No	Yes	-
PPP2R5C	List_1	cg09990596	0,110293027	0,120966262	-0,01	-0,122642193	0,006451792	0,009125493	No	Yes	-
C9orf3	List_3	cg14582550	0,281388285	0,321934677	-0,04	-0,18795648	0,010022894	0,013862182	No	Yes	-
FBRSL1	List_	cg18450555	0,09819856	0,037030815	0,06	1,202003056	0,010297	0,014221	No	Yes	-

	1						573	899			
THSD7A	List_<br>2	cg15090509	0,428918927	0,31353001	0,12	0,44005494	0,012357<br>248	0,016905<br>601	No	Yes	-
DDX51	List_<br>2	cg22672078	0,086236498	0,092912511	-0,01	-<br>0,096762329	0,016327<br>532	0,022027<br>764	No	Yes	-
RGS10	List_<br>3	cg19653161	0,23822419	0,116597769	0,12	0,971391739	0,025792<br>51	0,033949<br>027	No	Yes	-
PPAP2B	List_<br>3	cg10500503	0,570658577	0,508719921	0,06	0,162734342	0,026310<br>633	0,034594<br>586	No	Yes	-
LITAF	List_<br>2	cg04359558	0,359541546	0,404629816	-0,05	-<br>0,166087296	0,02864	0,037482<br>837	No	Yes	-
WDR82	List_<br>1	cg12661343	0,175450533	0,166607087	0,01	0,070491176	0,030519<br>322	0,039812<br>767	No	Yes	-
SAMD11	List_<br>1	cg13546858	0,533338673	0,460411546	0,07	0,207928265	0,031289<br>444	0,040761<br>15	No	Yes	-
BMP8A	List_<br>1	cg25139493	0,429623415	0,293019734	0,14	0,536856479	0,035342<br>198	0,045716<br>989	No	Yes	-
CMYA5	List_<br>3	cg10257870	0,466611226	0,391738367	0,07	0,246556685	0,041062<br>483	0,052674<br>023	No	No	-
BMP8A	List_<br>1	cg02575697	0,171963608	0,069075359	0,10	1,202349838	0,047264<br>646	0,060155<br>53	No	No	-
CHST11	List_<br>3	cg01964337	0,313977459	0,340885791	-0,03	-<br>0,115108089	0,049892<br>245	0,063297<br>487	No	No	-
PTBP1	List_<br>3	cg19373090	0,053691067	0,057037706	0,00	-<br>0,073881729	0,060198<br>439	0,075545<br>817	No	No	-
CHST11	List_<br>3	cg11739675	0,18746699	0,091647088	0,10	0,958042614	0,064629<br>323	0,080770<br>696	No	No	-
PPP2R5C	List_<br>1	cg08163906	0,147848426	0,129742078	0,02	0,175773375	0,086224<br>674	0,105895<br>523	No	No	-
BMP8A	List_	cg15947940	0,110799571	0,032834169	0,08	1,495781316	0,104115	0,126367	No	No	-

	1						461	241			
BMP8A	List_1	cg08748615	0,18674844	0,069446696	0,12	1,308293069	0,117716184	0,141748674	No	No	-
CMYA5	List_3	cg03546977	0,387294396	0,323611978	0,06	0,252037355	0,125901456	0,150940087	No	No	-
SAMD11	List_1	cg02439789	0,551745553	0,509632956	0,04	0,112423867	0,132094969	0,157889466	No	No	-
SAMD11	List_1	cg03269716	0,527695586	0,565580403	-0,04	-0,098227844	0,155091397	0,183398294	No	No	-
ZNF366	List_3	cg04454664	0,342750098	0,361609364	-0,02	-0,07514037	0,200911877	0,233316648	No	No	-
NRP2	List_3	cg05348875	0,451493713	0,40719816	0,04	0,1455782	0,241125387	0,276288709	No	No	-
CHST11	List_3	cg17844339	0,753568373	0,848675162	-0,10	-0,169355113	0,247921306	0,283472256	No	No	-
SCAND3	List_3	cg22302929	0,602176632	0,59165482	0,01	0,02501195	0,269090876	0,305829879	No	No	-
SAMD11	List_1	cg13856810	0,501200269	0,530870609	-0,03	-0,08139491	0,319087367	0,357800247	No	No	-
ITGAM	List_3	cg22490695	0,660684825	0,704679984	-0,04	-0,091662423	0,320645016	0,359420226	No	No	-
LHX3	List_4	cg08967938	0,628067746	0,621223591	0,01	0,015558485	0,367495675	0,407401042	No	No	-
SAMD11	List_1	cg06531475	0,408476338	0,359276554	0,05	0,180444384	0,380042682	0,420152655	No	No	-
SAMD11	List_1	cg05527507	0,371538733	0,302086783	0,07	0,289882266	0,389513439	0,42971609	No	No	-
ITGA11	List_3	cg26217827	0,753100814	0,702906286	0,05	0,098161224	0,44713666	0,487513866	No	No	-
SKI	List_	cg12580943	0,406229153	0,371280077	0,03	0,126526867	0,470157	0,510339	No	No	-

	3						645	276			
HMBS	List_1	cg20929545	0,845081943	0,84798478	0,00	-0,004889375	0,476216269	0,516309808	No	No	-
PLOD3	List_1	cg18437077	0,186368161	0,187926203	0,00	-0,011401592	0,578519848	0,615784595	No	No	-
THSD7A	List_2	cg09557034	0,406291947	0,350981476	0,06	0,205670845	0,610119342	0,646037858	No	No	-
TNS1	List_3	cg12681370	0,320011323	0,290717118	0,03	0,134108532	0,733322863	0,761859288	No	No	-
PPP2R5C	List_1	cg15321108	0,101309763	0,096079549	0,01	0,069433596	0,757627553	0,784234175	No	No	-
CHST11	List_3	cg12529671	0,751832014	0,815446149	-0,06	-0,115701184	0,770132616	0,795735466	No	No	-
SAMD11	List_1	cg14324200	0,301379616	0,242303319	0,06	0,303515319	0,812883941	0,834699983	No	No	-
TXNDC5	List_3	cg11401394	0,555186425	0,578771979	-0,02	-0,058982199	0,862578154	0,879450809	No	No	-

Supplemental Table 10

Gene_Symbol	Expression HCC to Normal	p-value
ALDH4A1	Down	≤0.001
ANO10	Up	≤0.001
ATG4B	Up	≤0.001
ATP11A	Up	≤0.05
ATP6V0A1	Up	≤0.001
ATP6V1C1	Up	≤0.001
B3GNT9	Down	≤0.01
BLOC1S1	Up	≤0.05
BMP8A	Up	≤0.001
C1QTNF4	n.s.	-
C9orf3	n.s.	-
CAMTA1	Down	≤0.001
CBFA2T3	Down	≤0.001
CCDC40	Up	≤0.001
CCDC57	n.s.	-
CCDC84	Up	≤0.001
CCR5	Down	≤0.05
CHERP	n.s.	-
CHST11	n.s.	-
CLEC14A	Up	≤0.01
CMYA5	Down	≤0.001
CNKSR1	Down	≤0.001
COPA	Up	≤0.001
CREBBP	Down	≤0.05
CRHBP	Down	≤0.001
CRYBB3	n.s.	-
CUL3	Down	≤0.001
CYP1A2	Down	≤0.001
DDX51	Up	≤0.001
DNAH17	Up	≤0.001
DNASE1L2	Up	≤0.001
DNHD1	Up	≤0.001
EGFLAM	Up	≤0.01
EXOC2	Up	≤0.01
FAM189A1	Down	≤0.01
FAM20B	Up	≤0.001
FBRSL1	Up	≤0.001
FGFR4	Up	≤0.001
FNDC1	n.s.	-
GALK2	n.s.	-
GAS7	n.s.	-
GLTSCR1	Up	≤0.001
HIPK1	n.s.	-

HK3	Down	≤0.001
HLA-DPB2	Down	≤0.01
IRX3	Up	≤0.001
ITGA11	Up	≤0.001
ITGAM	n.s.	-
ITGB3	Down	≤0.001
KAT2A	Up	≤0.001
KCNK7	Up	≤0.01
KCNS2	n.s.	-
KIAA1875	Up	≤0.001
KLHL5	n.s.	-
KREMEN2	Up	≤0.001
KRTCAP3	Down	≤0.001
LHX3	n.s.	-
LITAF	n.s.	-
LPP	Down	≤0.01
LZTS1	Up	≤0.001
MAP4	Up	≤0.001
MAST2	Up	≤0.001
MATN2	Down	≤0.01
MCM6	Up	≤0.001
MICA	Up	≤0.001
MYH14	Up	≤0.001
NAA30	Down	≤0.001
NID1	Down	≤0.001
NRP2	Down	≤0.001
NSD1	Up	≤0.001
PALM3	Down	≤0.001
PAQR6	Up	≤0.001
PCNX	Down	≤0.001
PDE4DIP	n.s.	-
PIAS1	n.s.	-
PIK3CG	Down	≤0.01
PPAP2B	Down	≤0.001
PPM1D	n.s.	-
PPP2R5C	Down	≤0.01
PRMT7	Up	≤0.01
PRR5	Down	≤0.001
PTPRC	Down	≤0.001
PTPRJ	Up	≤0.01
PURA	Down	≤0.001
REEP3	Down	≤0.001
RGS10	Up	≤0.01
RNF220	Up	≤0.001
RWDD1	n.s.	-
SAMD11	Down	≤0.001
SATB2	Up	≤0.001

SCAMP1	n.s.	-
SIPA1L1	n.s.	-
SKI	Down	≤0.05
SLC25A27	n.s.	-
SMARCC1	Up	≤0.001
SORBS1	Down	≤0.001
SPG11	Down	≤0.05
SPN	Down	≤0.001
STRN4	Up	≤0.001
STX1A	Up	≤0.001
STX3	Up	≤0.05
SWAP70	Up	≤0.001
THRSP	Down	≤0.001
THSD7A	Up	≤0.01
TIGD1	Up	≤0.001
TMEM18	n.s.	-
TNS1	Down	≤0.001
TSC2	Up	≤0.001
UCN	Up	≤0.001
UGT2B15	Down	≤0.001
USP34	n.s.	-
WDR82	n.s.	-
WNT7A	Down	≤0.001
ZDHHC7	n.s.	-
ZIC5	Up	≤0.001
ZMIZ2	Up	≤0.001
ZNF212	Up	≤0.001
ZNF366	n.s.	-
ZNF540	n.s.	-
ZNF876P	Up	≤0.01
ZYG11A	n.s.	-

Supplemental Table 11

Gene_Symbol	Logrank p-value	Hazard Ratio (high)	p(HR)	n(high)	n(low)
ALDH4A1	0.56	0.9	0.55	182	182
ANO10	0.004	1.7	0.0043	181	181
ATG4B	0.003	1.7	0.0033	182	182
ATP11A	0.031	1.5	0.031	182	182
ATP6V0A1	0.034	1.5	0.035	182	182
ATP6V1C1	0.73	1.1	0.73	182	182
B3GNT9	0.047	1.4	0.048	182	182
BLOC1S1	0.74	1.1	0.75	182	182
BMP8A	0.045	1.4	0.046	172	173
C1QTNF4	0.51	1.1	0.5	165	177
C9orf3	0.5	0.89	0.5	182	182
CAMTA1	0.012	1.6	0.013	182	182
CBFA2T3	0.095	0.74	0.095	182	182
CCDC40	0.23	1.2	0.23	181	181
CCDC57	0.44	0.87	0.44	182	182
CCDC84	0.34	1.2	0.34	182	182
CCR5	0.022	0.67	0.023	182	182
CHERP	0.85	1.0	0.84	182	182
CHST11	0.4	1.2	0.4	181	180
CLEC14A	0.15	0.77	0.15	181	181
CMYA5	0.9	0.98	0.9	182	182
CNKSR1	0.78	1.1	0.77	180	174
COPA	0.025	1.5	0.026	182	182
CREBBP	0.8	0.96	0.8	182	182
CRHBP	0.12	0.76	0.12	182	182
CRYBB3	0.51	1.1	0.51	178	179
CUL3	0.096	1.3	0.098	182	182
CYP1A2	0.93	0.98	0.93	182	182
DDX51	0.12	1.3	0.13	182	182
DNAH17	0.086	1.4	0.087	179	175
DNASE1L2	0.45	0.87	0.45	176	180
DNHD1	0.92	0.98	0.93	182	182
EGFLAM	0.48	0.88	0.48	181	179
EXOC2	0.13	1.3	0.13	182	182
FAM189A1	0.54	1.1	0.54	152	178
FAM20B	0.21	1.3	0.21	181	181
FBRSL1	0.067	1.4	0.068	182	182
FGFR4	0.059	1.4	0.061	182	182
FNDC1	0.25	1.2	0.25	182	182
GALK2	0.78	0.95	0.78	182	182
GAS7	0.23	1.2	0.23	180	181
GLTSCR1	0.39	1.2	0.39	181	180
HIPK1	0.26	1.2	0.25	182	182
HK3	0.88	1.0	0.88	180	181

HLA-DPB2	0.17	0.78	0.17	179	181
IRX3	0.96	1.0	0.96	182	182
ITGA11	0.22	1.2	0.22	180	181
ITGAM	0.61	1.1	0.61	182	182
ITGB3	0.93	1.0	0.94	182	182
KAT2A	0.24	1.2	0.24	182	182
KCNK7	0.19	1.3	0.19	176	179
KCNS2	N/A	N/A	N/A	N/A	N/A
KIAA1875	0.39	1.2	0.39	174	180
KLHL5	0.079	1.4	0.079	182	182
KREMEN2	0.18	1.3	0.18	179	157
KRTCAP3	0.15	0.77	0.15	182	182
LHX3	0.46	0.87	0.46	172	174
LITAF	0.12	1.3	0.12	182	182
LPP	0.038	1.5	0.038	182	182
LZTS1	0.65	1.1	0.64	181	181
MAP4	0.0069	1.6	0.0074	182	182
MAST2	0.0039	1.7	0.0043	182	182
MATN2	0.55	0.9	0.55	182	182
MCM6	0.0041	1.7	0.0045	181	181
MICA	0.15	1.3	0.15	182	182
MYH14	0.24	1.2	0.24	182	182
NAA30	0.22	1.2	0.23	180	181
NID1	0.0052	1.6	0.0057	182	182
NRP2	0.41	1.2	0.41	182	182
NSD1	0.48	1.1	0.48	181	180
PALM3	0.42	0.87	0.42	182	182
PAQR6	0.99	1.0	0.99	182	182
PCNX	0.79	0.96	0.8	181	181
PDE4DIP	0.28	1.2	0.28	182	182
PIAS1	0.067	1.4	0.069	182	182
PIK3CG	0.89	0.98	0.9	181	170
PPAP2B	0.16	0.78	0.16	182	182
PPM1D	0.018	1.5	0.018	182	182
PPP2R5C	0.12	1.3	0.12	182	182
PRMT7	0.81	1.0	0.81	182	182
PRR5	0.67	1.1	0.67	182	182
PTPRC	0.93	0.99	0.94	182	182
PTPRJ	0.88	1.0	0.87	182	182
PURA	0.99	1.0	1	181	180
REEP3	0.025	1.5	0.025	182	182
RGS10	0.062	1.4	0.063	182	182
RNF220	0.02	1.5	0.021	182	182
RWDD1	0.02	1.5	0.021	181	181
SAMD11	0.74	0.94	0.74	182	182
SATB2	0.99	1.0	0.99	180	181
SCAMP1	0.35	1.2	0.35	182	182

SIPA1L1	0.96	1.0	0.96	182	182
SKI	0.55	1.1	0.54	182	182
SLC25A27	0.75	1.1	0.75	181	181
SMARCC1	0.011	1.6	0.012	182	182
SORBS1	0.29	1.2	0.29	182	182
SPG11	0.44	1.1	0.44	181	181
SPN	0.22	0.81	0.22	182	182
STRN4	0.0072	1.6	0.0078	182	182
STX1A	0.0018	1.7	0.002	181	181
STX3	0.00097	1.8	0.0011	182	182
SWAP70	0.026	1.5	0.027	182	182
THRSP	0.064	0.72	0.066	182	182
THSD7A	0.18	1.3	0.19	178	177
TIGD1	0.096	1.3	0.1	182	182
TMEM18	0.027	1.5	0.028	182	182
TNS1	0.12	1.3	0.12	182	182
TSC2	0.71	1.1	0.7	182	182
UCN	0.039	1.4	0.039	181	181
UGT2B15	0.18	0.79	0.19	182	182
USP34	0.028	1.5	0.029	182	182
WDR82	0.074	1.4	0.074	182	182
WNT7A	N/A	N/A	N/A	N/A	N/A
ZDHHC7	0.0048	1.7	0.0053	181	181
ZIC5	0.21	1.2	0.21	181	181
ZMIZ2	0.11	1.3	0.12	182	182
ZNF212	0.037	1.5	0.037	182	182
ZNF366	0.096	0.74	0.099	179	181
ZNF540	0.18	0.79	0.18	178	180
ZNF876P	0.9	0.98	0.89	178	168
ZYG11A	0.34	1.2	0.34	179	178

ANO10 in LIHC (n=371):

Model: Surv(OS, EVENT) ~ `ANO10` + Age + Gender + Race + Stage + Purity

310 patients with 106 dying (61 missing obs.)

	coef	HR	se(coef)	95%CI_l	95%CI_u	z	p	signif
ANO10	0.321	1.378	0.133	1.062	1.788	2.414	0.016	*
Age	0.011	1.011	0.008	0.995	1.028	1.367	0.172	
Gendermale	-0.112	0.894	0.226	0.573	1.393	-0.496	0.620	
RaceBlack	0.655	1.924	0.495	0.729	5.080	1.321	0.186	
RaceWhite	-0.025	0.976	0.239	0.611	1.558	-0.104	0.917	
Stage2	0.205	1.227	0.266	0.728	2.068	0.769	0.442	
Stage3	0.910	2.483	0.236	1.563	3.946	3.850	0.000	***
Stage4	1.480	4.392	0.622	1.297	14.876	2.378	0.017	*
Purity	0.706	2.027	0.457	0.827	4.966	1.545	0.122	

Rsquare = 0.101 (max possible = 9.66e-01)
Likelihood ratio test p = 1.3e-04
Wald test p = 8.29e-05
Score (logrank) test p = 3.03e-05

ATG4B in LIHC (n=371):

Model: Surv(OS, EVENT) ~ `ATG4B` + Age + Gender + Race + Stage + Purity

310 patients with 106 dying (61 missing obs.)

	coef	HR	se(coef)	95%CI_l	95%CI_u	z	p	signif
ATG4B	0.350	1.419	0.191	0.976	2.063	1.833	0.067	
Age	0.011	1.011	0.008	0.995	1.028	1.396	0.163	
Gendermale	-0.021	0.979	0.236	0.616	1.555	-0.091	0.928	
RaceBlack	0.838	2.312	0.492	0.881	6.067	1.702	0.089	
RaceWhite	0.016	1.016	0.237	0.639	1.615	0.067	0.947	
Stage2	0.301	1.351	0.262	0.809	2.257	1.150	0.250	
Stage3	0.916	2.500	0.234	1.579	3.958	3.909	0.000	***
Stage4	1.648	5.194	0.619	1.543	17.490	2.660	0.008	**
Purity	0.512	1.669	0.458	0.681	4.092	1.119	0.263	

Rsquare = 0.095 (max possible = 9.66e-01)
Likelihood ratio test p = 3.22e-04
Wald test p = 2.08e-04
Score (logrank) test p = 7.18e-05

ATP11A in LIHC (n=371):

Model: Surv(OS, EVENT) ~ `ATP11A` + Age + Gender + Race + Stage +
Purity

310 patients with 106 dying (61 missing obs.)

	coef	HR	se(coef)	95%CI_l	95%CI_u	z	p	signif
ATP11A	0.215	1.240	0.108	1.004	1.532	1.999	0.046	*
Age	0.015	1.015	0.008	0.998	1.031	1.754	0.080	
Gendermale	-0.061	0.941	0.230	0.599	1.477	-0.265	0.791	
RaceBlack	0.852	2.344	0.491	0.895	6.141	1.734	0.083	
RaceWhite	-0.026	0.974	0.238	0.612	1.552	-0.109	0.913	
Stage2	0.294	1.342	0.262	0.804	2.242	1.125	0.261	
Stage3	0.910	2.484	0.236	1.566	3.942	3.863	0.000	***
Stage4	1.814	6.134	0.630	1.784	21.089	2.879	0.004	**
Purity	0.523	1.686	0.463	0.680	4.181	1.128	0.259	

Rsquare = 0.096 (max possible = 9.66e-01)
Likelihood ratio test p = 2.6e-04
Wald test p = 1.54e-04
Score (logrank) test p = 5.69e-05

ATP6V0A1 in LIHC (n=371):

Model: Surv(OS, EVENT) ~ `ATP6V0A1` + Age + Gender + Race + Stage +
Purity

310 patients with 106 dying (61 missing obs.)

	coef	HR	se(coef)	95%CI_l	95%CI_u	z	p	signif
ATP6V0A1	0.198	1.219	0.157	0.896	1.657	1.262	0.207	

Age	0.010	1.010	0.008	0.994	1.026	1.258	0.208	
Gendermale	-0.166	0.847	0.226	0.544	1.320	-0.732	0.464	
RaceBlack	0.895	2.447	0.489	0.938	6.383	1.830	0.067	
RaceWhite	0.049	1.050	0.239	0.658	1.677	0.205	0.838	
Stage2	0.279	1.322	0.263	0.790	2.212	1.062	0.288	
Stage3	0.919	2.507	0.234	1.583	3.969	3.920	0.000	***
Stage4	1.339	3.817	0.651	1.067	13.660	2.059	0.039	*
Purity	0.656	1.927	0.468	0.770	4.824	1.402	0.161	

Rsquare = 0.089 (max possible = 9.66e-01)
Likelihood ratio test p = 6.55e-04
Wald test p = 4.13e-04
Score (logrank) test p = 1.49e-04

B3GNT9 in LIHC (n=371):
Model: Surv(OS, EVENT) ~ `B3GNT9` + Age + Gender + Race + Stage + Purity
310 patients with 106 dying (61 missing obs.)

	coef	HR	se(coef)	95%CI_l	95%CI_u	z	p	signif
B3GNT9	0.261	1.299	0.110	1.047	1.611	2.381	0.017	*
Age	0.013	1.013	0.008	0.997	1.029	1.566	0.117	
Gendermale	-0.060	0.942	0.228	0.603	1.472	-0.263	0.792	
RaceBlack	0.870	2.387	0.489	0.916	6.220	1.781	0.075	
RaceWhite	-0.025	0.975	0.237	0.613	1.550	-0.106	0.916	
Stage2	0.314	1.368	0.262	0.819	2.288	1.196	0.232	
Stage3	0.887	2.427	0.237	1.524	3.865	3.733	0.000	***
Stage4	1.550	4.711	0.620	1.398	15.869	2.501	0.012	*
Purity	0.929	2.532	0.483	0.983	6.522	1.924	0.054	

Rsquare = 0.1 (max possible = 9.66e-01)
Likelihood ratio test p = 1.57e-04
Wald test p = 7.32e-05
Score (logrank) test p = 2.58e-05

BMP8A in LIHC (n=371):
Model: Surv(OS, EVENT) ~ `BMP8A` + Age + Gender + Race + Stage + Purity
310 patients with 106 dying (61 missing obs.)

	coef	HR	se(coef)	95%CI_l	95%CI_u	z	p	signif
BMP8A	-0.015	0.985	0.179	0.693	1.399	-0.086	0.932	
Age	0.011	1.011	0.008	0.995	1.027	1.330	0.184	
Gendermale	-0.145	0.865	0.232	0.549	1.364	-0.623	0.533	
RaceBlack	0.891	2.439	0.489	0.935	6.359	1.823	0.068	
RaceWhite	0.004	1.004	0.237	0.630	1.598	0.015	0.988	
Stage2	0.315	1.370	0.261	0.820	2.286	1.203	0.229	
Stage3	0.952	2.591	0.237	1.629	4.122	4.022	0.000	***
Stage4	1.593	4.921	0.619	1.463	16.553	2.575	0.010	*
Purity	0.576	1.779	0.458	0.725	4.365	1.258	0.208	

Rsquare = 0.085 (max possible = 9.66e-01)
Likelihood ratio test p = 1.2e-03
Wald test p = 7.25e-04
Score (logrank) test p = 2.74e-04

CAMTA1 in LIHC (n=371):
Model: Surv(OS, EVENT) ~ `CAMTA1` + Age + Gender + Race + Stage + Purity
310 patients with 106 dying (61 missing obs.)

	coef	HR	se(coef)	95%CI_l	95%CI_u	z	p	signif
CAMTA1	0.274	1.316	0.160	0.962	1.799	1.716	0.086	
Age	0.013	1.013	0.008	0.997	1.030	1.562	0.118	
Gendermale	-0.153	0.858	0.225	0.553	1.334	-0.679	0.497	
RaceBlack	0.741	2.098	0.495	0.795	5.537	1.497	0.135	
RaceWhite	-0.028	0.972	0.238	0.610	1.549	-0.120	0.905	

Stage2	0.290	1.336	0.262	0.800	2.230	1.107	0.268	
Stage3	0.889	2.433	0.238	1.524	3.882	3.728	0.000	***
Stage4	1.616	5.032	0.619	1.495	16.932	2.610	0.009	**
Purity	0.356	1.427	0.470	0.568	3.587	0.756	0.450	

Rsquare = 0.093 (max possible = 9.66e-01)
Likelihood ratio test p = 3.83e-04
Wald test p = 2.15e-04
Score (logrank) test p = 7.31e-05

CCR5 in LIHC (n=371):
Model: Surv(OS, EVENT) ~ `CCR5` + Age + Gender + Race + Stage + Purity
310 patients with 106 dying (61 missing obs.)

	coef	HR	se(coef)	95%CI_l	95%CI_u	z	p	signif
CCR5	0.069	1.071	0.143	0.809	1.419	0.481	0.630	
Age	0.011	1.011	0.008	0.995	1.027	1.343	0.179	
Gendermale	-0.124	0.884	0.228	0.566	1.381	-0.543	0.587	
RaceBlack	0.872	2.392	0.491	0.914	6.261	1.776	0.076	
RaceWhite	0.002	1.002	0.237	0.630	1.594	0.008	0.994	
Stage2	0.318	1.374	0.261	0.824	2.293	1.217	0.223	
Stage3	0.956	2.600	0.235	1.640	4.123	4.063	0.000	***
Stage4	1.614	5.025	0.621	1.489	16.959	2.601	0.009	**
Purity	0.690	1.993	0.517	0.724	5.488	1.334	0.182	

Rsquare = 0.085 (max possible = 9.66e-01)
Likelihood ratio test p = 1.11e-03
Wald test p = 6.66e-04
Score (logrank) test p = 2.51e-04

COPA in LIHC (n=371):
Model: Surv(OS, EVENT) ~ `COPA` + Age + Gender + Race + Stage + Purity
310 patients with 106 dying (61 missing obs.)

	coef	HR	se(coef)	95%CI_l	95%CI_u	z	p	signif
COPA	0.312	1.367	0.125	1.069	1.747	2.495	0.013	*
Age	0.014	1.014	0.008	0.997	1.030	1.646	0.100	
Gendermale	-0.072	0.930	0.228	0.595	1.455	-0.317	0.751	
RaceBlack	0.867	2.379	0.495	0.902	6.272	1.752	0.080	
RaceWhite	0.079	1.082	0.238	0.679	1.725	0.331	0.741	
Stage2	0.225	1.252	0.264	0.746	2.101	0.850	0.395	
Stage3	0.927	2.528	0.234	1.597	4.000	3.961	0.000	***
Stage4	1.838	6.285	0.628	1.835	21.520	2.927	0.003	**
Purity	0.460	1.585	0.461	0.642	3.913	0.998	0.318	

Rsquare = 0.104 (max possible = 9.66e-01)
Likelihood ratio test p = 9.02e-05
Wald test p = 6.52e-05
Score (logrank) test p = 2.4e-05

LPP in LIHC (n=371):
Model: Surv(OS, EVENT) ~ `LPP` + Age + Gender + Race + Stage + Purity
310 patients with 106 dying (61 missing obs.)

	coef	HR	se(coef)	95%CI_l	95%CI_u	z	p	signif
LPP	0.157	1.170	0.184	0.816	1.679	0.855	0.392	
Age	0.012	1.012	0.008	0.996	1.028	1.457	0.145	
Gendermale	-0.122	0.885	0.226	0.568	1.379	-0.541	0.589	
RaceBlack	0.882	2.416	0.490	0.926	6.308	1.802	0.072	
RaceWhite	-0.037	0.963	0.242	0.600	1.548	-0.154	0.878	
Stage2	0.303	1.353	0.261	0.811	2.259	1.157	0.247	
Stage3	0.921	2.512	0.238	1.577	4.002	3.877	0.000	***
Stage4	1.652	5.219	0.623	1.539	17.693	2.653	0.008	**
Purity	0.590	1.804	0.457	0.736	4.422	1.290	0.197	

Rsquare = 0.087 (max possible = 9.66e-01)

Likelihood ratio test p = 9.16e-04
Wald test p = 5.39e-04
Score (logrank) test p = 2.05e-04

MAP4 in LIHC (n=371):
Model: Surv(OS, EVENT) ~ `MAP4` + Age + Gender + Race + Stage + Purity
310 patients with 106 dying (61 missing obs.)

	coef	HR	se(coef)	95%CI_l	95%CI_u	z	p	signif
MAP4	0.354	1.425	0.169	1.023	1.985	2.094	0.036	*
Age	0.012	1.012	0.008	0.996	1.028	1.455	0.146	
Gendermale	-0.127	0.881	0.227	0.565	1.373	-0.561	0.575	
RaceBlack	0.899	2.457	0.488	0.943	6.397	1.841	0.066	
RaceWhite	0.030	1.031	0.238	0.647	1.642	0.127	0.899	
Stage2	0.225	1.252	0.265	0.745	2.104	0.850	0.396	
Stage3	0.834	2.303	0.240	1.438	3.688	3.474	0.001	**
Stage4	1.494	4.453	0.620	1.320	15.025	2.407	0.016	*
Purity	0.664	1.942	0.459	0.790	4.774	1.447	0.148	

Rsquare = 0.097 (max possible = 9.66e-01)
Likelihood ratio test p = 2.21e-04
Wald test p = 1.49e-04
Score (logrank) test p = 4.71e-05

MAST2 in LIHC (n=371):
Model: Surv(OS, EVENT) ~ `MAST2` + Age + Gender + Race + Stage + Purity
310 patients with 106 dying (61 missing obs.)

	coef	HR	se(coef)	95%CI_l	95%CI_u	z	p	signif
MAST2	0.433	1.542	0.138	1.176	2.021	3.134	0.002	**
Age	0.012	1.012	0.008	0.996	1.029	1.499	0.134	
Gendermale	0.005	1.005	0.232	0.637	1.584	0.021	0.983	
RaceBlack	0.944	2.570	0.492	0.980	6.743	1.918	0.055	
RaceWhite	0.067	1.070	0.238	0.671	1.705	0.284	0.776	
Stage2	0.157	1.170	0.267	0.693	1.976	0.588	0.556	
Stage3	0.839	2.314	0.238	1.452	3.687	3.530	0.000	***
Stage4	1.674	5.333	0.622	1.576	18.046	2.692	0.007	**
Purity	0.440	1.553	0.454	0.638	3.779	0.969	0.332	

Rsquare = 0.113 (max possible = 9.66e-01)
Likelihood ratio test p = 2.42e-05
Wald test p = 1.2e-05
Score (logrank) test p = 4.08e-06

MCM6 in LIHC (n=371):
Model: Surv(OS, EVENT) ~ `MCM6` + Age + Gender + Race + Stage + Purity
310 patients with 106 dying (61 missing obs.)

	coef	HR	se(coef)	95%CI_l	95%CI_u	z	p	signif
MCM6	0.417	1.517	0.105	1.234	1.864	3.964	0.000	***
Age	0.016	1.016	0.008	0.999	1.033	1.850	0.064	
Gendermale	0.061	1.063	0.236	0.669	1.690	0.259	0.796	
RaceBlack	0.643	1.902	0.509	0.701	5.161	1.263	0.207	
RaceWhite	0.125	1.134	0.244	0.703	1.828	0.515	0.607	
Stage2	0.131	1.140	0.270	0.672	1.934	0.485	0.628	
Stage3	0.759	2.136	0.241	1.333	3.424	3.153	0.002	**
Stage4	1.918	6.805	0.629	1.982	23.367	3.047	0.002	**
Purity	0.324	1.383	0.459	0.563	3.399	0.707	0.480	

Rsquare = 0.13 (max possible = 9.66e-01)
Likelihood ratio test p = 2.14e-06
Wald test p = 1.59e-06
Score (logrank) test p = 4.46e-07

NID1 in LIHC (n=371):
Model: Surv(OS, EVENT) ~ `NID1` + Age + Gender + Race + Stage + Purity

310 patients with 106 dying (61 missing obs.)

	coef	HR	se(coef)	95%CI_l	95%CI_u	z	p	signif
NID1	0.246	1.279	0.094	1.063	1.539	2.608	0.009	**
Age	0.017	1.017	0.009	1.000	1.034	1.944	0.052	
Gendermale	-0.022	0.978	0.234	0.619	1.546	-0.095	0.925	
RaceBlack	0.890	2.434	0.491	0.931	6.367	1.814	0.070	
RaceWhite	-0.020	0.981	0.238	0.615	1.564	-0.082	0.934	
Stage2	0.312	1.366	0.263	0.816	2.286	1.185	0.236	
Stage3	0.930	2.536	0.235	1.601	4.016	3.966	0.000	***
Stage4	1.920	6.823	0.634	1.968	23.658	3.027	0.002	**
Purity	0.422	1.525	0.455	0.625	3.722	0.927	0.354	

Rsquare = 0.105 (max possible = 9.66e-01)
Likelihood ratio test p = 7.52e-05
Wald test p = 5.37e-05
Score (logrank) test p = 1.78e-05

REEP3 in LIHC (n=371):
Model: Surv(OS, EVENT) ~ `REEP3` + Age + Gender + Race + Stage + Purity
310 patients with 106 dying (61 missing obs.)

	coef	HR	se(coef)	95%CI_l	95%CI_u	z	p	signif
REEP3	0.229	1.258	0.147	0.942	1.679	1.554	0.120	
Age	0.013	1.013	0.008	0.997	1.030	1.584	0.113	
Gendermale	-0.111	0.895	0.227	0.573	1.397	-0.489	0.625	
RaceBlack	0.943	2.568	0.491	0.982	6.717	1.923	0.055	
RaceWhite	-0.063	0.939	0.242	0.585	1.508	-0.260	0.795	
Stage2	0.286	1.332	0.262	0.797	2.224	1.095	0.274	
Stage3	0.894	2.445	0.239	1.532	3.902	3.748	0.000	***
Stage4	1.657	5.244	0.620	1.555	17.689	2.671	0.008	**
Purity	0.537	1.711	0.460	0.694	4.216	1.166	0.244	

Rsquare = 0.092 (max possible = 9.66e-01)
Likelihood ratio test p = 4.78e-04
Wald test p = 2.38e-04
Score (logrank) test p = 8.85e-05

RNF220 in LIHC (n=371):
Model: Surv(OS, EVENT) ~ `RNF220` + Age + Gender + Race + Stage + Purity
310 patients with 106 dying (61 missing obs.)

	coef	HR	se(coef)	95%CI_l	95%CI_u	z	p	signif
RNF220	0.592	1.808	0.190	1.245	2.626	3.111	0.002	**
Age	0.011	1.011	0.008	0.995	1.028	1.368	0.171	
Gendermale	-0.035	0.965	0.230	0.615	1.516	-0.153	0.879	
RaceBlack	0.789	2.202	0.493	0.838	5.788	1.601	0.109	
RaceWhite	0.133	1.142	0.241	0.713	1.831	0.553	0.580	
Stage2	0.211	1.235	0.265	0.735	2.074	0.796	0.426	
Stage3	0.820	2.271	0.238	1.423	3.625	3.440	0.001	**
Stage4	1.795	6.022	0.624	1.772	20.461	2.877	0.004	**
Purity	0.441	1.554	0.453	0.639	3.777	0.972	0.331	

Rsquare = 0.113 (max possible = 9.66e-01)
Likelihood ratio test p = 2.58e-05
Wald test p = 1.65e-05
Score (logrank) test p = 5.63e-06

SMARCC1 in LIHC (n=371):
Model: Surv(OS, EVENT) ~ `SMARCC1` + Age + Gender + Race + Stage + Purity
310 patients with 106 dying (61 missing obs.)

	coef	HR	se(coef)	95%CI_l	95%CI_u	z	p	signif
SMARCC1	0.408	1.503	0.143	1.136	1.989	2.853	0.004	**
Age	0.015	1.016	0.008	0.999	1.032	1.874	0.061	

Gendermale	-0.085	0.918	0.228	0.588	1.435	-0.374	0.709	
RaceBlack	0.759	2.137	0.501	0.801	5.701	1.517	0.129	
RaceWhite	0.075	1.078	0.238	0.676	1.721	0.316	0.752	
Stage2	0.230	1.258	0.264	0.749	2.112	0.869	0.385	
Stage3	0.887	2.429	0.236	1.531	3.854	3.766	0.000	***
Stage4	1.744	5.718	0.623	1.686	19.387	2.799	0.005	**
Purity	0.449	1.567	0.461	0.635	3.865	0.975	0.330	

Rsquare = 0.109 (max possible = 9.66e-01)
Likelihood ratio test p = 4.44e-05
Wald test p = 3.11e-05
Score (logrank) test p = 1.06e-05

STRN4 in LIHC (n=371):
Model: Surv(OS, EVENT) ~ `STRN4` + Age + Gender + Race + Stage + Purity
310 patients with 106 dying (61 missing obs.)

	coef	HR	se(coef)	95%CI_l	95%CI_u	z	p	signif
STRN4	0.360	1.433	0.154	1.059	1.939	2.330	0.020	*
Age	0.013	1.013	0.008	0.997	1.030	1.587	0.112	
Gendermale	-0.002	0.998	0.235	0.630	1.583	-0.007	0.994	
RaceBlack	0.791	2.206	0.493	0.839	5.797	1.604	0.109	
RaceWhite	0.019	1.019	0.237	0.640	1.622	0.080	0.936	
Stage2	0.248	1.282	0.264	0.763	2.151	0.939	0.348	
Stage3	0.915	2.497	0.234	1.578	3.951	3.909	0.000	***
Stage4	1.675	5.339	0.622	1.579	18.051	2.695	0.007	**
Purity	0.561	1.753	0.455	0.719	4.275	1.234	0.217	

Rsquare = 0.1 (max possible = 9.66e-01)
Likelihood ratio test p = 1.46e-04
Wald test p = 1.11e-04
Score (logrank) test p = 3.93e-05

STX1A in LIHC (n=371):
Model: Surv(OS, EVENT) ~ `STX1A` + Age + Gender + Race + Stage + Purity
310 patients with 106 dying (61 missing obs.)

	coef	HR	se(coef)	95%CI_l	95%CI_u	z	p	signif
STX1A	0.385	1.470	0.121	1.159	1.865	3.177	0.001	**
Age	0.010	1.010	0.008	0.994	1.026	1.236	0.217	
Gendermale	-0.116	0.891	0.225	0.573	1.384	-0.514	0.607	
RaceBlack	0.938	2.555	0.490	0.977	6.678	1.913	0.056	
RaceWhite	-0.030	0.971	0.240	0.606	1.554	-0.124	0.901	
Stage2	0.288	1.333	0.261	0.799	2.225	1.100	0.271	
Stage3	0.865	2.374	0.238	1.490	3.781	3.640	0.000	***
Stage4	1.763	5.830	0.622	1.724	19.712	2.837	0.005	**
Purity	0.585	1.795	0.468	0.717	4.497	1.249	0.212	

Rsquare = 0.108 (max possible = 9.66e-01)
Likelihood ratio test p = 4.71e-05
Wald test p = 1.65e-05
Score (logrank) test p = 3.91e-06

STX3 in LIHC (n=371):
Model: Surv(OS, EVENT) ~ `STX3` + Age + Gender + Race + Stage + Purity
310 patients with 106 dying (61 missing obs.)

	coef	HR	se(coef)	95%CI_l	95%CI_u	z	p	signif
STX3	0.320	1.377	0.101	1.130	1.677	3.176	0.001	**
Age	0.012	1.013	0.008	0.996	1.029	1.501	0.133	
Gendermale	-0.002	0.998	0.232	0.633	1.575	-0.007	0.994	
RaceBlack	0.978	2.659	0.492	1.014	6.972	1.988	0.047	*
RaceWhite	0.011	1.011	0.240	0.631	1.619	0.045	0.964	
Stage2	0.262	1.300	0.263	0.776	2.177	0.998	0.318	
Stage3	0.844	2.325	0.239	1.456	3.711	3.536	0.000	***

Stage4	1.590	4.906	0.621	1.451	16.581	2.560	0.010	*
Purity	0.771	2.161	0.469	0.862	5.418	1.643	0.100	

Rsquare = 0.113 (max possible = 9.66e-01)
Likelihood ratio test p = 2.57e-05
Wald test p = 1.26e-05
Score (logrank) test p = 3.84e-06

SWAP70 in LIHC (n=371):
Model: Surv(OS, EVENT) ~ `SWAP70` + Age + Gender + Race + Stage + Purity
310 patients with 106 dying (61 missing obs.)

	coef	HR	se(coef)	95%CI_l	95%CI_u	z	p	signif
SWAP70	0.282	1.326	0.119	1.049	1.675	2.360	0.018	*
Age	0.013	1.013	0.008	0.997	1.030	1.610	0.107	
Gendermale	-0.032	0.969	0.231	0.616	1.525	-0.137	0.891	
RaceBlack	0.859	2.361	0.491	0.902	6.184	1.749	0.080	
RaceWhite	-0.025	0.976	0.237	0.613	1.553	-0.104	0.917	
Stage2	0.260	1.297	0.263	0.775	2.171	0.990	0.322	
Stage3	0.933	2.542	0.235	1.603	4.030	3.968	0.000	***
Stage4	1.602	4.962	0.620	1.471	16.741	2.581	0.010	*
Purity	0.722	2.059	0.464	0.830	5.108	1.558	0.119	

Rsquare = 0.101 (max possible = 9.66e-01)
Likelihood ratio test p = 1.35e-04
Wald test p = 7.97e-05
Score (logrank) test p = 2.97e-05

UCN in LIHC (n=371):
Model: Surv(OS, EVENT) ~ `UCN` + Age + Gender + Race + Stage + Purity
310 patients with 106 dying (61 missing obs.)

	coef	HR	se(coef)	95%CI_l	95%CI_u	z	p	signif
UCN	0.123	1.131	0.148	0.846	1.513	0.833	0.405	
Age	0.010	1.010	0.008	0.994	1.026	1.226	0.220	
Gendermale	-0.165	0.848	0.226	0.544	1.321	-0.728	0.467	
RaceBlack	0.849	2.337	0.491	0.892	6.123	1.728	0.084	
RaceWhite	-0.008	0.992	0.237	0.624	1.578	-0.032	0.974	
Stage2	0.300	1.350	0.262	0.808	2.256	1.145	0.252	
Stage3	0.932	2.540	0.236	1.600	4.031	3.956	0.000	***
Stage4	1.643	5.173	0.622	1.529	17.498	2.643	0.008	**
Purity	0.534	1.706	0.460	0.692	4.205	1.160	0.246	

Rsquare = 0.087 (max possible = 9.66e-01)
Likelihood ratio test p = 9.29e-04
Wald test p = 5.49e-04
Score (logrank) test p = 2.04e-04

ZNF212 in LIHC (n=371):
Model: Surv(OS, EVENT) ~ `ZNF212` + Age + Gender + Race + Stage + Purity
310 patients with 106 dying (61 missing obs.)

	coef	HR	se(coef)	95%CI_l	95%CI_u	z	p	signif
ZNF212	0.228	1.255	0.201	0.847	1.860	1.135	0.257	
Age	0.011	1.011	0.008	0.995	1.027	1.330	0.183	
Gendermale	-0.129	0.879	0.226	0.564	1.370	-0.569	0.570	
RaceBlack	0.850	2.341	0.492	0.892	6.142	1.728	0.084	
RaceWhite	0.034	1.035	0.238	0.649	1.650	0.143	0.886	
Stage2	0.312	1.366	0.262	0.818	2.281	1.192	0.233	
Stage3	0.915	2.496	0.236	1.571	3.964	3.875	0.000	***
Stage4	1.665	5.286	0.623	1.560	17.912	2.674	0.007	**
Purity	0.453	1.574	0.470	0.627	3.951	0.965	0.334	

Rsquare = 0.088 (max possible = 9.66e-01)

```
Likelihood ratio test p = 7.32e-04
Wald test p = 4.4e-04
Score (logrank) test p = 1.63e-04
```

Supplemental Table 13: Clinico-pathological data of patients (validation cohort Mainz)

Characteristic	low (≤ 0.162516)	high (> 0.162516)	p-value[2]
	N = 372 (71%)[1]	N = 149 (29%)[1]	
Median age in years (range)	64.9 (56.6, 71.0)	64.0 (55.7, 71.3)	0.5
Gender			0.9
Male	294 (79%)	116 (78%)	
Female	78 (21%)	33 (22%)	
Etiology of liver disease			
Alcohol abuse	104 (28%)	51 (34%)	0.2
HCV	80 (22%)	24 (16%)	0.2
HBV	71 (19%)	24 (16%)	0.5
NASH	34 (9.1%)	7 (4.7%)	0.13
Hemochromatosis	19 (5.1%)	5 (3.4%)	0.5
BCLC			>0.9
A	66 (18%)	24 (16%)	
B	242 (65%)	97 (65%)	
C	43 (12%)	18 (12%)	
D	21 (5.6%)	10 (6.7%)	
ECOG PST			0.3
0-1	359 (97%)	142 (96%)	
2	4 (1.1%)	4 (2.7%)	
3	4 (1.1%)	0 (0%)	
4	3 (0.8%)	2 (1.4%)	
Liver cirrhosis			0.5
Absent	133 (36%)	59 (40%)	
Present	239 (64%)	90 (60%)	
Child-Pugh-Score			0.7
A	140 (54%)	48 (50%)	
B	97 (38%)	38 (40%)	
C	21 (8.1%)	10 (10%)	
Portal vein thrombosis (PVT)	46 (12%)	18 (12%)	>0.9
Vascular invasion			>0.9
Absent	239 (64%)	94 (63%)	
Micro	75 (20%)	32 (21%)	
Macro	58 (16%)	23 (15%)	

[1] Statistics presented: median (IQR); n (%)

[2] Statistical tests performed: Wilcoxon rank-sum test; chi-square test of independence; Fisher's exact test

Supplemental Table 14: Large and small liver cell changes

Patient	Pathology number	Large cell change_grade	Small cell change_grade
1	05-28637	2	1
2	07-07906	3	2
3	08-24098	3	3
4	08-32197	1	1
5	08-40746	3	2
6	09-27796	3	1
7	09-09377	4	2
8	11-44812	3	2

Criteria	grade 1	1~5%
	grade2	6~25%
	grade 3	26~50%
	grade 4	51~100%
